# Tools to guide clinical discussions on physical activity, sedentary behaviour, and/or sleep for health promotion between primary care providers and adults accessing care: a scoping review

**DOI:** 10.1186/s12875-023-02091-9

**Published:** 2023-07-07

**Authors:** Tamara L. Morgan, Emma Faught, Amanda Ross-White, Michelle S. Fortier, Mary Duggan, Rahul Jain, Kirstin N. Lane, Amanda Lorbergs, Kaleigh Maclaren, Taylor McFadden, Jennifer R. Tomasone

**Affiliations:** 1 School of Kinesiology and Health Studies, Queen’s University, 28 Division Street, Kingston, ON K7L 3N6 Canada; 2 School of Medicine, Queen’s University, Kingston, ON Canada; 3 Bracken Health Sciences Library, Queen’s University, Kingston, ON Canada; 4 School of Human Kinetics, University of Ottawa, Ottawa, ON Canada; 5 Canadian Society for Exercise Physiology, Ottawa, ON Canada; 6 Temerty Faculty of Medicine, University of Toronto, Toronto, ON Canada; 7 School of Exercise Science, Physical and Health Education, University of Victoria, Victoria, BC Canada; 8 Canadian Frailty Network, Kingston, ON Canada; 9 Independent Communication Specialist, Ottawa, ON Canada; 10 Canadian Medical Association, Ottawa, ON Canada

**Keywords:** Physical activity, Sedentary behaviour, Sleep, 24-Hour Movement Guidelines, Primary care, Assessment, Counselling, Prescription, Referral, Tool

## Abstract

**Background:**

Health care providers have reported low knowledge, skill, and confidence for discussing movement behaviours (i.e., physical activity, sedentary behaviour, and sleep), which may be improved with the use of tools to guide movement behaviour discussions in their practice. Past reviews have examined the psychometric properties, scoring, and behavioural outcomes of physical activity discussion tools. However, the features, perceptions, and effectiveness of discussion tools for physical activity, sedentary behaviour, and/or sleep have not yet been synthesized. The aim of this review was to report and appraise tools for movement behaviour discussions between health care providers and adults 18 + years in a primary care context within Canada or analogous countries.

**Methods:**

An integrated knowledge translation approach guided this review, whereby a working group of experts in medicine, knowledge translation, communications, kinesiology, and health promotion was engaged from research question formation to interpretation of findings. Three search approaches were used (i.e., peer-reviewed, grey literature, and forward searches) to identify studies reporting on perceptions and/or effectiveness of tools for physical activity, sedentary behaviour, and/or sleep. The quality of included studies was assessed using the Mixed Methods Appraisal Tool.

**Results:**

In total, 135 studies reporting on 61 tools (i.e., 51 on physical activity, one on sleep, and nine combining two movement behaviours) met inclusion criteria. Included tools served the purposes of assessment (*n* = 57), counselling (*n* = 50), prescription (*n* = 18), and/or referral (*n* = 12) of one or more movement behaviour. Most tools were used or intended for use by physicians, followed by nurses/nurse practitioners (*n* = 11), and adults accessing care (*n* = 10). Most tools were also used or intended to be used with adults without chronic conditions aged 18–64 years (*n* = 34), followed by adults with chronic conditions (*n* = 18). The quality of the 116 studies that evaluated tool effectiveness varied.

**Conclusions:**

Many tools were positively perceived and were deemed effective at enhancing knowledge of, confidence for, ability in, and frequency of movement behaviour discussions. Future tools should guide discussions of all movement behaviours in an integrated manner in line with the 24-Hour Movement Guidelines. Practically, this review offers seven evidence-based recommendations that may guide future tool development and implementation.

**Supplementary Information:**

The online version contains supplementary material available at 10.1186/s12875-023-02091-9.

## Introduction

The Canadian 24-Hour Movement Guidelines for Adults (24HMG) offer integrated recommendations on physical activity (PA), sedentary behaviour, and sleep for adults [1]. Uniquely, these guidelines emphasize a 24-h movement paradigm, which characterizes optimal patterns of, and interactions between, PA, sedentary behaviour, and sleep [2, 3]. This paradigm has been gaining traction nationally and internationally, such as with health guidelines for some chronic diseases [4]. In a 24-h paradigm, it is recommended to trade sedentary behaviour for light PA or moderate-to-vigorous PA (MVPA), while preserving sufficient sleep [1].^1^ Regrettably, general population adults are largely non-adherent to movement behaviours [5] and are incurring increased morbidity and mortality risk as a result [6].

Health care providers are well-positioned to promote healthy PA, sedentary behaviour, and sleep as they are in semi-frequent contact with general population adults [7] and are deemed reliable sources of health information [8]. Indeed, 74% of Canadian adults met with their provider at least once in 2018 [9]. Moreover, health care providers are appropriate disseminators and implementers of movement behaviour guidelines as primary care covers a spectrum of services devoted to the improvement of health outcomes [10, 11]. In Canada and other developed, high-income countries, primary care is commonly delivered by physicians, nurses, and nurse practitioners [12, 13]; however, family health teams may involve other providers, including pharmacists [12, 14, 15], dietitians [12, 16], psychologists [15], registered psychotherapists [17], and social workers [10, 12], who perform key roles in movement behaviour promotion.

Providers can engage in several actions to promote sufficient sleep, PA, and reduce sedentary behaviour among adults accessing care. For the present study, the terminology “adults accessing care” is used in place of “patients” as it is more inclusive and disaffirms a power dynamic with providers [18]. When engaging in discussions with adults accessing care, providers can *assess* current movement behaviours, *counsel* on behaviour change strategies*, prescribe* targets for behaviour change, and *refer* to other professionals or programs for follow-up [19, 20]. However, providers have reported low knowledge, skill, confidence, and motivation for movement behaviour conversations as well as barriers to movement behaviour promotion, such as lack of remuneration [21–24]. Notably, materials and strategies to support providers have been identified as facilitators to implementing movement behaviour promotion practices [25].

Tools that guide movement behaviour discussions between providers and adults accessing care have been developed and used since the early 2000s (e.g., [26, 27]). Overwhelmingly, these tools have focused on PA, while only some have focused on sleep and few have focused on sedentary behaviour only in combination with PA. New tools have emerged over recent years (e.g., [28]), showing a continued and growing interest in the field. Nevertheless, these single-behaviour tools are limited in their utility to inform discussions on integrated movement behaviours in line with the 24HMG. Thus, considering tools that guide discussions on all three movement behaviours between providers and individuals accessing care is important and timely. Notably, this work could inform the development of new, integrated tools or the refinement of existing tools to promote healthy movement behaviours.

### Rationale

Two systematic reviews [29, 30] and one literature review [31] have examined the psychometric properties, scoring, and behavioural outcomes of PA discussion tools. However, these reviews have captured neither all previously available tools nor tools that have emerged since 2017. Furthermore, tools for sedentary behaviour or sleep discussions for health promotion have not been reviewed. Finally, reviews have not considered whether tools are theory-based or stem from public health guidelines. Implementation efforts grounded in behaviour change theory may procure greater success [32]. Likewise, public health guidelines are developed via systematic review and expert appraisal [33] and their implementation success is strategically monitored and supported by multidisciplinary teams [34]. Lastly, the perceptions of providers and individuals accessing care regarding the utility of movement behaviour discussion tools have not been reviewed, which could inform more practically relevant and useful tools. A scoping review was deemed necessary to build upon the evidence base by capturing a broader range of features and outcomes for a larger number of tools on PA, sedentary behaviour, and sleep for health promotion discussions in primary care**.**

### Objectives

The purpose of this scoping review was to report on and appraise tools that guide movement behaviour discussions between health care providers (i.e., physicians, nurses, nurse practitioners, pharmacists, dietitians, psychologists, registered psychotherapists, and social workers) and adults 18 + years accessing care in a primary care context within Canada or analogous countries (i.e., English-speaking, developed, high-income).

The research questions (RQs) were: (1) What tools are available to guide discussions on assessment, counselling, prescription, and/or referral for PA, sedentary behaviour, and/or sleep for chronic disease prevention and health promotion among adults 18 + years, and what are their features? (2) What are the positive and/or negative perceptions of health care providers and adults accessing care toward the potential and/or actual utility of these tools in clinical interactions? and (3) Has the use of these tools enhanced (i) providers’ knowledge, ability, confidence for, and/or frequency of assessing, counselling, prescribing, and/or referring or (ii) levels of PA, sedentary behaviour, and/or sleep among adults accessing care?

## Methods

Engaging knowledge partners bridges the gap between research and non-research audiences to enhance the applicability, clarity, awareness, and dissemination of review findings [35]. Therefore, an integrated knowledge translation approach [36, 37] was chosen to guide this review. A working group of experts (i.e., academic professionals, health professionals, and representatives of organizations related to the topic of study) were personally invited to be involved from research question formation to interpretation of findings. Working group involvement transpired over three 90-min structured online meetings led by TLM and email correspondence (June-October, 2021). Scoping review methodology was chosen as we aimed to broadly appraise the characteristics of movement behaviour discussion tools where much of the research is emergent and not amenable to examining effectiveness alone [38]. Established guidance for scoping review methodology [39] and quality of reporting on knowledge partner engagement in reviews [40, 41] were followed. An a priori review protocol was registered in Open Science Framework on July 23, 2021 [42] that, like this paper, noted conflicts of interest. Findings are reported in accordance with the Preferred Reporting Items for Systematic Reviews and Meta-Analyses Extension for Scoping Reviews (PRISMA-ScR [43]). The PRISMA-ScR checklist is shown in Additional File 1. Ethics approval was not required for this review.

### Eligibility criteria

Eligible studies reporting on, and sufficiently describing, a tool for assessment, counselling, prescription, and/or referral of PA, sedentary behaviour, and/or sleep used or intended for use among adults 18 + years with or without a chronic disease(s) accessing primary care were considered. Primary care settings were defined as the first point of contact for adults accessing care [11]. Studies must have been published in Canada or a similar country (i.e., English-speaking, developed, high-income) in 2000 or later to be eligible for inclusion. All study designs were eligible, including those reported in conference proceedings, except for scoping reviews, systematic reviews, and meta-analyses. Studies and tools not available in English were excluded and those implemented in a low-to-middle income country were not considered due to the dissimilarity of their health care context compared to that of high-income countries [44].

### Search strategy

Recommendations by Levac and colleagues [45] informed the search strategy. A professional librarian (ARW) developed and carried out the peer-reviewed (MedLine [Ovid], EMBASE [Ovid], PsycINFO [Ovid], CINAHL [Ebsco], Cochrane Controlled Trials Register [Ovid], and Google Scholar [Publish or Perish [46]) and grey literature (Thesis & Dissertations-ProQuest Dissertations Online, Web of Science, Canadian Electronic Library-Canadian Public Policy Collection and Canadian Health Research Collection) searches in June 2021. In addition, forward searches of all studies deemed eligible for full-text screening were performed by TLM in August 2021 to ensure all related references would be captured. Forward searching has been found to be a highly effective method for reviews where concepts are challenging to retrieve using subject headings or keywords [47, 48]. Finally, review authors were asked to check their personal libraries to identify additional, potentially relevant peer-reviewed publications. Additional File 2 contains the full search strategy.

### Study selection

Deduplication of studies occurred in Covidence [49]. Two reviewers (TLM and EF) independently screened all titles and abstracts from the peer-reviewed and grey literature searches. Title and abstract screening of forward search results was performed by TLM. Full-texts deemed as potentially relevant were downloaded and independently screened by the two reviewers. Discrepancies regarding inclusion at the title and abstract and full-text phases were resolved by discussion between the two reviewers, and in consultation with a third reviewer (JRT) when necessary.

### Data extraction

The data extraction table was independently piloted by four researchers (TLM, EF, and two research assistants, LK and MK). All four researchers attempted extraction of three papers and met to reach consensus on the table’s utility for addressing all RQs. Subsequently, the four researchers each extracted one-quarter of included studies prior to auditing another one-quarter (e.g., TLM audited LK, and vice versa). Data was extracted per: study characteristics (i.e., author(s), title, year, study design, country, and participant characteristics), tool characteristics (i.e., RQ1: name, purpose(s), description, format, population(s) used by and served, and guideline and/or theoretical basis), tool perception outcomes as per a modified coding framework by Neudorf and colleagues [50] (i.e., RQ2: satisfaction, content, efficiency, navigation, understandability, usability, visibility, and workflow), and tool effectiveness outcomes (i.e., RQ3: knowledge, ability, confidence, frequency of assessment/counselling/prescription/referral, movement behaviours, and outcomes not stated in RQ3). Neudorf and colleagues’ [50] framework was modified to include an eighth component—tool satisfaction—per the recommendation of a recent systematic review [29]. Applying this framework may help capture a broader understanding of why tools may or may not have been positively rated or effective in practice. Included tools were classified into five formats: paper-based, integrated into the Electronic Medical Record (EMR), electronic-based (i.e., tablet, website, online portal, software/program), mobile-based (i.e., app, text messaging), or pedometer-based.

### Data synthesis

Following data extraction, discussion with the working group was sought to interpret the results. Narrative synthesis of studies reporting on outcomes across all RQs was performed [51] to corroborate positive perceptions of tools with their features and effectiveness outcomes. Study results pointing toward the future improvement of tools were also synthesized. Synthesis of studies examining outcomes across all RQs involved reporting the same language and statistical data used by the authors of the primary studies. Results are presented across four supplementary files and one table included herein: Additional Files 3–5 report tool characteristics (RQ1), including a description of each tool, and are organized alphabetically by tool name; Additional File 6 reports all studies addressing RQ2 and 3 outcomes, organized alphabetically by tool name, then chronologically by study year within each tool; Table 1 shows the links between tool features, perceptions, and outcomes and evidence-based recommendations for future tool development.

**Table 1:** Recommendations for future discussion tools on movement behaviours, based on evidence from primary studies included in the present review

Recommendation	Rationale	Supporting Evidence
1. Tools should, at a minimum, guide assessment and prescription, and if time allows, include counselling and referral for movement behaviours	Some providers may be more familiar with the act of assessing or prescribing [20] whereas counselling typically requires more time and training [52, 53]. Referral is the natural conclusion of a clinical encounter [54–56]	[52–64]
2. Tools should be quick to administer, ideally in three minutes or less	Time is a frequently reported barrier to providers’ facilitation of movement behaviour discussions [23, 53] and effective discussions are possible within 2–5 min [59, 65]	[26, 56, 59, 66–74]
3. Paper-based and electronic tool formats should be prioritized in the initial stages of tool development, with integration into the EMR being a more distal goal	Paper and electronic formats can be used electronically or as handouts [75], are seen as convenient [76], and can be developed at little to no cost compared to EMR integration, which can be more cost-dependent [77]	[54, 76–83]
4. Tools should be aesthetically appealing and have visual representations of concepts (e.g., graphs or progress bars)	Having numerical and graphical information and illustrations can enhance navigation, understandability, and usability [84–86]	[76, 79, 84, 86–92]
5. Tools should include generic statements and recommendations on movement behaviours that can broadly apply to multiple populations of individuals accessing care	Generic statements and recommendations can enhance efficacy when promoting movement behaviours among adult populations [93, 94]. E.g., “move more”, “reduce sedentary time”, and “focus on sleep hygiene”	[14, 28, 56, 95]
6. Tools should be informed by one or more theories, models, and/or frameworks in addition to public health guidelines	Tools informed by theories/models/frameworks and public health guidelines were more likely to be associated with greater implementation success than those not informed by theories/models/frameworks or a public health guideline	[59, 68, 69, 71, 72, 78, 85, 89, 90, 96–103]
7. Tool implementation should be supported by accompanying training and resources	Provider training can increase the likelihood they will counsel on and prescribe movement behaviours [104] and individuals accessing care have reported that take-home printouts are helpful [82]	[14, 28, 82, 101, 104–106]

*EMR* Electronic Medical Record

### Quality assessment

The methodological quality of included studies answering RQ3 (i.e., tool effectiveness) was appraised by TLM, EF, LK, and MK using the Mixed Methods Appraisal Tool (MMAT [107]). The MMAT includes five unique rating criteria for each of five study designs (i.e., randomized controlled trials, non-randomized designs, quantitative descriptive designs, qualitative research, and mixed methods designs). MMAT scores ranged from 0 to 5 out of a possible 5 (see rightmost column in Additional File 6); however, only scores of similar study designs should be compared [108].

## Results

**Fig. 1:**
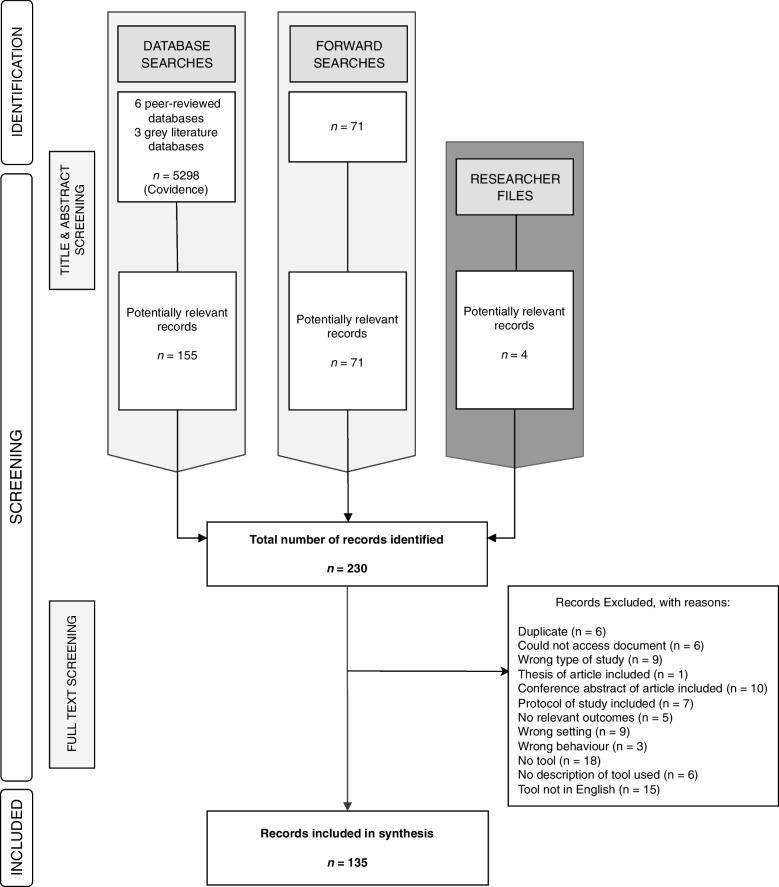
PRISMA figure of study flow

Peer-reviewed and grey literature searches identified 8 292 studies. After de-duplication, 5 298 studies remained. Following title and abstract screening, 155 studies were carried forward to full-text screening. Forward searching and authors’ searching of personal libraries identified another 71 and 4 studies, respectively, resulting in a total of 230 full-texts. In total, 135 studies (i.e., peer-reviewed and grey literature searches, *n* = 77; forward searches, *n* = 55; researchers files, *n* = 3) were included in data synthesis (Fig. 1).

### Tool characteristics

Sixty-one tools were identified, with several tools including more than one purpose, movement behaviour, format, and/or population. Of the 61 tools, 57 were for the purpose of assessment, 50 were for counselling, 18 were for prescription, and 12 were for referral. Assessment was rarely performed in isolation; only five tools were for assessment alone. Counselling was performed once in isolation [77]. Prescription and referral were not performed alone in any included tool. Nine tools that integrated two movement behaviours were identified. Fifty-one tools focused on PA, one focused on sleep, and nine focused on multiple behaviours (i.e., seven on PA/sedentary behaviour, two on PA/sleep). Most tools were paper-based (including PDF; *n* = 33), followed by EMR-based (*n* = 18), electronic-based (e.g., software, website, tablet; *n* = 17), pedometer-based (*n* = 6), mobile-based (e.g., app, text messaging; *n* = 2). Most tools were used or intended for use by physicians (*n* = 49), followed by nurses/nurse practitioners (*n* = 11), adults accessing care (*n* = 10), dietitians (*n* = 3), pharmacists (*n* = 3), and psychologists/social workers (*n* = 1). Many tools were used or intended to be used with adults without chronic conditions aged 18–64 years (*n* = 34) and adults with chronic conditions (*n* = 18); less were used or intended to be used with adults with overweight or obesity specifically (*n* = 10), adults 65 + years (*n* = 5), adults who smoke (*n* = 1), and veterans (*n* = 1).

### Perceptions of tools

Positive perceptions regarding all RQ2 outcomes except navigation were reported for four of the seven PA/sedentary behaviour tools. For example, providers felt the functions of the EMR PA Tool were easy to use, worthwhile, and should be permanently integrated into the EMR [109]. Further, negative perceptions for two RQ2 outcomes (i.e., navigation and visibility) were shared for one tool: nurses reported overlooking certain functional elements (i.e., buttons within individual charts [84]) in the Interactive Tool for Self-management through LIfestyle FEedback! (It’s LiFe!) tool and felt that navigation was too complex and laden with technical issues [84, 110]. Mixed perceptions on five RQ2 outcomes (i.e., satisfaction, content, efficiency, usability, and workflow) were reported for two tools. For instance, adults accessing care valued providers’ use of the Paper-Based Decision Tool; however, providers did not seem to recognize that the tool was valued and found it “impossible to use in everyday practice”[58 p7]. Only one of the two PA/sleep tools reported RQ2 outcomes. Positive perceptions of usability were reported for EMR-based the Integrated Wellness Tool (IWT), wherein adults and providers stated that the tool was easy to use [111]. However, mixed perceptions regarding users’ satisfaction and workflow were also given for the IWT. Most adults reported that the IWT would aid their provider in better understanding their health; however, providers stated that the tool did not offer any new information [111].

Of the 51 PA tools, positive perceptions regarding all RQ2 outcomes (i.e., satisfaction, content, efficiency, navigation, usability, understandability, visibility, and workflow) were reported for 30 tools. For example, physicians and adults accessing care reported that the EMR-based Electronic Case-finding and Help Assessment (eCHAT) tool was acceptable, easy to use and understand [66], and facilitated discussions of PA that otherwise may not have been initiated [67]. However, negative perceptions covering all RQ2 outcomes except satisfaction (i.e., only positive or mixed satisfaction was reported) were given for 22 tools. For instance, the 5A’s Team Tools offer a toolkit of provider counselling and prescription tools and shared decision-making resources in both paper and electronic formats, but these were perceived as “too long” by providers [112] and certain elements (i.e., mnemonics) were not seen as useful for communicating with adults accessing care [87]. Finally, there were mixed perceptions covering all RQ2 outcomes across 19 tools. One example is the Computer-Based Counselling System, an electronic tool, where some adults accessing care felt that the informational videos were relatable to them, but others felt they were not applicable to adults who are employed [113].

Positive perceptions of satisfaction were given for the one sleep tool. Most adults accessing care responded positively about the paper-based Sleep Health Materials tool and stated they would recommend it to a friend [14].

### Tool effectiveness

Among the PA/sedentary behaviour tools, It’s LiFe! was positively associated with nurses’ knowledge and ability [110] and increases in adults’ PA behaviour [110, 114, 115] while the Electronic Medical Prescription in Portugal was positively associated with frequency of PA/sedentary behaviour discussions [96]. Alternatively, the EMR PA Tool was negatively associated with physicians’ knowledge [109], and the Paper-Based Decision Tool was associated with decreases in sedentary behaviour but also PA [76]. Moreover, three tools showed mixed associations with knowledge [110] and frequency of counselling and prescription [110, 115, 116]. For example, the Rapid Assessment Disuse Index (RADI) was used to provide general advice to reduce sedentary time in 10% of adults accessing care; however, no adults received a written plan to reduce sedentary behaviour and only 2% were given specific strategies to target sedentary behaviour change [116]. One PA/sleep tool, the IWT, was not associated with any changes in physicians’ ability to provide counselling [111].

Thirty-eight of the 51 PA tools were positively associated with all RQ3 outcomes over time: increases in providers’ knowledge (*n* = 5), ability (*n* = 4), confidence (*n* = 5), and frequency (*n* = 8) of PA assessment, counselling, prescription, and/or referral, and PA behaviour (*n* = 16) among adults accessing care. For example, Activity Counselling Trial, a 3–4 min paper-based tool, was reported to improve providers’ perceived ability to counsel on PA [68] and significantly increase self-reported PA in adults accessing care compared to baseline [69]. Effect sizes of PA tools that were successful at increasing PA were only reported in two studies, with one showing large effect sizes [117] and the other showing small effect sizes [61]. Alternatively, eight tools showed negative associations across all 5 RQ3 outcomes except PA behaviour. For instance, in reference to using the PA Screen in EMR tool, providers mentioned low knowledge surrounding PA guidelines or exercise programs [28]. Five tools were not associated with any changes in confidence and frequency in PA discussions or PA behaviour. For example, the paper-based and EMR-integrated Physical Activity Vital Sign was not associated with changes in providers’ confidence to give PA advice and prescriptions from pre- to post-intervention; however, levels of confidence for giving PA advice were already high at baseline (89–94% [104]).

The one sleep tool (Sleep Health Materials tool) was associated with increases in adults’ sleep knowledge and confidence in managing sleep problems and showed positive associations with pharmacists’ sleep knowledge and frequency of counselling [14]. Effectiveness outcomes that were reported in included studies but not listed in RQ3 are included in Additional File 6.

### Evidence-based recommendations for future tool development

Recommendations and their supporting studies are presented in Table 1.

### Quality assessment

One-hundred-sixteen studies evaluated the effectiveness of tools. Of the 21 qualitative studies, quality ranged from 0–5 out of a possible 5. The most common reasons for lower scores were findings not derived from data (*n* = 2 [70, 118]), interpretation not substantiated by data (*n* = 2 [70, 118]), and lack of coherence between data sources, collection, and analysis (*n* = 2 [70, 118]). Of the 35 RCTs, study quality ranged from 0–5. Lower scores resulted from incomplete outcome data (*n* = 22 [57, 58, 68, 69, 71, 72, 78, 85, 88, 89, 97, 117, 119–128]), outcome assessors not blinded (*n* = 24 [58–60, 68, 69, 71, 72, 75, 78, 88, 97, 98, 119, 124–134]), and lack of adherence to the intervention (*n* = 18 [58–60, 68, 79, 80, 85, 88, 89, 114, 117, 120–123, 128, 129, 133]).

Of the 15 non-randomized designs, study quality varied from 0–5. Common reasons for lower scores included incomplete outcome data (*n* = 13 [54, 61, 62, 69, 73, 81, 90, 99, 135–139]) and no accounting of confounders (*n* = 14 [54, 61, 62, 73, 81, 90, 99, 100, 135–140]).

Of the 34 descriptive studies, quality ranged from 0–5. Lower scores were primarily due to sample not representative of the target population (*n* = 11 [55, 63, 66, 113, 139, 141–146]) and high risk of non-response bias (*n* = 24 [55, 63, 64, 66, 69, 74, 91, 101, 113, 139, 142–155]).

Of the 11 mixed methods studies, quality varied from 2–5. Lower scores were due to inconsistency between quantitative and qualitative results (*n* = 6 [61, 104, 110, 115, 156, 157]) and quantitative and qualitative components not adhering to the quality criteria of their method (*n* = 5 [84, 115, 156–158]). The full MMAT ratings are presented in Additional File 7.

## Discussion

This scoping review aimed to comprehensively report and appraise tools for PA, sedentary behaviour, and/or sleep discussions between health care providers and adults (18 + years) accessing care in clinical settings in Canada and analogous countries. Findings indicated that a vast number of tools to guide discussions on PA, sedentary behaviour, and sleep have been developed and implemented to varying degrees in clinical settings. The 61 tools showcased a broader picture of PA discussion tools than did previous reviews in this area [29–31] including several sedentary behaviour and sleep discussion tools that had not been previously synthesized.

Providers and adults accessing care held positive perceptions toward many (58.8%) of the included PA tools and nearly three-quarters (74.5%) were positively associated with effectiveness outcomes. These results are promising as the positively rated tools in this review can provide insight as to what specific features (e.g., length of the tool, supportive language) can be borrowed from in emerging tools or carried forward in future iterations of existing tools. Conversely, insight from studies reporting negative or mixed perceptions and effectiveness outcomes of PA tools can also be gleaned to make strategic improvements to the design and functionality of tools moving forward. For instance, many negative perceptions of included PA tools pertained to their efficiency and workflow [90, 152, 159]. It is no surprise that providers face unrelenting time constraints and workload [160], but these findings highlight the need to develop movement behaviour discussion tools that can be embedded seamlessly into a clinical encounter. Additionally, negative associations with providers’ knowledge, confidence, ability, and frequency were present for some PA tools. A systematic review found that the greatest barriers to PA counselling reported by physicians were a lack of knowledge, training, and resources [23]. However, only one tool in the present review was associated with decreases in PA (specifically MVPA) among adults accessing care [76]. This suggests that while providers may not feel knowledgeable, confident, or skilled in engaging in PA discussions, the mere act of facilitating PA discussions through a tool appears to positively influence the PA levels of adults accessing their care [114, 119, 127].

Only four tools did not cover PA assessment. That most tools included an assessment of PA in addition to counselling, prescription, and/or referral is consistent with the sequence of actions that providers enact to promote PA, as reported in the literature [19, 20]. Assessing PA is the first step to providing effective discussions of PA behaviour change and this was evidenced by many tools where data from the PA assessment step was used to prompt and support PA counselling and/or prescription later in the consultation (e.g., [70, 96, 161]).

Only one tool focused solely on sleep for health promotion purposes. Unsurprisingly, it was more detailed than the two combined PA and sleep tools, including information on sleep duration and on sleep environment and lifestyle considerations, such as sleep quality and sleep hygiene [14]. Notably, during screening, many tools were excluded given their focus on sleep disorders. The absence of sleep disorders is not always indicative of healthy sleep patterns; healthy sleep is comprised of sufficient duration and quality, suitable timing, *and* a lack of sleep disorders [162]. In contrast to the predominant focus in clinical practice on diagnosing and treating disordered sleep [163], discussions on healthy sleep behaviours should be approached whether or not an individual presents with a sleep disorder. This evidence suggests there is a need to develop more tools for assessment, counselling, prescription and referral for sleep behaviour change and health promotion in clinical settings.

Given the growing body of literature noting the differences between sedentary behaviour and inactivity [164, 165], tools focusing on increasing PA and decreasing sedentary behaviour have begun to emerge. Of the seven PA/sedentary behaviour, six were published in the last 10 years [26, 76, 96, 109, 114, 116]. Some perceptions and outcomes of the PA/sedentary behaviour tools were mixed or negative; however, three tools were positively related to enhancements in providers’ knowledge, ability [110], and frequency discussing movement behaviours [96], and adults’ sedentary behaviour [76]. The Paper-Based Decision Tool [76] was also associated with small reductions in MVPA. It is unclear what may have caused this drop in PA, though perhaps, in an effort to conserve their energy to reduce their sedentary behaviour, participants in the study may have simultaneously traded some MVPA for lighter-intensity PA. Given this study was published before the 24HMG for Adults were released [1], providers were unlikely counselling participants on the integrated relationships between PA, sedentary behaviour, and sleep. Therefore, future tools combining movement behaviour recommendations should promote trading sedentary time for light PA without decreasing MVPA (or with increasing MVPA where possible), while preserving sufficient sleep. Further, as a low number of sedentary behaviour tools have been evaluated, more research is needed to establish best practices for developing tools that target healthy sedentary behaviour habits overall, and in the context of a 24-h movement paradigm.

There are several explanations for these mixed results. One could be that sedentary behaviour remains an unfamiliar topic for some providers and adults accessing care, leading to fewer instances where sedentary behaviour discussions are initiated in clinical settings. For instance, breaking up sedentary time is known to have health benefits [1]; however, the 24HMG lack suggestions on how often sedentary time should be interrupted (e.g., every 30 min, every 60 min, etc.), therefore providers may feel unsure what practical recommendations to make. Indeed, providers have reported limited knowledge about sedentary behaviour counselling and a desire for education on the topic [24]. Another reason is that sedentary behaviour can be difficult to quantify in absence of objective measures (e.g., accelerometers), thus adults may misperceive how much sedentary time they are engaging in. Low awareness of sedentary behaviour guidelines in the general population has also been described [166]. Two recent reviews [167, 168] have emphasized that establishing knowledge, awareness, and positive attitudes should precede efforts to improve self-efficacy, intentions, and actual performance of a given behaviour. Thus, it is logical that the included sedentary behaviour tools were associated with varying degrees of confidence, ability, and frequency of use.

User perceptions and effectiveness outcomes were only reported for four of the nine multi-behaviour tools (3 PA/sedentary behaviour tools: EMR PA Tool, It’s LiFe!, Paper-based Decision Tool; 1 PA/sleep tool: IWT). Similar to the sleep-only tool, the studies reporting on PA/sleep tools did not evaluate sleep behaviour. Further, studies for only two of the PA/sedentary behaviour tools (RADI; Paper-based Decision Tool) evaluated for changes in sedentary behaviour. Thus, gaps exist on whether discussions on PA and sleep were more successful than discussions on PA/sedentary behaviour tools or what features of multi-behaviour tools may make them successful or not. In comparison, user perceptions and changes in PA were assessed in many of the PA-only tools, which allowed us to synthesize what features may have influenced their success (e.g., theoretical basis, low time to administer) into our recommendations for future movement behaviour tools in Table 1. Future studies implementing sedentary behaviour and sleep health promotion tools should strive to measure and evaluate changes in sedentary time (i.e., occupational and recreational screen time, non-screen-based sedentary time [169]) and sleep behaviour (i.e., duration, quality, and timing [162]) in adults accessing care to ascertain why sedentary behaviour and sleep tools may or may not influence behaviour change. Finally, tool implementation should also involve a process evaluation to gauge whether tools are being used as intended.

### Practice implications

All tools but one [14] focused on the assessment, counselling, prescription and/or referral of PA, which was in line with our expectation that the vast majority of movement behaviour discussion tools would target only PA. Given that the 24HMG [1] were relatively new at the time our searches were run, and that we included studies published since 2000, it is understandable that the integration of PA, sedentary behaviour, and sleep was not reflected in any tool. Unexpectedly, we discovered nine tools that integrated two of the three movement behaviours (see Additional Files 3 and 4). Targeting the integration of PA, sedentary behaviour, and sleep in clinical discussions can present opportunities to broach health promotion discussions for multiple movement behaviours concurrently, augmenting the potential impact of a single discussion. Discussing one movement behaviour can open the door to conversations about how changes in one behaviour can influence changes in the other two behaviours within a 24-h day [2, 3]. For instance, adults can be advised to replace high sedentary time with more PA or more sleep, and both avenues will help the individual achieve more favourable health outcomes [1]. However, a person-centered approach that respects adults’ individual needs and preferences should be used, as movement behaviour discussions will likely be different for people of different ability levels, with or without chronic conditions [170]. Moving forward, researchers could investigate how shared decision making in discussions about PA, sedentary behaviour, and sleep in clinical settings influences the health outcomes of adults accessing care of all abilities and health statuses.

Several tools were accompanied by training and resources [14, 82, 104]. In one study, 45% of adults accessing care found that the take-home printouts for the Patient-centered Assessment and Counselling for Exercise (PACE +) tool helped them change their PA behaviour [82]. Another study found that providers who received a single training session on the PAVS tool were significantly more likely to assess, counsel, and prescribe PA compared to providers who did not receive training [104]. Accompanying training resources, such as user guides or workshops that target knowledge, confidence, and skills may help users digest the content of tools and improve their effectiveness and feasibility in practice. When translating movement behaviour tools into primary care, available PA, sedentary behaviour, and sleep interventions (e.g., community-based programs, workplace interventions, movement/sleep studies) are other resources to consider. While challenging, establishing clear referral pathways to support adults in achieving healthy movement behaviours beyond the primary care discussion will be necessary.

A small number of tools included device-based measures of movement behaviours, such as apps, accelerometers, or pedometers. Increasingly, devices have made movement behaviour data readily available, which may increase adults’ awareness of movement behaviour deficits and interest in interventions to improve them. Ideally, appropriate data management systems should be used or developed to store, and possibly integrate, data across movement behaviours to better inform providers’ movement behaviour advice.

Based on our findings, we offer seven recommendations for future movement behaviour discussion tool development (Table 1) in an effort to close the abovementioned gaps. Following these recommendations is advisable as it may inform the development, refinement, and/or implementation of discussion tools that are well-received by their intended audiences and more effective at guiding discussions that integrate PA, sedentary behaviour, and sleep.

### Strengths and limitations

This scoping review contributes to the literature by investigating the characteristics, perceptions, and effectiveness of tools for PA, sedentary behaviour, and sleep discussions in primary care settings. The integrated knowledge translation approach [36, 37] allowed us to engage relevant knowledge partners who were invested in the review topic to increase the applicability and uptake of our findings. Moreover, we used a modified coding framework [50] to categorize our RQ2 outcomes, which we found valuable for capturing a range of perceptions about tools. Future reviews or qualitative research could similarly use this framework to structure their coding of study outcomes or transcripts, respectively. Importantly, this scoping review informed a list of recommendations that researchers and health care providers, including ourselves, can use to guide the development of evidence-based, positively-valued, and effective health promotion tools that integrate all movement behaviours.

Despite our rigorous search strategy, it is possible that some relevant studies were missed. For instance, studies published in languages other than English were excluded; however, a recent study suggested that the results of 59 Cochrane reviews did not significantly change when non-English studies were excluded [171]. Moreover, several English studies were excluded as they reported on tools published in languages other than English, which was one of our exclusion criteria. Nevertheless, this did not limit the comprehensiveness of our scoping review as 135 studies spanning 61 tools were retrieved and synthesized, which is greater in scope than previous reviews [29–31]. Finally, our eligible health care provider populations did not include qualified exercise professionals (QEPs; e.g., Kinesiologists), who are another ideal group to promote movement behaviours as it is relevant to their scope of practice and they have the requisite education and training [172]. Future research is warranted to review movement behaviour discussion tools that are used, or intended for use, among QEPs to ascertain whether tool characteristics or the effectiveness of movement behaviour discussions differ in this context compared to clinical settings. In some settings, QEPs may already be integrated within the health care team [173].

## Conclusions

Clinical discussion tools have the potential to enhance the promotion of movement behaviours between providers and individuals who access care. In this scoping review, we identified a large number of studies, with 51 focusing on PA, one focusing on sleep, and nine combining two movement behaviours. Many tools were positively perceived and effective at enhancing knowledge of, confidence for, ability in, and frequency of movement behaviour discussions, and most were used or intended for use among physicians and adults without chronic conditions aged 18–64 years. However, to fill remaining gaps in knowledge and practice, tools should be designed to guide discussions of all three movement behaviours in an integrated manner and researchers and providers should consider using our list of seven evidence-based recommendations to inform future tool development and refinement.

The following studies included in this review were not referenced in this manuscript but are referenced in Additional Files 3, 4, 5 and 6: [174–199].

## Supplementary Information

**Additional file 1: **PRISMA-ScR checklist.

**Additional file 2: **Search strategy.

**Additional file 3: **Physical activity tools (*n *= 51).

**Additional file 4: **Sleep tools (*n *= 1).

**Additional file 5: **Multi-behaviour tools (*n *= 9).

**Additional file 6: **Perceptions and effectiveness outcomes of included discussion tools.

**Additional file 7: **Mixed Methods Appraisal Tool (MMAT) Quality Assessment Ratings.

## Data Availability

The datasets used and analysed during the current study are available from the corresponding author on reasonable request.

## Abbreviations

24HMG: 24-Hour Movement Guidelines
eCHAT: Electronic Case-finding and Help Assessment tool
EMR: Electronic medical record
It’s LiFe!: LIfestyle FEedback! Tool
IWT: Integrated Wellness Tool
MMAT: Mixed Methods Appraisal Tool
MVPA: Moderate-to-vigorous physical activity
PA: Physical activity
PACE +: Patient-centered Assessment and Counselling for Exercise
PRISMA-ScR: Preferred Reporting Items for Systematic Reviews and Meta-Analyses Extension for Scoping Reviews
QEP: Qualified Exercise Professional
RADI: Rapid Assessment Disuse Index
RQ: Research question

## Footnotes

1 The 24-Hour Movement Guidelines recommends that adults ages 18–64 years preserve 7–9 h of good-quality sleep per day, limit sedentary behaviour to ≤ 8 h per day (including ≤ 3 h of recreational screen time per day), obtain ≥ 3 h of light physical activity per day, and achieve ≥ 150 min of moderate-to-vigorous physical activity per week (including ≥ 2 days of strength training). For adults 65 + years, the sleep recommendations are 7–8 h of good quality sleep per day and the physical activity recommendations also include balance training [1].
