# Supplementary material for: Tools to guide clinical discussions on physical activity, sedentary behaviour, and/or sleep for health promotion between primary care providers and adults accessing care: a scoping review
Source: BMC Prim Care. 2023 Jul 7;24:140. doi: 10.1186/s12875-023-02091-9 (PMC10326959; doi:10.1186/s12875-023-02091-9)
Supplement: Supplementary file 2 — Additional file 2: Search strategy. [file 12875_2023_2091_MOESM2_ESM.docx]

**Multimedia Appendix 2.** Search strategy.

Database: Ovid MEDLINE(R) and Epub Ahead of Print, In-Process, In-Data-Review & Other Non-Indexed Citations, Daily and Versions(R) <1946 to June 22, 2021>

Search Strategy:

--------------------------------------------------------------------------------

1 exp Exercise/ (211495)

2 exp Exercise Movement Techniques/ (8872)

3 exp Exercise Therapy/ (55014)

4 Physical Exertion/ (56858)

5 Motor Activity/ (98162)

6 exp Sports/ (193296)

7 Sedentary Behavior/ (10996)

8 Screen Time/ (594)

9 Sleep/ (56840)

10 ("Physical activit*" or "Physical inactivit*" or "Physical train*" or "Physical fitness*" or Exercise or "Bodily movement*" or "Leisure activit*" or "Walk" or "Lifestyle" or "life style" or "strength train*" or "balance exercis*" or "postural balan*" or "balance train*" or "falls" or "accidental fall*" or "stability train*" or "instability train*").ab,ti,kw. (550238)

11 (Sedentar* or "sitting" or "low energy expenditure*" or "Bed rest*" or (Computer adj3 time) or (television adj3 time) or "screen time").ab,ti,kw. (66697)

12 Sleep*.ab,ti,kw. (197610)

13 or/1-12 (1018800)

14 Decision Making, Shared/ (979)

15 decision support techniques/ (21325)

16 Patient Participation/ (27207)

17 decision support*.mp. (40905)

18 shared decision*.mp. (10579)

19 Patient Education as Topic/ (86943)

20 exp Directive Counseling/ (4480)

21 ((discussion* or conversation* or counsel* or referral*) adj2 tool*).mp. (694)

22 Patient Education Handout.pt. (5377)

23 or/14-22 (167080)

24 exp Primary Health Care/ (169936)

25 primary care*.mp. (131174)

26 family nurse practitioners/ or general practitioners/ or physicians, family/ (25096)

27 "family practice*".mp. (69243)

28 exp General Practice/ (75930)

29 "primary health care*".mp. (97700)

30 or/24-29 (336330)

31 13 and 23 and 30 (1753)

32 limit 31 to (english language and yr="2000 -Current") (1447)

33 limit 32 to "all child (0 to 18 years)" (209)

34 32 not 33 (1238)

***************************

1.

General practice nurses' communication strategies for lifestyle risk reduction: A content analysis.

James S, Mcinnes S, Halcomb E, Desborough J

Journal of Advanced Nursing. 76(11):3082-3091, 2020 Nov.

[Journal Article]

UI: 32898932

Authors Full Name

James, Sharon, Mcinnes, Susan, Halcomb, Elizabeth, Desborough, Jane
