# Supplementary material for: Tools to guide clinical discussions on physical activity, sedentary behaviour, and/or sleep for health promotion between primary care providers and adults accessing care: a scoping review
Source: BMC Prim Care. 2023 Jul 7;24:140. doi: 10.1186/s12875-023-02091-9 (PMC10326959; doi:10.1186/s12875-023-02091-9)
Supplement: Supplementary file 3 — Additional file 3: Physical activity tools (n = 51). [file 12875_2023_2091_MOESM3_ESM.docx]

**Multimedia Appendix 3.** Physical activity tools (*n* = 51)

| **Tool** | **User** | **Target Population** | **Format** | **RQ1** | | | **RQ2^e^** | **RQ3^e^** | **References** |
| --- | --- | --- | --- | --- | --- | --- | --- | --- | --- |
|  |  |  |  | **Guideline** | **TMF** | **Description^a^** |  |  |  |
| 5A’s Team Tools | Physicians  Dieticians  Psychologists/Psychotherapists/Social workers | Adults with overweight or obesity | Paper and electronic^b^ |  | 5 A’s Framework, Theoretical Domains Framework | Toolkit based on the 5 A’s Framework that incorporates the 4 M’s of obesity (mental, mechanical, metabolic, and monetary root causes, complications, and barriers to managing obesity). Includes communication tools (4 M’s assessment tool (A), decision-making tool to assess strengths and challenges per the 4 M’s, SMART goal-setting sheet, and weight loss information; C), provider tools (PA prescription (P), and messaging about the 4 M’s of obesity), tools for adults accessing care (obesity fact sheet, relapse prevention tool). | + satisfaction, content, usability, visibility, workflow  − efficiency  +/− understandability | ↑ knowledge, ability, confidence, frequency | [62,63,104,105,149] |
| 10 Simple Steps | Physicians | Adults 18-64 years | Paper | National Health and Medical Research Council Clinical Practice Guidelines for the Management of Overweight and Obesity in Adults, Adolescents, and Children in Australia (2013)^e^ |  | Prudence Score assesses PA behaviour (A), the results of which are used to inform counselling materials. Counselling materials include a 1-page personalized computer-tailored feedback that states which behaviours (incl. PA) are below guideline recommendations and a 1-page health promotion information sheet with tips for improving behaviours, information on health benefits, and links to more information (C). The counselling materials can be viewed independently by adults accessing independently or in collaboration with a physician. | + satisfaction  − efficiency  +/− workflow | + knowledge  Ø PA behaviour *(adults accessing care)*  +/− abiltiy  + frequency | [101,159] |
| 10 Top Tips | Physicians | Adults with overweight or obesity | Paper | UK Chief Medical Officer’s Physical Activity Guidelines (2019) and US Department of Health and Human Services Physical Activity Guidelines (2008) |  | A 2-3 minute intervention to be used by providers in primary care visits to discuss lifestyle, incl. PA (C). Two of the 10 tips relate to PA recommendations: (1) “Walk off the weight” and (2) “Up on your feet” | + content, usability | + PA behaviour^*^ | [54] |
| 10,000 Steps Counselling Materials | Physicians | Adults 18-64 years | Paper and pedometer^b^ |  |  | Adults are provided with pedometers to assess and monitor PA (A). Physicians receive training in counselling techniques (e.g., motivational interviewing) and are provided materials, incl.: PA brochures, posters, and PA counselling tip sheets focused on “Ask, Advise, Assist, and Arrange” components (C). | + usability | ↑ knowledge^*^  Ø frequency | [137] |
| ACT (Activity Counselling Trial) | Physicians | Adults 18-64 years | Paper | Centers for Disease Control and Prevention and American College of Sports Medicine PA Recommendations | Social Cognitive Theory, Trans-theoretical Model | 3-4min protocol to assess (A), advise (C), and refer (R) using a standardized “Current Physical Activity form” | + satisfaction, efficiency, workflow | ↑ ability, PA behaviour^*^  + frequency | [69,70,88,89] |
| ADAPT (Avoiding Diabetes Thru Action Plan Targeting) | Physicians | Adults with chronic conditions | EMR |  | Social Cognitive Theory, Trans-theoretical Model, Health Belief Model | Pedometer use for 1 week (A), EHR-embedded counselling tool with a focus on feedback to increase self-efficacy plus web-based brief action plans using “if-then” statements (C), SMART goals tracking flowsheet and signed behaviour change prescription (P) integrated into instructions for adults accessing care, and persuasive elements (social comparisons via website, behaviour change material given by provider, testimonials). Weekly emails, and biweekly report (F). | + satisfaction, content  ↑ efficiency  − navigation, usability, understandability, workflow | ↑ PA behaviour^*^ | [73,87,175,176] |
| ARCH (Activating Resources for Community Health Promotion) | Physicians | Adults 18-64 years | Paper and electronic^b^ |  |  | Education and self-management resources in 5 areas: (1) health information (2) informed decision making (3) peer information exchange and emotional support (4) tools and calculators to assess risk, (5) promoting self-care and self-efficacy. The Prescription for Health Pad facilitates discussion of health behaviors, goal-setting, planning, and follow-up and includes a checklist of website resources (C, P). A searchable database of community programs for smoking cessation, PA, diet/nutrition, and weight management facilitates referral to community programs (R). | − workflow | − ability  ↑ frequency^*^ | [143] |
| BETTER 2 [and BETTER WISE] Programs (Building on Existing Tools to Improve [Cancer and] Chronic Disease Prevention and Screening in Primary Care [for Wellness of Cancer Survivors and Patients]) | Physicians | Adults with chronic conditions | Paper |  | Motivational interviewing | BETTER 2 prevention and screening toolkit includes: patient health surveys that assess weekly/occupational/readiness for/confidence for PA (A), prevention visit form, bubble diagrams that assist providers in educating on disease risk factors (C), prevention prescription with goal-setting (P) and referral component (R), and a care map to guide decision-making. Use of all components may take up to 1 hour.  BETTER WISE toolkit includes: blended care pathways, poverty screening tool, and the “Patient Health Survey”, bubble diagrams, and prevention prescriptions (from BETTER 2), targeted at cancer surveillance. | + satisfaction, +/− content *(adults accessing care)*  +/− satisfaction, usability,  + content *(physicians)*  − efficiency | + knowledge, frequency  ↑ PA behaviour | [132,145,154,177,178] |
| BFWHW (Bright Futures for Women’s Health and Wellness Initiative) | Physicians | Adults 18-64 years | Paper | U.S. Department of Health and Human Services Physical Activity Guidelines for Americans (2008) | Theory of Planned Behaviour | Includes a “Counselling Support Tool” that asks 5 questions on aerobic and strength activities [i.e., How many days/week of PA; How much time spent doing PA on a typical day; How much moderate intensity PA/day; How much vigorous intensity PA/day; Which types of strength training activities engaged in an how many times/week (A)]. The tool encourages discussions between provider and individual accessing care to promote self-management, goal-setting, and behaviour change (C). | + satisfaction, usability  − content, efficiency | + confidence  ↑ frequency | [115] |
| CBCS (Computer-Based Counselling System) | Physicians | Adults with chronic conditions | Electronic |  | Trans-theoretical Model, Social Cognitive Theory | Computer-based tool completed in the waiting room. Tool includes a brief PA behaviour assessment that asks whether the individual has performed moderate PA for ≥ 30min 5x/week or if they intend to do so (A) and structured PA information that is tailored to the individual’s stage of change, incl.: discussions of barriers and facilitators, PA benefits, encouragement, and goal-setting (C). One consultation takes about 10min. | +/− content  + understandability, usability, visibility  − workflow | ↑ confidence *(adults accessing care)* | [64] |
| CD-ROM Tool for Managing Obesity in Primary Care | Nurses | Adults with overweight or obesity | Electronic |  | 5 A’s Framework | Structured discussions guided by a computer program, covering assessment of anthropometrics (A), followed by personalized goal-setting, keeping a diary, advice for coping with setbacks, and discussion of the benefits of increased PA, monitoring PA habits, and setting fortnightly lifestyle goals. Delivered alongside weight-management training for providers and the provision of pedometers (C). | +/− satisfaction | + frequency *(adults accessing care)* | [102] |
| CDSS (Clinical Decision Support System) for Physical Activity and Diet | Physicians | Adults who smoke | Electronic | Canadian Society for Exercise Physiology Physical Activity Guidelines for Adults (2011) | Motivational Interviewing | The CDSS for PA and Diet is integrated into an online portal for smoking cessation treatment (STOP portal) and allows providers to: screen for PA levels (A), be alerted when an individual does not meet the national PA guidelines (A), guide providers to discuss PA as a risk factor for smoking cessation with the individual seeking care using a script (C), and share self-monitoring resources. | N/A | N/A | [161] |
| The Change Program | Physicians  Dieticians  Psychologists | Adults with overweight or obesity | Paper |  |  | Workbook incl.: goal-setting, information about PA and behavioural change supports, and a PA diary (A). Handbook for providers with discussions of challenges in weight-loss interventions (incl. PA) and troubleshooting (C). | +/− content, workflow  − navigation  + usability | N/A | [179] |
| Computer-based Lifestyle Intervention Concept | Adults accessing care | Adults 18-64 years | Electronic | CDC and ACSM Recommendations for Physical Activity and Public Health (1995) |  | Computer-based "test" that assesses PA behaviour, motivation to change, and generates tailored advice. Questions measures days/week of moderate PA of ≥30min, days/week of vigorous PA, and intention to change behaviour in the next 6 months (A). Scores are categorized as “physically active" or "insufficiently active". Based on responses, the program generates tailored advice that can be discussed further with the provider (C). | + satisfaction, usability, content  − workflow | − frequency, knowledge  ↑ ability  + confidence | [142,174,180] |
| Computer-tailored Health Communication Program | Adults accessing care  Physicians | Adults 18-64 years | Electronic |  |  | Program that collects data and gives feedback based on: PA level, readiness to change, decisional balance, processes of change, and self-efficacy. Questions assess risk factors and frequency of symptoms related to PA (e.g., fatigue) (A). Feedback explains how becoming physically active would improve risk factors. A feedback report is created for physicians to prompt and guide them in counselling to adopt or maintain PA (C). Requires an average of 10min to complete. | − efficiency, understandability, usability, workflow | − frequency | [72] |
| CONNECT (Consumer Navigation of Electronic Cardiovascular Tools) | Adults accessing care | Adults with chronic conditions | EMR |  |  | Interactive, web-based application linked to EMR, pre-populated with CVD risk and incl. PA assessment (A). Also includes goal-setting, reminders and motivational messages, reward system, social comparison strategies, and education (C). | + content, navigation, understandability, usability, visibility, workflow | ↑ knowledge^*^, PA behaviour^*^ | [100,150] |
| CTH (Connection to Health) | Physicians  Nurses | Adults with chronic conditions | Electronic |  |  | Program helps identify: (1) self-reported management problems, (2) self-reported management priorities, and (3) an action plan and follow-up with a provider to select a self-management goal. Web-based assessment of current self-management over 12 areas (incl. PA) is conducted in face-to-face meetings the provider (A). Automated algorithms flag problem areas using colours (green = low need for change; yellow = some need for change; red = high need for change) and recommend areas for change. A summary is shown, after which adults are prompted to prioritize up to 2 areas to discuss with the provider; a meeting is held to review the assessment results, select a goal, and develop an action plan to achieve the goal (C). | − workflow | − knowledge | [133] |
| EASY (Exercise Assessment and Screening for You) | Adults accessing care  Physicians | Adults 65+ years | Electronic |  |  | Web-based tool that matches underlying health problems with a PA program that is safe for said health problems. Includes an interactive, web-based system to guide providers and older adults through 6 questions. The questions aim to identify health problems that could affect the type of PA the adult may perform and highlight activities that may best benefit their health problems. An algorithm guides adults to a list of appropriate PA programs and prompts adults to set a goal to walk 30min/day most days/week and review safety tips to follow before, during, and after PA (P). | N/A | + PA behaviour^*^ | [134,199] |
| eCHAT (Electronic Case-finding and Help Assessment Tool) | Adults accessing care  Physicians  Nurses | Adults 18-64 years | EMR |  | Self-efficacy Theory | Tool is self-administered by adults on an iPad in the waiting room or remotely and takes 2-5min; the provider receives an electronic summary report at the point of care with scored and interpreted results and decision support tools. Assesses for unhealthy behaviours, incl. physical inactivity (A), facilitates a conversation about aspects of adults’ lives they consider important and wish to change, decision making, and self-management (C), and offers possible interventions using a stepped care model. Stepped care includes: (1) self-management resources (e.g., written information), (2) provider resources (e.g., motivational interviewing), (3) community-based referrals, and (4) referrals to secondary services (R). Includes an innovative Help question that asks adults whether they would like help with specific issues, during their visit or later, or want to take any action to improve their mood or health behaviors. | + satisfaction, understandability, workflow  +/− content, efficiency, usability  − visibility | + frequency, ↑ confidence *(adults accessing care)* | [60,61] |
| eHealth Tool | Physicians  Adults accessing care | Adults 18-64 years and adults with chronic conditions | EMR and electronic^b^ | Canadian Society for Exercise Physiology Physical Activity Guidelines for Adults (2011) | Health Action Process Approach | Electronic survey via email or tablet to assess perceived barriers and motivators for, and levels of, PA (A). Data are summarized in EMR and compared to PA guideline benchmarks and are used to create tailored resources and a customized PA prescription based on adults’ PA levels and motivators in the EMR (P). The EMR is also populated with links to PA toolkits of community-based resources tailored to the adults’ PA levels and barriers, or a condition-specific PA toolkit if the adult reported a chronic condition (R). The prescription can be edited by the provider in discussion with the adult and printed alongside the toolkit to take home. | + satisfaction, efficiency | +/− frequency  ↑ PA behaviour | [94] |
| EMPOWER-H (Engaging and Motivating Patients Online With Enhanced Resources - Hypertension) | Nurses  Dieticians  Pharmacists | Adults with chronic conditions | EMR, mobile, and pedometer^b^ |  | Theory of Planned Behaviour, Health Belief Model, Social CognitiveTheory | Interactive, web-based disease management system integrated in EMR. Inclu: (1) wireless BP monitor that uploads in the EMR and EMPOWER system, (2) smartphone with 2 apps, (3) comprehensive dashboard of the status of personalized action plans, treatment goals, and self-monitoring data, available from within a web-based portal (“My Health Online”), (4) pedometer for monitoring steps, (5) web-based messaging system for communicating between providers and adults accessing care, (6) Nurse Care Managers assisted by Registered Dieticians for nutrition and weight management and consultation with pharmacists (related to medication), and (7) text and video educational nuggets (e.g., motivational text on step goals) sent by Nurse Care Managers (C). Adults are encouraged to use their pedometer to upload daily step count (A). | N/A | ↑ PA behaviour^*^ | [114] |
| EVS (Exercise Vital Sign) | Nurses | Adults with chronic conditions | EMR | U.S. Department of Health and Human Services Physical Activity Guidelines for Americans (2008) |  | Tool embedded in “vital sign” section of EMR for identifying adults who are not meeting the PA guidelines and assisting providers in promoting PA. Includes 2 questions that are asked and entered into the EMR on days/week and how many minutes of moderate to strenuous PA (A). Response choices for days are categorical (0–7). Minutes are recorded in blocks of 10 (0, 10, 20, 30, 40, 50, 60, 90, 120, and ≥150). EMR software multiplies the two self-reported responses to display minutes per week of moderate or strenuous PA for the provider to review and use to initiate brief counselling sessions with adults accessing care (C). Comment section is provided to note any issues with the assessment. Takes < 1min to administer. | + satisfaction, usability | ↑ frequency^*^ | [109,139,140,181] |
| Exercise Prescription Model | Physicians | Adults 18-64 years | Paper |  |  | Structured approach per each first and follow-up visits. First visit: rapport building, clinical history and lifestyle assessment (A), explanation of benefits of lifestyle change (incl. discussion of motivation to change), goal-setting (i.e., reasons for change, prioritize a behaviour, define short- and long-term goals), education about PA and reducing SB (i.e., practical strategies, desire to adhere) (C). Goal-setting and education are used to create a prescription (P). Follow-up visit: rapport building, clinical history and lifestyle assessment (A), analyzing results, barriers encountered, and progression of goals, discussing benefits experienced, problem- solving (solutions to overcome barriers, share resources), and additional goal-setting. | N/A | N/A | [182] |
| Food and Physical Activity Habit Inventory | Physicians | Adults 18-64 years | Paper |  |  | 2-sided form containing a checklist of questions (incl. on intensity and frequency of PA and sedentary activities, such as computer use and television viewing) (A). A second 2-sided tip sheet was designed to help physicians use the form. In addition to discussing their PA and SB, adults accessing care are asked to set a realistic goal to discuss with the physician (C). | N/A | ↑ PA behaviour^*^ | [117] |
| GDRS (German Diabetes Risk Score) | Physicians | Adults with chronic conditions | Paper |  |  | Tool focuses on modifiable non-invasive risk factors and consists of 11 questions on anthropometrics, PA, other lifestyle factors (i.e., smoking, nutrition, and family history of diabetes to predict 5-year diabetes risk (A). A visual presentation of the adult’s diabetes risk is used during counselling with the physician to discuss tailored preventive strategies and short recommendations to enhance healthy lifestyle (C). | N/A | N/A | [183] |
| GEM (Goals for Eating and Moving) | Adults accessing care  Nurses | Veterans | Electronic |  | 5 A’s Framework, Theory of Planned Behaviour | Self-administered by adults on an iPad in-clinic. The Health Coach (nurse) is the main facilitator of tool, lightening the burden for counselling sessions with providers, which should take <5min. Includes 16 questions on lifestyle behaviours and barriers (A) followed by tailored weight loss and behavior change advice. Adults are guided to rate their perceived importance of each piece of advice and a ranked list of potential goals is generated based on the advice and importance ratings. Adults are to choose goals (incl. a PA goal), then receive personalized tailored materials as assembled by the Health Coach (C). | +/− satisfaction, content  − efficiency, usability, workflow | Ø PA behaviour | [86,184] |
| GOALS (Goal-focused Online Access to Lifestyle Support) | Physicians | Adults with chronic conditions | EMR |  |  | Online adaptation of the Diabetes Prevention Program that incorporates online education and coaching. Includes PA tracking (A), diet reporting, and educational modules with personalized lifestyle coaching (C) | + content  +/− understandability, usability, workflow  − navigation, visibility | N/A | [151] |
| General Practitioner Physical Activity Project | Physicians | Adults 18-64 years | Paper | National Physical Activity Guidelines for Australians (2002) | Health Belief Model, Trans-theoretical Model | Flow-chart of 4 steps: (1) identifying target group and when to intervene, (2) assessing PA level and decision to discuss further (A), (3) advising, suggesting, informing, referring, encouraging, or reinforcing behaviour (C, R), and (4) system reminders that the physician has screened and discussed PA with the adult. If adults report insufficient PA, physicians give verbal advice and written information; if adults report sufficient PA, physicians encourages and reinforces the behaviour. Flow-chart was accompanied by PA assessment questionnaires, background information on PA counselling, a directory of PA options suitable for beginners, an education handout, and recall and reminder stickers. | N/A | ↑ PA behaviour^*^ | [108] |
| GPPAQ (General Practitioner Physical Activity Questionnaire) | Physicians | Adults 18-64 years | Paper | National Institute for Clinical Excellence Physical Activity Guidelines for Adults (2013) |  | Short, self-report questionnaire on PA and occupation and is scored into “active”, “moderately active”, “moderately inactive” or “inactive” categories (A). “Active” is consistent with achieving PA guidelines relating to time spent in MVPA or VPA; all other categories require a PA intervention. | + efficiency  − usability | + frequency | [113,129] |
| Green Prescription/Enhanced Green Prescription | Physicians  Nurses | Adults 18-64 and 65+ years and adults with chronic conditions | Paper^c^  Pedometer^d^ | New Zealand Eating and Activity Guidelines | Motivational Interviewing, Trans-theoretical Model | Green Prescription includes assessing adults’ PA levels to determine whether they are “inactive” (A), brief counselling (about 7-13min) using motivational interviewing techniques and individualized to the adult's age, capability, medical condition, and every day activities (C), specific and tailored advice written on a “green prescription” and given to the adult, usually recommending home-based PA and walking tasks (P), and faxed green prescriptions to exercise specialists at the Regional Sports Foundation (R). Adults are encouraged to use a pedometer to monitor their steps.  Enhanced Green Prescription includes additional telephone support through referral to a community-based exercise facilitator. | +/− satisfaction, usability, workflow  − efficiency | − knowledge  +/− confidence  + frequency  ↑ PA behaviour^*^ | [81-85,120,121,124,127,128,146,152,185] |
| HausMed Website | Physicians | Adults with overweight or obesity | Electronic |  |  | After a pre-assessment on PA in a structured form (A), the program tailors coaching based on physician recommendations and the adult’s physical characteristics and everyday behaviour (C). The adult receives a copy of the pre-assessment form, which advises to use the coaching program. The coaching program uses individualized education, motivation, exercise guidance, daily text message reminding, weekly feedback and active monitoring (incl. ~3 phone calls over 12 weeks by physicians or their staff), and referral from the website to applicable programs (R). Available at https://www.hausmed.de/ (translatable to English) | N/A | ↑ PA behaviour^*^ | [80] |
| I-ACE (Interactive Lifestyle Assessment, Counselling, and Education Software) | Dieticians | Adults with chronic conditions | Electronic | World Health Organization Physical Activity Recommendations for Adults (2010) | Motivational Interviewing, Behaviour Change Techniques | Software for tracking tailored healthy lifestyle programs using food, nutrient, and PA databases and evidence-based age-specific, sex-specific, or health status-specific goal packages. Lifestyle (dietary intake, leisure PA) is quantitatively assessed (A) to identify lifestyle behaviours as targets for education and behaviour change. Counselling then occurs using motivational interviewing and behaviour change techniques (simulation, goal setting, goal modification) to document the adult’s willingness to change and identify the minimal amount of change needed for maximal impact. Agreed-upon changes are summarized in a take-home report and followed up in subsequent counselling sessions (C). The tool uses dietary and PA data to calculate actionable, graphically displayed summary measures (e.g., average daily or weekly PA). | + satisfaction, usability, visibility  − content  +/− understandability | + PA behaviour | [79] |
| Life’s Simple 7 and My Life Check | Physicians  Nurse Practitioners | Adults with overweight or obesity and/or chronic conditions  Adults 65+ years | Paper and EMR^b^ |  | Social Cognitive Theory, Motivational Interviewing | Life’s Simple 7: Automated cardiovascular health metric embedded in EHR and accessible to adults via online portal. Calculation classifies 7 modifiable factors into *ideal, intermediate,* or *poor* categories: smoking status, body mass index, healthy diet, total cholesterol, blood pressure, fasting plasma glucose and mins/week of MVPA (A). System generates graphics of the adult’s current metric, and how it may change if adjustments to modifiable factors are made. The tool alerts providers to missing data, which requires their manual input, and higher risk adults, to prompt discussions on modifiable risk factors (C) and develop individualized action plans (P). Providers also receive system training, a summary document, and tip sheets. A resource to aid lifestyle change incl: report summaries, educational information, self-monitoring tools (e.g., PA tracking sheets), and goal-setting worksheets.  My Life Check: scores overall cardiovascular health from 0-10 based on 4 behavioural factors (maintaining a healthy weight, eating patterns, PA, and smoking) and 3 biomarker levels (blood pressure, blood cholesterol, blood glucose). Computer-based tool with an algorithm that calculates a weighted score and generates an action plan.  Accessible via http://mylifecheck.heart.org | + usability, visibility | + ability *(nurse practitioners),*  confidence *(adults accessing care)* | [125,135,187] |
| MIS (Minimal Intervention Strategy) | Physicians | Adults 18-64 years and adults with overweight or obesity | Paper |  | Trans-theoretical Model, Goal-setting Theory | Consists of two flow charts to assist with healthy nutrition and PA behaviour change. The screening flow chart helps determine if the adult is motivated for weight management guidance related to nutrition and PA or not (A). The treatment flow chart guides the physician or nurse to guide through basic nutrition and PA counselling for weight management (C). The treatment is supported by a manual explaining how to use the flow charts, a desktop flipchart, and educational materials for adults incl. fillable forms on motivation and expectations. | + content, efficiency  +/− understandability | + knowledge, ability  − confidence | [188] |
| MyPlan | Physicians | Adults 18-64 years | Electronic | ACSM and American Heart Association Physical Activity Recommendations (2007) | Self-Regulation Theory, Health Action Process Approach | Dynamic delivery to allow for several assessment and feedback moments over multiple sessions. Information modules are accessible online prior to the visit. Other components are completed by adults on a tablet in the waiting room or during their visit. Includes: (1) questionnaire about a chosen behaviour (A); (2) computer-tailored feedback based on questionnaire responses; (3) action planning using questions on barriers and facilitators to behaviour change and goal-setting (P); (4) feedback from providers (C); and (5) follow up. Adults may return to the website to re-assess their behaviour, evaluate goal progress, and receive further tailored feedback. | + satisfaction  − navigation  +/− content, efficiency, understandability, usability, visibility, workflow | + knowledge, frequency  +/− ability  ↑ PA behaviour^*^ | [74,189,190] |
| PA Screen in EMR | Physicians | Adults 18-64 years | EMR | CSEP Physical Activity Guidelines for Adults 18-64 Years (2017) |  | Comprised of 2 components: (1) the PA screening questionnaire and (2) the care plan. (1) PA screening questionnaire assesses current frequency and duration engaging in PA, broken down by activity (A). The system generates a PA summary score. (2) The care plan prompts the provider to ask about weekly PA habits, which are compared to PA Guideline benchmarks, and includes fillable/printable sections to facilitate goal-setting (C) and a PA prescription (P). A screening flowchart and a treatment flowchart accompany each component. The PA screening portion takes ~3:45min and the care plan takes ~5:45min to administer. | +/− content, efficiency, understandability, usability, workflow  − navigation, visibility | − knowledge  +/− ability | [28,50] |
| PAAT (Physical Activity Assessment Tool) | Adults accessing care | Adults 18-64 years | Paper |  |  | Self-administered tool to rapidly assess PA in primary care settings and reduce physician time for assessment. The tool defines moderate and vigorous PA, and lists of common types of PA that are stratified by intensity according to the updated Compendium of PA. Measures type, frequency, and duration of MVPA from all four domains of physical activity—leisure, occupational, household, and transportation—in the last 7 days, and asks if this is “more, less, or about the same as usual” activity (A).  Can be completed in 5-7mins while waiting to see a physician. | N/A | N/A | [116] |
| PACE (Patient-Centered Assessment and Counselling for Exercise) | Physicians | Adults with overweight or obesity and/or chronic conditions | Paper and EMR^b^ |  | Trans-theoretical Model | Protocol incl. clear guidance for PA behaviour assessment, behaviour profile, action/relapse prevention plans, provider summary, and provider PA counselling. Behaviour assessment form (1min to complete) determines PA level on an 11-point scale (the PACE scores) to classify individuals by stage of change, i.e., as a precontemplator, contemplator, or as active (A). The protocol incl. provision of information and materials to individuals accessing care, one-on-one counselling (C), and a PA prescription (P). Protocol takes 2-5mins. A 2-3 week follow-up is conducted by telephone or through the mail, focused on reinforcing the themes within the stage-specific protocol. | + satisfaction, efficiency, understandability  +/− content, workflow  − visibility | ↑ knowledge, frequency^*^, ability, PA behaviour^*^  + confidence | [77,78,97–99,118,141,148] |
| PAFES (Physical Activity, Health, and Sports Plan) | Physicians | Adults 18-64 years and adults with chronic conditions | EMR | Government of Catalonia Èguia de Prescripció de l'Exercici Físic per a la Salut (Guia PEFS; 2007) | Motivational interviewing, Trans-theoretical Model | Involves 2 variables incorporated into the EMR (PA screening and advice given). All adults >15 years who visit are screened to determine their PA level and stage of change, paying special attention to those with ≥1 cardiovascular risk factor (A). Stage of change is measured by asking whether an individual engages in at least 30mins of PA 5 days/week and about predisposition to change. Answers classify adults as inactive (precontemplative, contemplative, or prepared stage) or active (active or maintenance stage). Unprepared, inactive adults in “precontemplation” or “contemplation” receive the motivational approach. Inactive adults in “preparation” receive brief advice, specific advice with follow-up, or referral to a local PA program (e.g. a "Healthy Route"). Those in the active or maintenance stage receive reinforcement to prevent relapse (C). Local resources are identified by each municipality to use as assets to support PA advice given by the primary health care team (R). | + satisfaction | ↑ frequency, PA behaviour^*^ | [136] |
| PAHLS | Physicians | Adults with chronic conditions | Paper |  | Trans-theoretical Model | 6-item tool to measure the propensity for behaviour change regarding diet, PA, and weight reduction. On a 5-point Likert scale, 3 items assess preparedness to change PA and 3 items assess capacity to to succeed with the change (A). | N/A | N/A | [191] |
| PAVS (Physical Activity Vital Sign) | Physicians | Adults 18-64 years | Paper^c^  EMR^d^ | U.S. Department of Health and Human Services Physical Activity Guidelines for Americans (2008) | Trans-theoretical Model | Takes <30 sec to be administered and is intended for every visit just as vital signs. Involves assessing PA as a vital sign (A), brief PA counselling (C), PA prescriptions for inactive adults (P), and referrals to a network of PA resources for guidance and support (R). PAVS asks two questions designed to assess light, moderate, and vigorous PA a person does in a typical week. Adults answer 2 questions on a form when checking in for an appointment, or in-person to the medical assistant taking vital signs. Responses are entered into the EHR by medical assistants or the physician. Total mins/week of PA at each intensity are auto-calculated by the EHR by (average mins/day x average days/week of PA). Total MVPA is also calculated. | + content  +/− satisfaction, navigation, usability, workflow  − efficiency | + knowledge  ↑ ability, frequency^*^, PA behaviour^*^  Ø confidence | [71,107,122,130,131,155,192] |
| Pedometer Prescription in EMR | Physicians  Pharmacists | Adults 18-64 years | EMR and pedometer^b^ |  |  | Embedded within the EMR to fit within usual provider work-flow. Physicians prescribe pedometers to adults deemed safe to engage in low-level PA (i.e., walking). Adults fill a pedometer prescription free of charge as they would any other prescription within the health center pharmacy system (P). Pharmacists provide brief counselling on how to use the pedometer, how to increase steps, and offer a flier about PA classes offered within the health system and a step-log (C). Both the physicians and the pharmacists are given brief scripts to assist them with communicating with adults accessing care. | N/A | + frequency | [119] |
| Pressure System Model | Physicians | Adults 18-64 years | Paper |  |  | Printed clinical materials (decision algorithm, decision balance table, list of barriers to counselling and strategies to overcome, status documentation form). Decision algorithm assesses whether adults are engaging in daily PA of ≥30min and are ready to initiate regular PA (A). A tracking form allows providers to note details of assessment and follow-up visits. Decision balance table allows adults and providers to list advantages and disadvantages of increasing PA or maintaining current behaviour (C). | N/A | ↑ frequency^*^, PA behaviour^*^ | [76] |
| Prex | Physicians | Adults 18-64 years | Paper^c^  EMR^d^ |  | 5 A’s Framework, Trans-theoretical Model | Physical prescription form that assesses current regular PA habits (A), provides counselling principles based on the 5 A’s Framework (C), gives instructions to prescribe PA (P), and could be used as a referral to physiotherapists, nurses, or exercise specialists (R). A User Guide was made to enhance adoption and is attached to a block of 20 prescriptions. Demo-software was made in 2002 to make installation in electronic records possible for health care units. | + content, usability, workflow  +/− satisfaction, efficiency | + ability *(adults accessing care)*  ↑ knowledge, PA behaviour^*^  +/− frequency | [106,126,144] |
| RAPA | Nurses | Adults 65+ years | Paper | Centre for Disease Control and Prevention Physical Activity Guidelines (2001) |  | Brief PA assessment tool designed by NPs. A single question assess whether the individual engages in any PA, and 2 questions assess the presence, duration, and frequency of light, moderate, and vigorous PA, 1 question assess strength exercise once or more per week, and 1 question assesses flexibility exercises once or more per week (A). It is validated among adults 50+ years and by geriatricians. Readable at a 6^th^ grade level and is 3 pages long (page 1 describes each PA intensity with associated images of example activities; page 2 has the assessment questions; page 3 shows how to score). | N/A | N/A | [123] |
| REAP | Physicians | Adults 18-64 years | Paper |  |  | Tool assesses PA (A) and is accompanied by a Physician Key that aids providers in discussing adults’ answers and providing tailored counselling accordingly (C). Key incl. sections on at-risk adult populations, and additional evaluation, treatment, and counselling points. To be used at an initial visit or yearly physical and takes 10min for adults to complete. Written at a 5^th^ grade reading level. | N/A | N/A | [27] |
| SNAP | Physicians | Adults 18-64 years | Paper |  | 5 A’s Framework, Trans-theoretical Model, Motivational Interviewing | Protocol includes: “Ask” (identify risk factors), “Assess” (level of each of the SNAP risk factors and their relevance to the individual in terms of health; assess readiness to change/motivation; A), “Advise” (provide written information and brief motivational interviewing; C), “Assist” (provide support for self-monitoring), and “Arrange” (offer referrals, support services, counselling, follow up; R). | + satisfaction, content  − efficiency  +/− workflow | + knowledge, ability, confidence | [153,193] |
| Standardized Computer Software (GOAL Study) | Nurse Practitioners | Adults with overweight or obesity | Electronic |  |  | Standardized computer software that allows for information entry. During health visits, the NP is guided through lifestyle counselling and entering data on PA measurements (A). Incl. goal setting, monitoring using pedometers, and addressing barriers for lifestyle change (C). | + content | ↑ PA behaviour^*^ | [90,91,194] |
| STEP | Physicians | Adults 18-64 and 65+ years | Paper | ACSM Physical Activity Guidelines (1990) | Trans-theoretical Model | Protocol asks adults to ascend and descend 2 small steps at a pace considered normal or comfortable 20 times. HR before and immediately after and the duration are recorded in seconds. HR, time, sex, age in years, and weight in kilograms are entered in the following equation to predict VO_2_ max: VO_2_ max = 3.9 + (1511/time) x (O_2_ pulse [mass/heart rate] x 0.124) − (age x 0.032) - (sex [male = 2, female = 1] x 0.633). VO_2_ max is used as a baseline fitness level to aid counselling and determine an exercise HR (beats per 10 sec) equivalent to 65% VO_2_ max (A). Counselling and support based on the adult’s PA stage of change, including advice on appropriate frequency, intensity, type, and duration of PA (C), and an individualized PA prescription based on step test results (P) are given. | + efficiency | ↑ knowledge^*^, confidence^*^, PA behaviour *(adults accessing care)*  ↓ SB  ↑ knowledge, confidence  + frequency *(physicians)* | [92,93,96,112,138,151,195] |
| Step It Up! | Physicians | Adults 18-64 years | Paper and pedometer^b^ | UK Department of Health and Social Care PA Recommendations (2015) | Behaviour Change Techniques | Motivational tool: A 5min face-to-face consultation using a case report form to assess adults’ self-reported PA (A), followed by informing them whether they meet the PA guidelines. Then, feedback on adults’ PA is given: providers use the case report form as a prompt and written record of the consultation, ask about perceived benefits of, importance of, and confidence to engage in PA, show how to set goals and self-monitor PA, and provide information about local PA resources (C). The key behaviour change techniques were goal-setting, action planning, feedback, and self-monitoring of behaviour.  Pedometer tool: Includes all of the above plus a pedometer, a step chart for self-monitoring of daily steps, and a booklet to set goals, make action plans, and monitor PA. Adults are told that 10,000 steps/day is a good target to aim for and are encourage to use the step chart to set weekly step goals (C). | + satisfaction, content  +/− efficiency, usability | ↑ confidence, frequency^*^  Ø PA behaviour and SB | [75,95] |
| Tablet-based Lifestyle Assessment | Physicians  Adults accessing care | Adults 18-64 years | Electronic |  |  | Developed for the Android operating system to evaluate PA, nutrition, weight, smoking status, and alcohol use (A). Incl. an interactive questionnaire that uses visual and textual feedback dependent on the adults’ responses. Provides immediate feedback on the risks associated with their reported behaviours and encourages following up with their provider, promoting lifestyle discussions between providers and adults accessing care (C). Adults who are classified as having healthy lifestyle behaviours receive a positive reinforcement statement and information about why it is important. | + satisfaction, content | N/A | [196] |

*^a^* (A) assessment; (C) = counselling; (P) = prescription; (R) = referral; (F) = follow-up

^b^ Multifaceted format of tool

^c^ First or primary format of tool

^d^ Subsequent format of tool

^e^ Italicized text in parentheses that follows RQ2 or RQ3 outcomes indicates the population for which those outcomes apply to

^*^ Statistically significant at *p* < 0.05

+ = positive perceptions/association

− = negative perceptions/association

+/− = mixed perceptions/association

↑ = increase in variable

↓ = decrease in variable

Ø = no change in variable

N/A = not applicable/no results

ACSM = American College of Sports Medicine; EHR/EMR = electronic health/medical record; HR = heart rate; PA = physical activity; sig. = significant; SMART goal = specific, measurable, attainable, realistic, time-oriented goal; TMF = theories, models, and frameworks; UK = United Kingdom; VO_2_ max = maximal oxygen uptake; WHO = World Health Organization
