# Supplementary material for: Tools to guide clinical discussions on physical activity, sedentary behaviour, and/or sleep for health promotion between primary care providers and adults accessing care: a scoping review
Source: BMC Prim Care. 2023 Jul 7;24:140. doi: 10.1186/s12875-023-02091-9 (PMC10326959; doi:10.1186/s12875-023-02091-9)
Supplement: Supplementary file 4 — Additional file 4: Sleep tools (n = 1). [file 12875_2023_2091_MOESM4_ESM.docx]

**Multimedia Appendix 4.** Sleep tools (*n* = 1)

| **Tool** | **User** | **Target Population** | **Format** | **RQ1** | | | **RQ2** | **RQ3^b^** | **References** |
| --- | --- | --- | --- | --- | --- | --- | --- | --- | --- |
|  |  |  |  | **Guideline** | **TMF** | **Description^a^** |  |  |  |
| Sleep Health Materials | Pharmacists | Adults 18-64 years | Paper |  |  | 2-phase behaviour change protocol and a checklist to record actions taken to counsel, provide written information, or refer adults to a physician. Phase 1 (motivational protocol): pharmacist training, screening for sleep disorders, referral to a physician for those at high risk for sleep disorder (R), information provision to adults about sleep health behaviour, counselling strategies to influence change in sleep behaviour, and arrangement of follow up (C). Phase 2 (volitional protocol): questionnaire to assess sleep health behaviour (A), referral uptake, impact of service on confidence in sleep. Checklist includes 2 sections on lifestyle (incl. documenting sleep environment issues and shift work), 1 section on medical history, and 1 section on sleep health (incl. sleep duration, dissatisfaction, and quality). | + satisfaction | ↑ knowledge, confidence *(adults accessing care)*  + knowledge, frequency *(pharmacists)* | [13] |

*^a^* (A) assessment; (C) = counselling; (P) = prescription; (R) = referral; (F) = follow-up

^b^ Italicized text in parentheses that follows RQ2 or RQ3 outcomes indicates the population for which those outcomes apply to

+ = positive perceptions/association

− = negative perceptions/association

+/− = mixed perceptions/association

↑ = increase in variable

↓ = decrease in variable

Ø = no change in variable

N/A = not applicable/no results

* incl. = including

TMF = theories, models, and frameworks
