# Supplementary material for: Tools to guide clinical discussions on physical activity, sedentary behaviour, and/or sleep for health promotion between primary care providers and adults accessing care: a scoping review
Source: BMC Prim Care. 2023 Jul 7;24:140. doi: 10.1186/s12875-023-02091-9 (PMC10326959; doi:10.1186/s12875-023-02091-9)
Supplement: Supplementary file 5 — Additional file 5: Multi-behaviour tools (n = 9). [file 12875_2023_2091_MOESM5_ESM.docx]

**Multimedia Appendix 5.** Multi-behaviour tools (*n* = 9)

| **Tool** | **User** | **Target Population** | **Format** | **RQ1** | | | **RQ2^c^** | **RQ3^c^** | **References** |
| --- | --- | --- | --- | --- | --- | --- | --- | --- | --- |
|  |  |  |  | **Guideline** | **TMF** | **Description^a^** |  |  |  |
| **Physical activity and sedentary behaviour tools (*n* = 7)** | | | | | | | | | |
| EMR (Electronic Medical Record) Physical Activity Tool | Physicians | Adults 18-64 years | EMR |  | 5 A’s Framework, Motivational Interviewing | EMR-based tool that helps providers determine adults’ current level of PA and motivation to increase PA and reduce SB (A), counsel according to this assessment (C), create an exercise prescription (P), and refer to an automated telephone PA coach (R). | + usability, workflow | − knowledge | [55] |
| EPR (Exercise Prescription and Referral) | Physicians  Adults accessing care | Adults 18-64 years | Paper | Canadian Society for Exercise Physiology Physical Activity Guidelines for Adults (2011) |  | Formal advice and prescription tool that promotes routine assessment of PA and advice on reducing SB at each clinical encounter (A), guides providers and adults accessing care through the FITT (frequency, intensity, type, and time) principles to prescribe PA (P), offers 4 tips for reducing SB, and assists with the referral process (R). The tool aims to kick-start the PA and SB behaviour change process and takes 1-3min to administer. | + satisfaction, content, efficiency, understandability, workflow | N/A | [26] |
| It’s LiFe! (Interactive Tool for Self-management through LIfestyle FEedback!) | Nurses | Adults with chronic conditions | Electronic, mobile, and pedometer^b^ |  | 5 A’s Framework, Motivational Interviewing, Theory of Planned Behaviour, Goal-Setting Theory, Self-Determination Theory | Monitoring and feedback tool (activity monitor/accelerometer) for daily wear wirelessly connected to mobile phone and Web apps, combined with a counselling protocol (2-4 sessions over 6+ months) delivered by nurses. Nurses assess the adult’s current PA level and readiness to change (A). Nurses then use motivational interviewing, communicate the risks of inactivity and a sedentary lifestyle using an info card, and agree on a PA goal and provide a list of local PA opportunities to the adult (C). Feedback on PA performance in relation to goal is given to the adult by the nurse and the tool during and in between consultations, respectively. Consultations could be standalone (20min) or added to a routine consultation (10min). | + satisfaction, understandability  +/− content, efficiency, usability, workflow  − navigation, visibility | + knowledge, ability  +/− frequency  +/− confidence *(adults accessing care)*  ↑ PA behaviour^*^ | [56,57,65,66,147] |
| The Lifestyle Assessment | Physicians | Adults with chronic conditions | Paper |  |  | Form incl. questions on occupational and leisure PA, television watching (SB), eating, desire and readiness to change, in a variety of question formats incl. yes/no, frequency, and rating scales (A). The form acts as a prompt for discussing specific objectives for behaviour change in the areas that the adult is ready to begin, and provides a written summary of behavioural objectives (C). | N/A | N/A | [197] |
| Paper-based Decision Tool | Physicians | Adults with chronic conditions | Paper |  | Self-Determination Theory, Behaviour Change Techniques | An 8-page, A5 size booklet with information on adults’ cardiovascular risk, diet, PA, and SB (A) to facilitate shared decision-making on lifestyle change. Consultations take about 15min. The tool prompts adults and providers to reflect on the adult’s beliefs, attitudes, and values, and social and physical environments to identify factors supporting or hindering changes in diet and PA, and blank spaces are given for written responses (C). The main behaviour change techniques used are goals and planning (goal-setting, problem-solving, and action planning), and social support (practical and emotional). | + understandability, visibility  +/− satisfaction, content, efficiency, workflow | +/−  Knowledge  − confidence  ↓ PA behaviour and SB | [58] |
| PEM (Electronic Medical Prescription in Portugal) | Physicians | Adults 18-64 years | EMR | World Health Organization Physical Activity Recommendations for Adults (2010) | Motivational Interviewing, Behaviour Change Techniques | 2 questions assess weekly PA via days/week of brisk walking or other MVPA and mins/day engaging in those activities, and 1 question assess daily time spent sitting (A). Assessment Tool features a "traffic light" feedback system (red-yellow-green depending on if the adult meets PA guidelines or threshold for SB). Brief counselling tool incl. 5 single-page “guides” that can be printed or by emailed to adults according to their motivation and PA levels (C). | N/A | + frequency | [67] |
| RADI (Rapid Assessment Disuse Index) | Physicians | Adults 18-64 years | Paper |  | Trans-theoretical Model, Social Cognitive Theory | Three questions measuring sitting time (SB) as well as general moving about and stair climbing behaviours (i.e., lifestyle PA), over the past day, month, and year (A). The tool is self-administered and presented as a matrix in ≤5min, and is completed prior to the provider visit. Higher scores indicate more sitting and less PA. | N/A | +/− frequency | [68,110,198] |
| **Physical activity and sleep tools (*n* = 2)** | | | | | | | | | |
| IWT (Integrated Wellness Tool) | Physicians | Adults 18-64 years | EMR |  |  | Wellness data entered by adults combined with clinical data from the EMR provides risk scores and clinical decision support in multiple areas (incl. PA and sleep). Adults complete brief questionnaires (incl. PA and sleep) on a tablet in waiting room (A), from which the tool generates a risk score. Providers are prompted to review and discuss risk scores with the adult upon opening their electronic chart, and may be prompted to order a screening test, consult, or treat (C). Adults receive printed informational handouts tailored to their risk scores after the visit. | +/− satisfaction, workflow  + usability | Ø ability, PA behaviour | [59] |
| MyHealthKeeper | Physicians | Adults with overweight or obesity | EMR |  |  | Interface with lifestyle data generated by adults graphically displayed on a clinician’s EHR screen. PA is displayed in a line graph as step count (x-axis: day, y-axis: total daily step count. A heat map also displays 4 rows pertaining to morning, afternoon, evening, and nighttime lifestyle behaviours, and columns pertaining to days. Sleep log are displayed as a stacked area graph rather than a heat map, as per clinicians’ requirements (A). Each lifestyle-related health data plot contained a prescription section for the clinician to specify a healthier daily routine for the adult, considering goals such as preventing weight gain, increasing PA, or increasing or decreasing the sleep period. The default prescription for PA is 10,000 steps (P). | N/A | N/A | [103] |

*^a^* (A) assessment; (C) = counselling; (P) = prescription; (R) = referral; (F) = follow-up

^b^ Multifaceted format of tool

^c^ Italicized text in parentheses that follows RQ2 or RQ3 outcomes indicates the population for which those outcomes apply to

^*^ Statistically significant at *p* < 0.05

+ = positive perceptions/association

− = negative perceptions/association

+/− = mixed perceptions/association

↑ = increase in variable

↓ = decrease in variable

Ø = no change in variable

N/A = not applicable/no results

EHR/EMR = electronic health/medical record; incl. = including; PA = physical activity; sig. = significant; SB = sedentary behaviour; SMART goal = specific, measurable, attainable, realistic, time-oriented goal; TMF = theories, models, and frameworks
