# Supplementary material for: Tools to guide clinical discussions on physical activity, sedentary behaviour, and/or sleep for health promotion between primary care providers and adults accessing care: a scoping review
Source: BMC Prim Care. 2023 Jul 7;24:140. doi: 10.1186/s12875-023-02091-9 (PMC10326959; doi:10.1186/s12875-023-02091-9)
Supplement: Supplementary file 6 — Additional file 6: Perceptions and effectiveness outcomes of included discussion tools. [file 12875_2023_2091_MOESM6_ESM.docx]

**Multimedia Appendix 6.** Perceptions and effectiveness outcomes of included discussion tools.

| **Author Information** | **RQ2:**  **Perceptions** | **RQ3:**  **Effectiveness** | **Quality Appraisal** |
| --- | --- | --- | --- |
| **5 A’s Team Tools (5AsT; *n* = 5)** | | | |
| Ogunleye et al. (2015)  Qualitative  Canada | **Satisfaction**: 83% of respondents rated the intervention as either very good or excellent, with the remaining 17% rating it as good.  **Efficiency**: Some providers thought the tool was too long. Issues with sound quality of videos during the learning sessions; the recurrent sessions helped the information sink in and gave providers time to adapt it to their practice.  **Visibility**: Providers liked the way the tool was set up. | **Confidence**: 86% of the providers said they were either “strongly comfortable” or “somewhat comfortable” with the 5As of obesity following the 5AsT intervention, and 91% reported they felt more comfortable discussing weight issues with adults accessing care as a result of the intervention. | 5 |
| Osunlana et al. (2015)  RCT  Canada | **Satisfaction**: All tools were found to be predominantly “most useful” and “moderately useful”, and more than half of providers rated the “weight loss vs. important health and wellness outcome” graph, “stress and eating” info sheet, obesity fact sheets, and 4Ms cards as “most useful”.  **Understandability**: The need for simple, flexible, and appropriate assessment tools for the primary care context was identified. The 5AsT tool kit was used for this reason.  **Visibility**: The need for clear visuals to avoid misinformation about weight loss expectations in adults accessing care was identified. This was why the “weight loss vs. important health and wellness outcomes” graphs were created. | **Confidence**: The importance of all providers giving consistent messaging and feeling confident in identifying and addressing the root causes of obesity outside their core discipline was highlighted. The “4Ms of obesity management” core messaging for interdisciplinary team-based care booklet was created for this purpose. | 3 |
| Asselin et al. (2017)  Qualitative  Canada | **Satisfaction**: Providers frequently reported that they loved the approach or thought it was valid and applicable to their practice.  **Content**: Providers reported that they believed in and accepted core program messaging.  **Workflow**: The intervention improved visit organization, comprehensiveness and follow-up. | **Knowledge**: Providers reported that the intervention revealed their intrinsic biases, with increased self-awareness leading many to reframe obesity as a chronic disease rather than a lifestyle choice; providers noted increased empowerment from partaking in the intervention, which armed them with effective obesity management knowledge and supported them as change agents in their clinics.  **Ability**: The 5As approach has been shown to improve providers’ willingness and efficacy to provide obesity management and counselling and to support weight loss; the intervention allowed for improved clinical practices.  **Confidence**: Providers reported increased willingness to initiate conversations about obesity management and specifically cited intervention content as the source of their enhanced confidence.  **Frequency**: Providers noted that asking permission to discuss obesity was among the easiest changes they made following the intervention.  **(Other) Barriers:** Many providers cited colleagues' different values and lack of willingness to change as major barriers to implementing the tool’s approach. | 5 |
| Noel et al. (2018)  Qualitative  Canada | **Satisfaction**: Provider: "I had fun. The tool helped, to not be focused on stress for him, and instead focus on his concerns."; "It is a nice way to start talking. It helps focus"; "it is a good conversation starter".  **Content:** It was identified that the tools should provide a structure but allow for different content and details, and should help understand adults accessing care from a holistic point of view.  **Efficiency**: Providers identified that mnemonics can be useful for learning a framework, but might not be as useful when communicating with adults accessing care.  **Understandability**: The tools were seldom introduced, and their function was not explained. If the tools are supposed to help guide conversations between adults and providers, then both parties should be aware of the tools' functions. The need to identify terms that adults did not understand (e.g., metabolic), as well as identifying words which had a negative connotation to adults, and replacing them with more positive words was recognized.  **Usability**: The tool offered flexibility and allowed for different ways of writing. The tools gave focus to the conversation in the clinical encounter, making it easier to identify and interpret important information.  **Visibility**: The tools helped reduce memory load. They provided guidance through steps, note taking and by visually organizing information; A physician noted that tool 4 was too busy.  **Workflow**: The toolkit provided a structure for conversations. It was stated that while the tools are numbered and sequential, the conversation is more organic and dynamic. | **Knowledge**: Providers needed support to gain knowledge about medical conditions, mental health, the occupation, and income of adults accessing care.  **Ability**: Although the tools guided conversations, it was identified that providers bring the skills that make the use of the tool effective, such as digging in, or working iteratively to understand and re-define goals. The providers' skills affect the effectiveness of the tools. | 5 |
| Campbell-Scherer et al. (2019)  RCT  Canada |  | **Confidence**: Provider confidence, their personal views on obesity management, role identity and both their inter-professional relationships and relationships with adults accessing care were found to affect providers’ uptake of the intervention.  **Frequency**: The intervention group had a non-statistically sig. 30% increase in the point estimate of the number of obesity visits conducted over the 6-month intervention. | 5 |
| **10 Simple Steps (*n* = 2)** | | | |
| Parekh et al. (2014)  RCT  Australia |  | **Movement Behaviours**: No group showed statistically sig. improvement in PA or BMI at 12 months after the intervention. | 3 |
| Dickfos et al. (2015)  Qualitative  Australia | **Satisfaction**: Physicians believed the tool was a valuable resource that increased awareness about health behaviours among adults accessing care.  **Efficiency**: Time was cited as a barrier, proposed solutions included: longer consultations (*n*=6; 46%); health behaviour change focused consultations (*n*=5, 38%); computer-based reminders (*n*=2,15%); and the use of allied health personnel (*n*=1,7%).  **Workflow**: 1/3 started counselling opportunistically - when the presenting complaint related to lifestyle behaviour, although some noted the adult's reason for their visit could be a barrier to discussing lifestyle. All physicians agreed the project placed no additional load on their practice and the majority indicated they would consider use of a similar intervention in the future. | **Knowledge**: Physicians believed the project had improved the knowledge of adults accessing care and acted as a trigger for discussion.  **Ability**: Physicians perceived their role in lifestyle change as ‘educators’ (100%), ‘supporters’ (*n*=8, 61%) and ‘prompters’ (*n*=7, 53%); 46% believed they only played a small role in behaviour change, 53% believed the efficacy of advice was largely dependent on adults accessing care.  **Frequency**: PA was addressed by nearly all physicians, with 38% addressing it first. | 5 |
| **10 Top Tips (*n* = 1)** | | | |
| Burr et al. (2020)  Quantitative Descriptive  USA | **Content**: “Walk off the weight” and “up on your feet” tips were considered helpful by 40% and 51%, which was low compared to other tips.  **Usability**: All 13 providers said they would be likely or very likely to use the tool. | **Movement Behaviours**: Average Weight Control Strategies Scale score increased from 1.21 to 2.13 (z=-4.883, *P*=.0001)  **(Other) General Health:** Mean weight loss of 1.17 kg (2.58 lb) (z=0.153, *P*=.8786) and sig. decrease in BMI after 12 weeks. | 5 |
| **10,000 Steps Counselling Materials (*n* = 1)** | | | |
| Eakin et al. (2004)  Quantitative Descriptive  Australia | **Usability**: Post-intervention, 81% of providers indicated that they planned to continue using the brochures and 68% planned to continue using the pedometers. | **Knowledge**: Providers awareness of the 10,000 Steps project increased sig. from 46% at baseline to 97% at the 14-month follow-up (*P*<0.001).  **Frequency**: No sig. increase in % of providers counselling on PA. Post-intervention, 33% of providers reported that they used the materials to counsel on PA. 26/37 providers who completed the post-intervention survey loaned pedometers to adults accessing care. | 4 |
| **ACT (Activity Counselling Trial; *n* = 4)** | | | |
| Albright et al. (2000)  RCT  USA | **Satisfaction**: 83% of respondents said the ACT was an asset. 73% of respondents' overall impression of the protocol was 'good' or 'very good'.  **Efficiency**: 63% of respondents said there was little to no increase in the length of visit.  **Workflow**: 88% of respondents said using the protocol did not impose a burden to clinic operations. | **Ability**: 64% of physicians said the protocol somewhat, much, or very much improved their ability to provide PA advice.  **Frequency**: 99% of physicians gave initial ACT advice. 97% of ACT physicians initialed the form.  Other: Time spent delivering the protocol was 3-6 minutes for 75% of respondents. | 0 |
| Simons-Morton et al. (2001)  RCT  USA |  | **Movement Behaviour:** At 6 months, all groups showed sig. increases in self-reported PA compared to baseline; no sig. between-group differences (exception: women in Counselling vs. Assistance arm showed sig. higher PA at 6 months, *P*=.01). At 24 months, the % meeting PA guidelines was sig. higher for women in Counselling vs. Assistance (*P*=.005) and for men in Assistance vs. Advice (*P*=.02).  **(Other) VO_2_ max:** Sig. increase in VO_2_ max among women in Assistance and Counselling arms versus Advice arm. | 3 |
| Anderson et al. (2005)  RCT  (USA |  | **(Other) General Health:** Women in Counselling or Assistance arms had sig. reductions in daily stress and improvements in body function satisfaction vs. those in Advice only. Men had reductions in daily stress across all arms. Change in barriers to self-efficacy was sig. associated with reductions in daily stress. | 3 |
| King et al. (2006)  RCT  USA |  | **(Other) General Health:** Sig. predictors of PA behaviour were physiological (Advice arm), demographics and health status (Assistance arm), and psychosocial and environmental (Counselling arm). | 3 |
| **ADAPT (Avoiding Diabetes Thru Action Plan Targeting; *n* = 4)** | | | |
| Mann & Lin (2012) Descriptive  USA | N/A | N/A | N/A |
| Lin & Mann (2012)  Qualitative  USA | **Satisfaction**: Providers noted positive reactions of adults accessing care towards behaviour change samples and web reminders.  **Efficiency**: Providers commented that goal-setting tool led to shorter (5min) and more effective counselling, which took 10min total. | **Movement Behaviour:** Steps per day among adults accessing care increased 4284 to 5250 on average. | 0 |
| Chrimes et al. (2014)  Qualitative  Ireland | **Content**: All supported inclusion of the flowsheet, suggested adding fields to make goal-setting customizable.  **Navigation**: Providers had difficulty navigating the different components and finding certain information.  **Usability**: Positive-to-negative comment ratio of 2.1. Allowing providers to set multiple goals at once may lessen the delay in entering goals.  **Understandability** **and Workflow**: Associated with the largest volume (80%) of negative comments.  **Workflow**: Issues using the document simultaneously with dialog. Instructions for adults accessing care were too formal. |  | N/A |
| Mann et al. (2016)  RCT  USA |  | **Movement Behaviour:** Sig. increase in total daily steps (*M*=1418 vs. 1598, *P*=0.007) and 7-day average steps (*M*=1345 vs. 1646, *P*=0.01) in intervention vs. control over 6 months.  **(Other) General Health:** No differences in intervention vs. control in PA stage of change from baseline to 6 months. Trend towards greater weight loss (Median 1.0 lbs. vs. 3.0 lbs, *P*=0.11) in intervention vs. control. | 3 |
| **ARCH (Activating Resources for Community Health Promotion; *n* = 1)** | | | |
| Flocke et al. (2006)  Mixed Methods  USA | **Workflow**: Systemic barriers (e.g., established routines, tension among competing priorities) prevented practices from fully integrating the Resource into daily routines. Timing of external events (e.g., research) drew attention away from the Resource. | **Ability**: Practices that did not perceive themselves as teams had difficulty implementing the intervention plan.  **Frequency**: Post-intervention cohort reported a greater rate of receipt of advice for PA and weight management (p<0.001) and greater rates of receipt of educational handouts and community programs referrals.  **(Other) Stage of Change:** No sig. difference in PA stage of change post-intervention. | 2 |
| **BETTER 2 [and BETTER WISE] Programs (Building on Existing Tools to Improve [Cancer and] Chronic Disease Prevention and Screening in Primary Care [for Wellness of Cancer Survivors and Patients]; *n* = 5)** | | | |
| Manca et al. (2015)  Descriptive  Canada | N/A | N/A | N/A |
| Sopcak et al. (2016)  Qualitative  Canada | **Satisfaction**: Adults accessing care perceived visits as valuable, necessary and motivating. Not all practitioners perceived the program as valuable.  **Efficiency**: Amount of material was perceived as overwhelming and time-consuming.  **Usability**: Implementation can be facilitated by a "champion" (person in clinic who is committed to and promotes the tool). Mixed views about the cost-effectiveness of the tool. |  | N/A |
| Sopcak et al. (2017)  Qualitative  Canada | **Satisfaction**: All adults accessing care were positive about the prevention visits. Many expressed gratitude and appreciation. Many found the program to be valuable, necessary, and motivating.  **Content**: Adults accessing care commented positively about the personalization of counselling but expressed that follow-up between the 1st and 2nd visit (6 months) was too long. | **Knowledge**: Adults accessing care noted that the practitioners were knowledgeable, gave useful advice, and were well-prepared. | 5 |
| Aubrey-Bassler et al. (2019)  Quantitative Descriptive Canada |  | **Frequency**: 63% of adults accessing care underwent PA screening; 36% received a PA referral.  **Movement Behaviour:** 61% of adults accessing care showed an improvement in PA behaviour (i.e., increased weekly PA, occupational PA, walking pace, readiness, and confidence to change). | 4 |
| Sopcak et al. (2021)  Qualitative Canada | **Content**: Providers and adults accessing care said the tools were useful for facilitating a personalized discussion (bubble diagram and prevention prescription in particular). Both groups said the goal-setting component is empowering for adults accessing care. Tools helped connect adults with resources in their communities and encouraged use of the healthcare team available to them (care map in particular).  **Efficiency**: Providers and adults commented on the importance of having enough time for the discussion. |  | N/A |
| **BFWHW (Bright Futures for Women’s Health and Wellness Initiative; *n* = 1)** | | | |
| Matoff-Stepp (2012)  Quantitative Non-randomized USA | **Satisfaction**: Results suggest that adults accessing care (66%), particularly overweight women, were interested in discussing PA with their provider and that the tools were helpful for goal setting. Most adults responded positively to the tools and reported using them to discuss PA. 78% of providers said they would recommend the materials to their colleagues. 67% said they would like to continue to use the materials.  **Content**: Some providers said the materials were not relevant for some adults accessing care.  **Efficiency**: Nearly three quarters of providers reported that the materials resulted in a longer visit. Site administrators reported lack of time in the clinical visit and lack of reimbursement as implementation barriers. Most providers cited time as a barrier.  **Understandability**: Providers cited a need for more guidance on helping women set goals (39%), referrals (28%), and interpreting women’s health assessments (22%). For some, the materials were too complicated or were unclear.  **Usability**: 67% said using the materials enhanced discussion. | **Confidence**: 83% of providers said they could make a difference in the PA levels of adults accessing their care.  **Frequency**: 56% of providers reported that the materials increased their discussion of PA with adults accessing care.  **(Other) Demographics:** Female participants who self-reported having overweight were sig. more likely than their healthy weight counterparts to want to discuss PA with their provider, χ2(2, N=261)=18.215, *P*<.001. No differences between non-Hispanic white and non-Hispanic black women regarding receptiveness to the tool materials or intention to change PA (*P*=.30), suggesting the tools may be culturally appropriate for both groups. Other providers reported that a few adults were unwilling to fill out the self-assessment, to discuss healthy eating or PA during the appointment, or to set goals. | 2 |
| **CBCS (Computer-Based Counselling System) (*n* = 1)** | | | |
| Becker et al. (2011)  Mixed Methods Germany | **Content**: Adults accessing care reported that the video actors were realistic and relatable; however, those who did not identify with the actors seemed to refrain from using the tool. One was concerned that most video models belonged to higher age groups. Insufficient tailoring minimized perceived usefulness of the tool. Adults said the system does not consider needs of employed persons, and that examples of sports activities included only organized sports.  **Understandability**: No adult accessing care had difficulty listening to the speaker, who was referred to as speaking too slowly.  **Usability**: All adults were able to use the tool without further instructions and found it easy to interact with.  **Visibility**: No adult had difficulty reading the text modules.  **Workflow**: Participants expressed being uncomfortable using the tool in the waiting room (felt too close to others, would prefer sufficient time and privacy to use the tool, with most preferring to use the tool at home). | **Confidence**: Mean increase in self-efficacy from 3.19 to 3.39 at 6-week follow-up (*P*=.81). Most adults accessing care were skeptical of acting on the tool recommendations and expressed resistance via rationalizing (perceiving barriers like age-related frailty, disability, or bad weather), claiming the system would patronize them, or denying relevance of the information to their own lives.  **(Other) Attitudes:** Sig. positive change in the affective (+ 6.25, *P*<.05) and cognitive (+7.09, *P*<.05) attitude components from the baseline to the time point directly after using the tool. | 5 |
| **CD-ROM Tool for Managing Obesity in Primary Care (*n* = 1)** | | | |
| Nanchahal et al. (2009)  RCT  UK | **Satisfaction**: Majority of participants reported the structured support groups as very or extremely helpful. A higher proportion in the structured support groups reported satisfaction with level of weight loss, meeting their expectations, and achieving their goals. Majority of participants (55/97, 56.7%) said they would like monthly support. They found that participation changed their lifestyle, gave focus-identified issues to improve upon, made them walk more, and gave a firm goal and deadline, and that seeing the graphs going down on computer made them feel good.  **Content**: Benefits reported included “knowing I was going to be weighed”, “pedometer encouraging extra exercise”, “setting daily goals, and “encouragement from the nurse”. Pedometers were helpful for seeing actual activity level and influencing participants to walk more, but some found it uncomfortable (often dislodged) and tiresome to wear or kept forgetting to put it on. | **Frequency**: Most adults accessing care in the pedometer group reported using the pedometer every day (28/44, 63.6%), or on most days (15/44, 34.1%).  **(Other) General Health:** Adjusted mean difference in weight in structured support compared to usual care groups was −2.63 kg and in the pedometer compared to no pedometer group it was –0.11 kg. 34% of participants in the structured-support groups lost 5% or more of their initial weight vs 18.9% in the usual-care groups; provision of a pedometer made little difference (14/48, 29.2% pedometer; 13/55, 23.6% no pedometer). Difference in waist circumference change in structured-support vs. usual-care groups was −1.80 cm (95% CI=−3.39 to −0.20 cm), and between the pedometer and no pedometer groups was −0.84 cm (95% CI=−2.42 to 0.73 cm). Weight loss was associated with improved weight-related quality of life, self-esteem, and health status, and decreased weight-related symptoms, degree of ‘bothersome-ness’, depression, and anxiety. | 4 |
| **CDSS (Clinical Decision Support System) for PA and Diet (*n* = 1)** | | | |
| Minian et al. (2020)  RCT Protocol  Canada | N/A | N/A | N/A |
| **The Change Program (*n* = 1)** | | | |
| Sturgiss & Douglas (2016) Qualitative Australia | **Content**: No provider wanted educational materials to go along with the toolkit (i.e., felt the toolkit was self-explanatory). Providers commented that the toolkit lacked prescriptive information, although some preferred a more "loose" approach. Some commented that the toolkit would need to be modified for the population they work with (i.e., not generalizable).  **Navigation**: Some commented that there was too much text, it was difficult to read and follow.  **Usability**: Majority of providers thought the program looked usable at face-value.  **Workflow**: All providers stated they wanted the program integrated into the EHR. Stakeholder representatives expressed concern that physicians would not implement the program (i.e., forget about it). It should be noted that providers expressed the opposite sentiment. | **(Other) Cost:** Providers expressed concern that following the recommendations would introduce a financial burden for adults accessing care. | 5 |
| **Computer-based Lifestyle Intervention Concept (*n* = 3)** | | | |
| Carlfjord et al. (2009)  Quantitative Descriptive Sweden | **Usability**: 88% of adults accessing care found the test easy to perform. | **Frequency**: 3065 tests were completed, representing 5.7% of the individuals visiting the primary health care units during the period. Approximately 25% had low levels of PA. | 4 |
| Carlfjord et al. (2010)  Mixed Methods  Sweden | **Satisfaction**: 85% found it positive to refer to the computer-based test.  **Content**: 93% of those who had read the written advice generated by the computer agreed with the advice provided.  **Usability**: Some providers commented that they would use the tool if they spent more time with adults accessing care. | **Knowledge**: Providers commented that more education would be helpful to ensure advice is evidence-based and standardized.  **Ability**: 66% agreed that the test resulted in more adults receiving PA (or alcohol) advice; 68% of respondents agreed it has become easier to address lifestyle issues.  **Confidence**: 78% of respondents had confidence in the computer-based test. | 3 |
| Carlfjord et al. (2012) Qualitative Sweden | **Satisfaction**: Providers expressed satisfaction in how lifestyle conditions had been handled.  **Usability**: Providers commented that perhaps the tool is best for use in follow-up visits; they also commented that the tool does not add anything new and would be a complement to other activities.  **Workflow**: Providers commented that it is difficult to initiate use of the tool and a conversation about lifestyle when the adults has sought care for something else; they also commented that the toll should be designed with it in mind that on first contact with adults accessing care, a thorough history and physical examination will come first to rule out serious illness. | **Knowledge**: Providers commented that adults often have false expectations about preventative care and that they are usually looking for a ‘quick fix’.  **(Other) Motivation:** Providers commented on the need for adults to be motivated to choose (i.e., have autonomy) whether or not they will accept preventative care using the tool. | 5 |
| **Computer-tailored Health Communication Program (*n* = 1)** | | | |
| Sciamanna et al. (2004)  Qualitative  USA | **Efficiency**: The program placed an additional time burden on staff who already felt overworked.  **Understandability**: Staff were inexperienced with the program.  **Usability**: There were technical problems with the computer and/ or printer.  **Workflow**: The program was viewed overall as inconsistent with practice workflow. Only 1 of 10 offices was able to incorporate the program successfully into their office workflow. | **Frequency**: Of the last 50 adults accessing care seen by each of the providers in the study, only providers at one of the practices had more than half of adults accessing care use the program on the day of their visit (either Time 1 or Time 2). | 2 |
| **CONNECT (Consumer Navigation of Electronic Cardiovascular Tools; *n* = 2)** | | | |
| Neubeck et al. (2016)  Mixed Methods Qualitative Australia | **Content**: 4 feasible suggestions were made about content (no additional details provided); all were addressed. Goals and health information were personalized and integrated in the EHR; rewards and encouraging messages included guideline-derived health information is considered trustworthy.  **Navigation**: It was deemed that minimum steps were required to move between tasks.  **Understandability**: 16 feasible suggestions were made about language/clarity (no additional details provided); all were addressed. The risk dial (shows how behaviour affects heart health) was seen as simple.  **Usability**: 4 feasible suggestions were made about functionality (no additional details provided); all were addressed.  **Visibility**: The goal achievement tally on the home screen was deemed a persuasive feature.  **Workflow**: Provider approaches use of program and can see the progress of adults accessing care. |  | N/A |
| Redfern et al. (2020)  RCT  Australia |  | **Knowledge**: Sig. improvements in adults accessing care meeting the pre-defined threshold of high e-health literacy ("skill at using information technology for health") in the intervention vs. control arm (72.6% vs. 64.0%, *P*=0.016)  **Movement Behaviours:** Sig. more adults meeting recommended levels for PA (87 vs 79.7%, *P*=0.02) in the intervention than the control group | 4 |
| **CTH (Connection to Health; *n* = 1)** | | | |
| Hessler et al. (2019) Quantitative Descriptive USA | **Workflow**: Key reasons reported by member of the healthcare team for adults not completing action plans were clinically based, primarily insufficient time due to workflow. | **Knowledge**: Lack of knowledge or comfort with adults accessing care around specific areas of self-management. | 4 |
| **EASY** **(Exercise Assessment and Screening for You; *n* = 2)** | | | |
| Resnick et al. (2008a)  Descriptive  USA | N/A | N/A | N/A |
| Resnick et al. (2008b)  Quantitative Descriptive  USA |  | **Movement Behaviours:** Those who responded affirmatively to at least 1 item on the tool were more likely to engage in exercise than those who did not (*X^2^=*8.0, *P*=.01)). Scoring positively on any of the items (i.e., that individuals who recognize they have problems such as cardiovascular disease, musculoskeletal pain, or dizziness) does not seem to inhibit older individuals from exercising. Item 6, which asks participants to indicate if there is a reason not mentioned why they would be concerned about starting an exercise program, was the only item to sig. predict who was likely to exercise. Those who responded affirmatively were 26% less likely to exercise. | 4 |
| **eCHAT (*n* = 2)** | | | |
| Goodyear-Smith et al. (2013)  Quantitative  Descriptive  New Zealand | **Satisfaction**: Previous studies have found high acceptability of the CHAT among adults accessing care when assessed by 50 consecutive adults from 51 urban and rural practices (2543 adults), with less than 1% objecting to specific questions. Providers were also positive.  **Content**: Evaluation of the initial eCHAT deployment with 196 adults accessing care (91% response rate) found that domains for which adults wanted immediate help were anxiety (9%), depression (7%), PA (6%), and smoking (5%), which was not overwhelming for physicians.  **Understandability**: The majority found the iPad easy to use, the questions easy to understand and appropriate, and did not object to questions. | **Frequency**: Very positive, and the practices have continued to use eCHAT regularly since the research was completed.  **(Other) General:** CHAT is feasible for use in primary care and community settings. Most of the screening questions have sensitivities of 81-96% and specificities of 74-97%. The physical inactivity question had lower sensitivity and specificity because of its format, which has been improved. | 5 |
| Elley et al. (2014)  Quantitative Descriptive  Canada | **Content**: 49% of adults accessing care commented that they thought the tool questions were simple, clear, thorough, relevant, and important; raised awareness of the issues; and showed concern. One commented that the tool raised hopes of getting help. 17 wrote about what they did not like. Some thought the questions were too general; lacked flow; were not long enough; or should include other options such as sometimes, whether the issue was already being addressed, or room for comments.  **Efficiency**: 102 risk factors were self-identified for which adults requested “help today” during 161 consultations (38 issues at 98 consultations in the urban academic practice and 64 issues at 63 consultations in the inner-city practice). If many of these issues were over and above the reason for the consultation, this might present an extra demand on time, either of the physician or of other providers.  **Usability**: 5 physicians commented about possible disadvantages or suggestions for improvement, such as adding a place for the date, other screening tools, or the option being addressed for each issue. One thought it would not be useful for follow-up, and another thought it too simple. All physician respondents stated that they would use such a form, if available. 3 thought they would screen all adults, one would use it opportunistically, and 4 said they would use another approach.  **Visibility**: Some adults accessing care found the questions difficult to read and required their reading glasses.  **Workflow**: 8 physicians completed a form and 7 made positive comments. They liked that it facilitated discussion about potentially sensitive issues that otherwise might not have been talked about, and that adults had the option of “naming” an issue but indicating that they did not wish to discuss it now. | **Confidence**: Participant comments suggested that the form also acted to increase awareness of the issues and provided an impetus for self-reflection, which might represent an intervention. | 3 |
| **eHealth Tool (*n* = 1)** | | | |
| Agarwal et al. (2020)  RCT  Canada | **Satisfaction**: Most adults accessing care reported being satisfied with their PA discussion irrespective of team, with none indicating they were dissatisfied.  **Efficiency**: Nearly half (86/176), 48.9%) of the adults who estimated the length of their PA discussion reported a length of 2 to 5 min. | **Frequency**: 49.4% of adults accessing care stated they received a PA prescription, with only 47% receiving the full intervention including tailored resources from their provider. No sig. differences in the proportion of adults who received at least a PA prescription versus no materials between teams (χ2 3=3.0; *P*=.39). Only 6.6% of adults who completed a process evaluation indicated that no PA discussion occurred during their appointment.  **Movement Behaviours:** Results show a trend toward improvement in PA levels for those who received the intervention, although the unexpectedly high variability limited statistical power. Non-sig. positive difference in MET-mins/week reported at follow-up between intervention and control (mean difference 1027; 95% CI −155 to 2209; *P*=.09). After excluding outliers, linear regression yielded a non–sig. and less positive (closer to the null) difference in grand mean number of MET mins/week reported between intervention and control (MD 487, 95% CI −298 to 1273; *P*=.22). | 3 |
| **EMPOWER-H (*n* = 1)** | | | |
| Lv et al. (2017)  Quantitative Non-randomized  USA |  | **Movement Behaviours**: EMPOWER-H sig. increased mins of aerobic exercise (*P*=.03), whereas mins of stretching or strengthening remained the same (*P*=*.*91).  (Other) EMPOWER-H sig. reduced both office and home SBP and DBP (both *P*<.001), decreased office-measured weight (*P*=.002) and BMI (*P*=.01) and consumption of high-salt and high-fat foods (both *P*<.001), and increased fruit and vegetable intake (*P*=.01), and hypertension knowledge (*P*<.001). 55.9% of participants achieved BP goals (<140 mm Hg for SBP and <90 mm Hg for DBP) at 6 months (*P*<.001). Smoking status and HR QOL showed no sig. changes. | 1 |
| **EMR (Electronic Medical Record) PA Tool** **(*n* = 1)** | | | |
| Dedier et al. (2014)  Quantitative Descriptive  USA | **Usability**: Most agreed or strongly agreed the 3 EMR tool functions were easy to use and were worth the time spend to use them.  **Workflow**: Most agreed that the 3 EMR tool functions should be made a permanent part of the EMR. | **Knowledge:** Providers’ greatest reservation about PA referral was not knowing enough about the Coach to confidently refer adults accessing their care. | 0 |
| **EPR** **(Exercise Prescription and Referral; *n* = 1)** | | | |
| Fremont et al. (2014)  Quantitative Descriptive  Canada | **Satisfaction**: All agreed or strongly agreed that the tool was applicable to the populations of adults accessing their care.  **Content**: EPR is detailed, and evidence based.  **Efficiency**: All agreed or strongly agreed that the tool was an effective use of their clinical time with adults accessing care.  **Understandability**: All agreed or strongly agreed that the tool was easy for adults accessing care to understand.  **Workflow**: All agreed or strongly agreed that the tool was easy to use in their practices. |  | N/A |
| **EVS** **(Exercise Vital Sign; *n* = 5)** | | | |
| Coleman et al. (2012) Quantitative Descriptive  USA |  | **(Other) Validity:** Within 18 months of implementation, 86% of adults who had at least one outpatient medical visit had an EVS measure. Reports of PA were lower than reported in national population-based surveys (31% vs. 60% and 50%) but followed similar patterns, indicating good face validity. Those who were older, of a racial/ethnic minority, had obesity, and had higher disease burdens were more likely to be inactive, suggesting that the EVS has discriminant validity. | 5 |
| Grant et al. (2013) Quantitative Non-randomized  USA |  | **Frequency**: Physicians were more likely to document exercise in their progress notes during visits where EVS was implemented (26.2% vs. 23.7%, including 29.4 % vs 26.0 % among adults with obesity). Progress notes increased by 12% among practices that implemented EVS. Increase in referrals when EVS was implemented (2.1% vs. 1.7% of visits); this difference was greater among adults with obesity (4.0 % vs. 3.2 % of visits). In a multivariate model adjusted for demographic differences and repeated measures, the per-visit odds of referral increased by 14 % (OR 1.14, 95 % CI: 1.11–1.18) among practices that had implemented EVS. More respondents with EVS visits reported that their physician discussed exercise than respondents with non-EVS visits (88 % vs. 76 %, *P*<0.001). After adjusting for age and baseline BMI, adults were 14% more likely to report that their provider discussed exercise (aRR 1.14, 95 % CI: 1.11–1.17) after a visit to an EVS practice (*n*=2,312) compared to a visit to a non-EVS practices (*n*=4,568, *P*<0.001).  **(Other) General Health:** Adults at the EVS medical centres lost more weight over the study period compared to those in the non-EVS medical centers (adjusted difference-in-differences  0.16 lbs [95 % CI: 0.10–0.21], *P*<0.001). EVS primary care practice was associated with 0.06% favourable decline in A1c in adults with diabetes. | 3 |
| Liu et al. (2017) Quantitative Descriptive  USA |  | **(Other) Validity:** Positive associations between PA and measures of disease severity, functional performance, symptoms, and QOL observed were consistent with previous studies using PA surveys or accelerometers (supports the validity of the EVS). Adults with higher levels of PA tended to have higher predicted forced expiratory volume in 1sec (*P*<0.01), less severe COPD (*P*=0.03), and fewer comorbidities (*P*<0.001) and were less likely to use supplemental oxygen (*P*<0.001). Functional performance measures were also associated with the best EVS categories. Adults who were more active had incrementally higher 6min walk test distance (*P*<0.001) and higher step counts (*P*=0.04). Symptom burden (*P*=0.01) and depressive symptoms *P*<0.01) were sig. associated with level of PA, but dyspnea (*P*=0.30) and anxiety (*P*=0.48) were not. Those with higher levels of PA had higher QOL (physical, *P*=0.04; mental, *P*=0.05) compared with those who were less active. | 4 |
| Kuntz et al. (2021)  Quantitative Descriptive USA |  | **(Other) Validity:** There was a positive correlation between the MVPA mins/week reported through EVS and through accelerometry (r =0.38, *P*<0.0001). When restricted to the subsample of 223 participants with <150mins/week, the strength of the correlation decreased (r=0.21, *P*=0.0013). Fair agreement was observed between EVS and accelerometry-based moderate-to-vigorous PA categories (weighted k=0.29). Highest agreement occurred for those with PA level ≥150mins/week. EVS correctly classified 68% and 67% of adults who did and did not meet PA guidelines, respectively. Positive and negative predictive values for EVS compared with accelerometers were 61% and 73%, respectively. | 4 |
| **Exercise Prescription Model (*n* = 1)** | | | |
| Lucini & Pagini (2021)  Descriptive  Italy |  |  | N/A |
| **Food and PA Habit Inventory (*n* = 1)** | | | |
| Sassano (2004)  Quantitative Non-randomized  USA |  | **Movement Behaviours:** Sig. mean changes in PA for various intensities and durations. Mins spent walking or in “other” PA sig. increased across the intervention period (*P*<0.05). | 0 |
| **GDRS** **(German Diabetes Risk Score; *n* = 1)** | | | |
| Jacobs et al. (2018)  RCT  Protocol  Germany | N/A | N/A | N/A |
| **GEM** **(; *n* = 2)** | | | |
| Mateo et al. (2018) Qualitative USA | **Satisfaction**: Tool language may elicit negative emotional response (e.g., shame, guilt).  **Content**: Veterans and Veterans Affairs staff felt positively about using goal-setting for behaviour change.  **Efficiency**: Providers faced time constraints when discussing goal setting during the visit.  **Understandability**: Purpose of questions is not always clear, prompting adults accessing care to ask questions.  **Usability**: Provider burden increased when they had to work with adults to scale goals down. Knowledgeable individuals should be available to assist adults in using technology (i.e., Health Coaches).  **Workflow**: Lack of effective or standardized way to record adults’ goals and communicate them to the health team. |  | 5 |
| Viglione et al. (2019)  RCT  USA | **Satisfaction**: GEM participants rated the quality of counselling 9.1/10 on average. 79% reported that duration of counselling (mean 24 mins) was “just right”. | **Movement Behaviour:** No sig. change in PA behaviour between the usual care and intervention groups.  **(Other) General Health:** GEM participants lost more weight (mean 0.8kg) compared to usual care. No sig. difference between groups in self-efficacy, motivation, intention to change. | 3 |
| **GOALS** **(Goal-focused Online Access to Lifestyle Support; *n* = 1)** | | | |
| Mishuris et al. (2016) Qualitative USA | **Content**: Although providers were very interested in the details of the weight, calories, and activity graphs, they also appreciated a quick view look at how those things interacted with each other and to engaging adults accessing care. This led to designing a dashboard that allowed for honing in to the specific data for each content area.  **Navigation**: Users generated multiple suggestions for improvements. For example, the initial display was a dashboard with 3 dials (exercise, calories, weight); most implicitly understood this, but immediately wanted greater detail and navigated to the graphs with data over time. Most tried to click on the pictorial display from the dashboard to access these graphs, but this was not enabled for navigation at that time.  **Understandability**: To improve usability and understanding of the data, the data visualization of data entered by adults from GOALS in the EHR evolved from bar charts to radar plots to speed dials. Many providers did not grasp the difference in timeline between the different graphs, although this was an important point to see trends over time and relationships between the various types of data presented.  **Usability**: Although all providers agreed that the information/tool would be very useful in healthy lifestyle discussions, the training and time availability to review all data with adults accessing care may be better suited to another role in clinic outside of the primary care provider. Many wanted quarterly updates on adults, but more so that the data be available to access and use in real-time during visits. Some identified the possibility of getting alerts when there was new activity from adults in the system.  **Visibility**: Providers wanted the data in the EHR with graphical, easy to interpret displays of the biometric data.  **Workflow**: All providers thought that integrating external health-related data from between visits would help them better understand adults accessing their care and help them in disease prevention. Most wanted to use the data during a visit, but most also wanted to train ancillary staff to use the interactive tool to engage with adults outside of the provider visit. Time constraints on the provider visit were a concern for all. | **(Other) Barriers:** Providers identified the desire to have some in-clinic contact with adults accessing care to initiate the online diabetes prevention program, and wanted to know adults’ goals and how they were developed. Providers needed a sense of the adults’ engagement with the program and wanted a sense of their barriers to success. | 5 |
| **General Practitioner PA Project** **(*n* = 1)** | | | |
| Porter et al. (2002) Quantitative Non-randomized Australia |  | **Movement Behaviours:** Mean number of times walked in the last week was 4.2 at pre-test and 4.5 at post-test (not statistically sig.). At pre-test, 17% of adults accessing care stated they had not walked for 10min in the last week (decreased to 8% at post-test). Mean number of mins spent walking in the last week increased from 123mins at pre-test to 143mins at post-test (t=2.60, *P*<.01). Number of times adults had done any vigorous PA increased between pre- and post-test from a mean of 0.8x in the last week to 1.1x (not statistically sig.). Slight increase in leisure time PA from pre- to post-test, with a mean of 1.2x/week to 1.5x/week (t=2.19, *P*<0.05).  **(Other) Cost:** Total goods and services budget for the project to cover three Divisions of General Practice was $4,200. Staff time allocated to the project was approximately 2.5 days/week for 1.5 years (for planning, implementation, evaluation). The low cost of this intervention is particularly important in terms of local service implementation and relevant to managers in terms of maximising returns on projects. | 1 |
| **GPPAQ** **(General Practitioner PA Questionnaire; *n* = 2)** | | | |
| Ahmad et al. (2015)  Quantitative Descriptive  United Kingdom |  | **(Other) Reliability:** GPPAQ showed 56 % (70/126; weighted kappa 0.57) and 67 % (87/129; weighted kappa 0.63) of controls scored the same at 3 and 12 months, respectively, as they scored at baseline. At baseline 24 % (69/289) achieved PA guidelines according to accelerometry, while 16 % (47/289) were classified as “active”. Percentage classified as active by GPPAQ-WALK was 32% (92/289). GPPAQ had 19 % (13/69) sensitivity and 85 % (186/220) specificity. GPPAQ-WALK had 39 % (27/69) sensitivity and 70 % (155/220) specificity. GPPAQ has reasonable reliability but results from this study measuring validity in older adults indicates poor agreement with objective accelerometry for accurately identifying PA levels. | 4 |
| VanDenToorn (2016)  Quantitative Non-randomized USA | **Efficiency**: On average, screenings and charting of results took 33.24 seconds to complete.  **Usability**: The need to access charting outside of the EHR was a limitation. Visits were observable in the EHR's daily schedule, however these were not visible for individual adults accessing care if the appointment had already occurred. After the completion of the project, it was discovered that appointments made with the Community Health Worker may have occurred the same day as the nursing student-prepared NP appointment. Thus, no appointment would have been seen in the EHR. These adults would have been considered to have not made an appointment, even though they may have occurred. This made it more difficult and time-consuming than expected to track appointments. | **Frequency**: 21/24 adults accessing care were screened for PA level using the GPPAQ. 5 adults who were screened, and 2 who were not screened, were referred to the Community Health Worker for help finding free or low-cost PA opportunities in the community. Only one of these actually made an appointment with the Community Health Worker. | 1 |
| **Green Prescription** **(GRx; *n* = 14)** | | | |
| Kerse et al. (2005)  RCT  New Zealand |  | **Movement Behaviours:** Leisure time moderate PA and energy expenditure increased significantly; total energy expenditure increased by not significantly. A statistically non-sig. increase in the proportion of participants reaching health-related PA goals of 2.5 hours of MVPA/week was observed. The proportion of intervention group participants reaching this goal rose from 14% (*n*=20) to 31% (*n*=40), compared with the control group, in which the proportion of participants increased from 16% (*n*=21) to 22% (*n*=31) over the 12-month follow-up period (*P*=.06).  **(Other) General Health:** Self-rated general health improved for those in the older age group, with vitality and general health scales showing statistically and clinically relevant improvements. | 3 |
| Dalziel et al. (2006) Quantitative Descriptive New Zeeland |  | **(Other) Cost-effectiveness:** One-way sensitivity analyses gave results ranging from $NZ827 per QALY to $NZ37,516 per QALY. The cost per QALY gained was most sensitive to the relative risk of activity gain for the intervention group, the time horizon of the model and the length of intervention benefit. There is a better than 90% likelihood that the GRx is more cost effective than ‘usual care’, provided that decision makers are willing to pay at least NZ$7,500 per QALY. | 2 |
| Sinclair & Hamlin (2007) Quantitative Descriptive New Zealand |  | **Movement Behaviours:** 70% continue to be physically active since starting the GRx program. 56% said they were currently spending more time in PA compared to before being prescribed the GRx; but 19% said they were undertaking less PA. 60% had undertaken at least 30min of PA on 3 days over the last week. 34% met the current PA guidelines. 20% undertook PA every day (7 days in the last week). Participants had been active for 30min on average of 3.4 (2.5) days/week.  **(Other) General Health:** Moderate to large effects were found for responses relating to increased energy levels (OR=24.5, 95% CI=2.8-210.0) and easier breathing (OR=9.7, CI=1.0-94.7) in the participants who reported increased PA compared to participants who reported less or about the same PA levels after the GRx intervention. Effects for the categories of wellbeing (OR=2.5, CI=0.1-42.5), number of medications (OR=2.1, CI=0.5-8.4), body weight increase (OR=0.5, CI=0.1-1.7), body weight decrease (OR=2.0, CI=0.6-7.1), and strength and fitness (OR=2.1, CI=0.2-16.0) between the participants who reported increased PA compared to those whose physical activity remained the same or decreased were trivial to small and unclear. Both groups reported similar increases in mobility after the GRx, but the group that reported more PA were also less likely to report decreased levels of mobility (OR=0.3, CI=0.1-1.2). Reported aches and pains were similar in both groups after the GRx. | 1 |
| Lawton et al. (2009)  RCT  New Zealand |  | **Movement Behaviours:** Both groups increased their PA over the two years. Mean PA levels, however, were higher (*P*=0.01) and a greater proportion reached the target of 150 minutes of MVPA in the intervention group compared to the control group at 12 months (233 (43%) v 165 (30%), (*P*<0.001), with levels declining but still significantly different at two years (214 (39%) v 179 (33%).  **(Other) General Health:** Physical functioning  (*P*=0.03) and mental health (*P*<0.05) were sig. better in the intervention group at 12 and 24 months. No sig. differences between groups in the proportions on anti-hypertensive (*P*=0.90) or lipid lowering (P=0.80) drugs between the groups over the 2 years. | 3 |
| Patel et al. (2011) Qualitative New Zealand | **Satisfaction**: Majority of GPs indicated that one of the most salient benefits to GRx is that it’s a drug-free process. Some GPs said how GRx puts importance on PA as a valid treatment for health gain, as it’s endorsed by GPs and is presented in the same format as a prescription medication.  **Efficiency**: Majority of GPs stated that time constraints of the consultation was the most salient barrier regarding administering GRx’s. GPs discussed how some adults presented with multiple problems/conditions and how this left little or no time for PA counselling or administering a GRx.  **Usability**: (re: the referral to exercise specialist). The support counsellor was seen as beneficial; GPs viewed them as having the time and skills to fully support adults accessing care in initiating and maintaining PA. Some GPs discussed how time constraints in clinical practice can hinder counselling. GPs stressed how the counsellor increased safety and allowed for monitoring of PA levels. Some GPs viewed GRxs as helping lessen the need for drug treatment for depression or lessening the dosage of antidepressants.  **Workflow**: A couple of GPs mentioned sometimes their practice nurse would administer GRxs. Some GPs delegated the more time-consuming tasks (e.g., choosing an activity) to the support counsellor. | **Frequency**: GPs issued GRxs for primary preventative purposes when there was an awareness (or risk for) a certain condition (i.e., diabetes, high BP, ischemic heart disease, hypertension). GRx was also issued for management in adults with arthritis and weight control, also for management for adults with depression.  **(Other) Cost-effectiveness:** Intervention delivery costs were estimated at NZ$3.62 for the initial brief advice and written exercise script and NZ$10.47 for the 6-month follow-up visit, for a total of NZ$14.09 per participant. The total cost was NZ$93.68 per participant when costs were spread across all those allocated to intervention regardless of utilisation. There was no significant difference in indirect costs over the course of the trial between the two groups [rate ratios: 0.99 (95% CI 0.81 to 1.2) at 12 months and 1.01 (95% CI 0.83 to 1.23) at 24 months (P=0.9)]. The cost–effectiveness ratios were NZ$687 per person made ‘active’ and sustained at 12 months, and NZ$1407 per person made ‘active’ and sustained at 24 months. The cost–effectiveness ratios per minute per week of equivalent moderate intensity PA were NZ$2.82 per min sustained to 12 months and NZ$8.58 per min sustained to 24 months. | 5 |
| Elley et al. (2011)  RCT  New Zealand |  | **Movement Behaviours:** PA levels were sig. higher in the intervention group compared to the control at 12 months (median 120 min/week vs 75 min/week) and 24 months (median 105 min/week vs 90 min/week) (*P*<0.01), and a greater proportion reached the target of 150 min of MVPA in the intervention group compared to the control at 12 months (232/544, 43% vs 164/545, 30%) and 24 months (208/544, 39% vs 178/544, 33%) (*P*<0.001). | 3 |
| Patel et al. (2012) Qualitative New Zealand | **Usability**: Barriers to proper use of the GRx reported by many GPs were the chronic health conditions of older-aged adults accessing care that preclude engaging in (and prescribing) certain PAs. Some GPs said they solved this by prescribing alternative PAs. Another barrier reported by most GPs was adults’ lack of transport to get to organized GRx programs or activity venues. Two GPs discussed trying to fix the transport issue by suggesting carpooling with another person in the same program who lives close to them or using mobility vouchers for discounted taxis. | **Confidence**: GPs discussed how the GRx was seen by some older-aged adults accessing care as a barrier as it involved them having to do new activities and think in a new or different way (about PA). One GP discussed that she brought up the GRx at every consultation she had with some of the older adults accessing her care, to get them used to the idea of the GRx and increase their confidence in starting/doing it. | 5 |
| Leung et al. (2012)  Quantitative Descriptive  New Zealand |  | **(Other) Health Care Use:** Pedometer group had 10% more users of hospital specialists (P=.09). No sig. differences in number of health care visits between groups; however, pedometer group had a higher number of visits except for nurses, other allied health professionals, and inpatient days. No sig. differences in mean costs per service between groups. Pedometer group tended to have higher (NZ$118) community care and exercise-related costs than standard GRx group, but this was more than offset by the lower (NZ -$197) hospital-related costs due to less expensive procedures and shorter inpatient stays. Only a NZ$17 difference in exercise-related costs in pedometer group is the cost of the pedometer is excluded. **(Other) Cost-effectiveness:** There were non-sig. differences in both costs and outcomes between the two groups. Incremental Cost-effectiveness Ratio: Additional cost per 30min of weekly leisure walking and per QALY were, when including only (i) community care costs, $115 and $3105, (ii) exercise and community care costs, $130 and $3500, and for (iii) all costs, both negative, indicating lower costs and greater effectiveness, respectively. Thresholds required for the pedometer-based GRx to have a 90% probability of being cost-effective per 30min of weekly leisure walking, compared with the standard GRx, were (i) $620, (ii) $660 and (iii) $370, respectively for the 3 (i-iii) cost categories. When QALY were used as the measure of effectiveness, the pedometer-based GRx, compared with the standard GRx, was statistically cost-effective at the QALY thresholds (i) $30000, (ii) $30500 and (iii) $16500 for the 3 cost categories. At a threshold of $20000 per QALY, the pedometer based GRx has a probability of being (i) 94%, (ii) 93% and (iii) 96% cost-effective for the 3 cost categories.  Sensitivity analysis: If the pedometers costed NZ$20 instead of NZ$50, the incremental cost-effectiveness ratio would decrease between 22 and 33%, meaning the pedometer-based GRx would be cost-effective at a threshold of $20000 per QALY for all 3 cost categories. | 4 |
| Kolt et al. (2012)  RCT  New Zealand |  | **Movement Behaviours:** During the intervention period (3-4mo), the pedometer group increased leisure walking by 63.0 min/week on average, which was more than double the increase in the standard group (30.9 min/week). At 12mo follow-up, pedometer group decreased 13.4 min/week (net increase of 49.6 min/week) and the standard group decreased 2.8 min/week (net increase 28.1 min/week). All participants increased total walking activity over time.  **(Other) General Health:** SBP and DBP decreased in each group over time, but with no sig. differences between. No sig. change between groups in physical function, or over time per group. Neither group had a change in BMI. Physical functioning, general health, vitality, and mental health improved sig. over time, but with no differential changes between groups (other QOL measures showed moderate to strong ceiling effects and were not analyzed). | 3 |
| Patel et al. (2013a)  RCT  New Zealand |  | **Movement Behaviours**: Leisure moderate PA significantly increased over time for participants independent of intervention allocation (Wilks’ Lambda=.86, F(2,225)=19.1, *P*<.0005), eta-squared=.15). Total leisure PA significantly increased over time for participants independent of intervention allocation (Wilks’ Lambda=.86, F(2,225)=17.8, *P*<.0005), eta-squared=.14). Total walking PA significantly increased over time for participants independent of intervention allocation (Wilks’ Lambda=.87, F(2, 224)=17.3, *P*<.0005), eta-squared=.13).  **(Other) Mental Health/Illness**: Depressive symptomology sig. decreased over time for participants independent of intervention allocation (Wilks’ Lambda=.91, F(2,219)=10.4, *P*<.005), eta-squared=.09). General mental health functioning sig. increased over time for participants independent of intervention allocation (Wilks’ Lambda=.94, F(2,217)=.94, *P*<.001), eta-squared=.06) | 3 |
| Patel et al. (2013b) Quantitative Descriptive  New Zealand |  | **(Other) Motives:** Participants being active for enjoyment reasons, for health and medical reasons, and for the purpose of wanting to be physically active accounted for 17%, 17%, and 15% of the variance in PA participation, respectively. Participants with 3+ chronic conditions (3.9 ± 0.42) were more motivated to engage in PA for health and medical reasons compared with those who reported no chronic health conditions (3.3 ± 0.47), F(2, 77)=4.3, *P*=.02, eta-squared=.02. **(Other) Benefits:** Perceived personal (e.g., confidence) and physical (e.g., feeling fitter) benefits of PA participation accounted for 32% and 19%, respectively. Participants aged 76 years and older (3.4 ± 0.46) perceived that they received more personal benefits from engaging in PA than did participants in the 65- to 75-year age group (3.1 ± 0.61), F(1, 78)=6.2, *P*=.02, eta-squared=.01. Older participants (76+ years; 3.6 ± 0.47) reported they experienced more physical benefits as a result of being physically active than did younger participants (age 65–75 years; 3.3 ± 0.51), F(1, 78)=5.2, *P*=.03, eta-squared=.01. Participants with 3+ chronic conditions perceived that they experienced more personal benefits as a result of engaging in PA (3.5 ± 0.41), F(2, 77)=6.9, *P*=.002, eta-squared=.02.  **(Other) Barriers**: Personal (e.g., motivation), perceptual (e.g., feeling too old), and time constraint barriers accounted for 16%, 16%, and 13%, respectively. Participants with 3+ chronic conditions (3.0 ± 0.63) perceived more personal barriers for PA than did those who reported no chronic health conditions (2. 4 ± 0.57), F(2, 77)=3.9, *P*=.2, eta-squared=.02. Compared with normal-weight (2.5 ± 0.55), participants with obesity perceived more personal barriers for PA (2.9 ± 0.61), F(2, 77)=3.4, *P*=.04, eta-squared=.01. | 3 |
| Hamlin et al. (2016) Quantitative Descriptive  New Zealand | **Satisfaction**: Most of the adherence group (78%) were either satisfied (30%) or very satisfied (48%) with their experience with the GRx, leaving only a small portion of dissatisfied participants (9%). Several participants suggested improvements to the program including better connection to activities (15%), more telephone support (13%), and an increased awareness and willingness to prescribe the programme from the GPs. | **Movement Behaviours:** Participants who had fulfilled their GRx reported an additional 64 min (95% CI=16-110) of total PA engagement in the week prior, compared to non-adherence group. 42% of participants in the adherence group reported increased PA levels compared to 29% in the non-adherence group. Participants in the adherence group were less likely to be sedentary than the non-adherence group (OR=0.7, 95% CI=0.5-0.9) and more likely to achieve the current PA guidelines of at least 150 min of PA/week (comprising of 30+ min/day on ≥5 days) (OR=1.1, 95% CI=1.0-1.3). Walking was the most popular activity in both groups with 25.5% walking in the adherence group and 20.2% walking in the non-adherence group.  **(Other) General Health:** 48.4% of the adherence participants reported improved health benefits over the past 2 years, compared to 28.5% of the non-adherence participants. Those who had received a GRx specified their lack of programme engagement was due to medical or injury (35.8%), insufficient time (12.8%), other reasons (10.3%), change of dwelling (7.7%), spousal disinterest (5%), or personal lack of interest (5%). | 3 |
| Patel et al. (2020)  RCT  New Zealand | **Satisfaction**: Most participants in both study groups agreed or strongly agreed that their appointment with their GP for their GRx was a positive experience, and that their GP discussed the best type of PA for them. Most participants (88%) in the pedometer-based group agreed or strongly agreed that their pedometer made them aware of how active they were, and 82% indicated that their pedometer motivated them to be more active and to walk more. Most also agreed that their pedometer was comfortable to wear (82%) and 69% agreed that their pedometer could easily be secured to their clothing. However, only a minority (36%) agreed or strongly agreed that their pedometer was accurate in recording all their steps, and that it measured all their activities (39%). |  | N/A |
| Albert (2020), Qualitative  New Zealand | **Satisfaction**: Participants viewed Te Whare Tapa Whā (4-dimensional health assessment), along with other Maori concepts, as a token gesture or the adding-on of a Maori concept. There is a strong view that this is disrespectful when non-Maori organizations use Maori concepts as add-ons. An add-on is described as the use of Maori concepts that are not the primary strand. | **Knowledge**: The GRx service is not generally well-known to communities and individuals  **(Other) Importance of whanaungatanga (relationship):** The relationship between the participant and the GRx coordinator has been identified as an essential component for participants to engage confidently with the GRx service. Once the relationship is established, there is a perceived belief that achieving success and reaching the individual’s health potential is achievable.  **(Other) Needing whakawhanaungatanga (process of establishing relationships) and a powhiri (to welcome, invite, beckon) process:** Beginning the relationship using whakawhanaungatanga and the powhiri process between the participant and the GRx coordinator is important and helps to address the potential of future communication and engagement barriers.  **(Other) Comfort**: Being comfortable and feeling safe emerged again as essential components for active engagement with the GRx service. | 5 |
| **HausMed Website (*n*** **= 1)** | | | |
| Mehring et al. (2013)  RCT  Germany |  | **Movement Behaviours:** Self-reported PA improved significantly more in the intervention than in the control (*P*=.048).  **(Other) General Health:** Weight decreased on average by 4.2 kg in the intervention group and 1.7 kg in the control group (mean group difference 2.5 kg; 95%CI 1,1; 3,8; *P*<0.001). Reductions for WC and BMI were also sig. larger within intervention (*P*s<.001). | 3 |
| **I-ACE (Interactive Lifestyle Assessment, Counselling, and Education Software;** ***n*** **= 1)** | | | |
| Abu-Saad et al. (2019)  RCT  Israel | **Satisfaction**: Over 90% of the participants in both study arms expressed high satisfaction with the dietician and lifestyle counselling. The study dietician also expressed overall satisfaction with the I-ACE software. She observed that younger participants who were technology oriented engaged more with the software in the counselling sessions than older participants.  **Content**: The dietician noted areas that the software did not address (e.g., emotional distress of coping with multiple comorbidities or other personal issues and other emotional support issues that may impact lifestyle behaviors and the readiness to make lifestyle changes).  **Understandability**: Most participants stated that they understood and could implement/utilize the information provided on general nutritional recommendations, portion sizes, exchanges portions, sample menus, and their progress over time; however, the proportion who indicated that they could only understand or implement these materials partially, or not at all, tended to be higher in the SLA than the I-ACE arm.  **Usability**: Most participants in the I-ACE arm thought that the I-ACE software was helpful to the dietary counselling (91%) and did not detract from their interaction with the dietician (96%).  **Visibility**: The study dietician also expressed overall satisfaction with the I-ACE software. She found the pictorial educational materials and the quantitative nutrient information, provided when individually tailoring sample menus, particularly helpful. | **Movement Behaviours:** The odds of engaging in any leisure PA at 12 months tended to be higher in the I-ACE arm versus standard lifestyle advice arm, but were not statistically sig. (OR=2.8; 95% CI 0.7-11.6; *P*=.16).  **(Other) General Health:** Both arms sig. reduced in HbA1c (*P*<.001). There was a marginally sig. lower intake of added sugar in the I-ACE arm. Within the I-ACE arm, the mean (SE) differences in added sugar and dietary fiber intakes from baseline to 12 months were −2.6% (SE 1.0%) of total energy (*P*=.03) and 2.7 (SE 0.0) g/1000 kcal (*P*=.003), respectively | 3 |
| **It’s Life! (Interactive Tool for Self-management through LIfestyle FEedback!;** ***n* = 5)** | | | |
| Verwey et al. (2012)  Mixed Methods  The Netherlands | **Satisfaction**: Most interviewees liked the idea that using the tool would give both providers and adults accessing care the ability to monitor PA levels. They confirmed the added value compared with self-reported PA because adults often overestimate their level of activity. Participants described the system postiively as “professional”, “motivating”, “valuable”, “customisable” and “innovative”, and negatively as “slow” and 'time-consuming'.  **Content**: Participants liked the use of the graph indicating the level of PA over the past months and they were satisfied with the content of the individual charts. They said that it was useful information and that this could support them when talking to adults during consultations. Nurses felt the most important information should be presented at the top of the page (and the page was too long).  **Efficiency**: The system was deemed valuable and easy to use, and instructing the nurses to use the system was done in a few minutes.  **Navigation**: When registering a new adult accessing care in the system, 3 nurses used the back button of the web browser instead of the back button of the application itself—this caused an error with the connection to the server. Sometimes the system was slow due to Internet connectivity problems. Many nurses commented on the complexity of navigation. Nurses asked whether subpages not necessary to PA coaching could be removed (e.g., medication charts).  **Usability**: Although it was the first-time nurses had used the system, they were mainly positive about the ease of use. Scores on task performance ranged from 5.5-6.6 on a scale from 1 to 7.  **Visibility**: The PA data should be clearly presented and embedded in the information system, or they should be linked with this system. Several nurses complained about using two or more systems and they wanted to avoid ‘double registration’. The system should present a summary of all information about all adults’ performance and goal attainment at a single glance, presented in numbers and graphs. The "more [down arrow]" button in the individual charts with information about the preferences of adults was overlooked by 4/5 participants.  **Workflow**: Most of the nurses were not enthusiastic about giving feedback on the PA levels of adults between regular consultations. A few mentioned that they would probably monitor activity levels to find out whether adults were actually using the tool. They did not, by any means, want to receive push information, such as notifications from the system. | **Ability**: Most nurses indicated that normally, they do not spend much time on the assessment of the level of PA. Therefore, the use of this tool by adults accessing care to assess PA levels objectively was considered valuable. | 4 |
| Verwey et al. (2014)  Mixed Methods  The Netherlands | **Satisfaction**: Most adults accessing care (12/17) were positive about the intervention. They felt encouraged to be more active and mentioned 3 aspects: the awareness of their PA performance, the stimulating effect of the daily target goal and the positive effect on self-efficacy. Opinions stayed positive from the 1st to 3rd interview. 4 participants regretted having to return the tool at the end of the study. Participants characterized the intervention in one word: stimulating (*n*=4), good (*n*=4), fun (*n*=3), positive, meaningful, could be effective, a boost, a helping hand, a big stick.  **Content**: All nurses agreed on the usefulness of obtaining objective PA data via the tool, indicating that it was difficult to assess otherwise. More than 50% of adults noted the first consultation (mostly focused on the tool and less on counselling) had a good atmosphere and was informative and clear. Adults were satisfied about goal setting in the second consultation. 'Minutes' were deemed a more important stimulus than 'intensity'.  **Efficiency**: All nurses indicated that when looking at the data, it was much easier to talk about barriers and facilitators for becoming more active. However, this often resulted in a longer consultation time. The nurses spent more time explaining the tool than on PA counselling.  **Usability**: Problems occurred frequently (18/20 participants) and had to do with log-in difficulties (small keyboard) and connectivity errors (not recognizing if Bluetooth was off or flight mode was on). 6 adults accessing care needed extra advice about how to log in, which was given to them during the first interview and during consultations with the nurse. The connectivity problems were twofold: between the accelerometer and the smartphone, as a result of which the app indicated the activity sometime later, and between the smartphone and the server. This was an issue for nurses as they could not see results on the website and thus adults did not receive feedback sessions. Regarding mins of PA tracked, one adult with severe breathing problems and one with an orthopaedic shoe said that, when active, neither reached a speed of more than 3.5 km/h (tool only 'counts' at 3.5 and up). Their impression was that they had really tried very hard, but the tool had not given them enough minutes as a reward. | **Confidence**: Although most adults accessing care were positive about the tool, the motivation of some dropped when technical problems occurred.  **Frequency**: Information on the server indicated that adherence regarding the use of the tool was high (on average 80%).  **Movement Behaviours:** A total of 12 adults were positive about the effect of the invention on their PA performance and 5 adults were neutral about it; the latter were those who were already sufficiently active. Mean PA sig. increased by 10.6 min/day, from 28.7 (SD 21.1) min/day in the first 2 weeks compared to 39.3 (SD 24.2) in the last 2 weeks (*P*=0.02).  **(Other) Mental Health:** Participants scored higher in QOL and self-efficacy after the intervention, but this was only sig. for QOL. QOL scores increased from 0.76 (SD 0.21) to 0.84 (SD 0.17) (*P*=0.04). Mean QOL at baseline scores of adults with diabetes were 0.2 higher than that of adults with COPD. | 3 |
| van der Weegen et al. (2015)  RCT  The Netherlands |  | **Movement Behaviours:** Directly after the intervention, participants in Group 1 who received the tool and the SSP showed 8 minutes more moderate and vigorous PA (≥3 METS) than participants in the SSP, and 12 minutes more PA than the care as usual group. The group that received the entire intervention (tool + SSP) showed more PA directly after the intervention than Group 2 (SSP) and Group 3 (care as usual). Three months post-intervention, this effect was still present and significant.  **(Other) Mental Health:** After 9 months, Group 2 (SSP) scored sig. higher for the PA of the QOL scale than Groups 1 (tool + SSP) and 3 (care as usual). At end of intervention (6 months), both groups scored sig. higher on the mental component scale compared to the care as usual group. | 4 |
| Verwey et al. (2016a)  Mixed Methods  The Netherlands | **Satisfaction**: All nurses were satisfied with the instruction charts per consultation. 84% of the adults in group 1 and 70% in group 2 were satisfied with the intervention. Participants from group 1 (SPP + tool) were sig. more positive about the intervention than those in group 2 (χ2(2, *n*=113) = 11.17, P=0.004). Those were more explicit in their positive opinions about the intervention, specifically about the fact that use of the tool was fun; it led to greater awareness and more discipline.  **Content**: Group 1 nurses were mainly satisfied or neutral (90%), while the view of group 2 nurses varied more; 67% were satisfied and 33% were dissatisfied with the content of consultations. In group 1, the nurses indicated that the intervention encouraged people to be more physically active because they had more insight into their exercise habits and nurses liked the possibility to monitor adults through the use of the web application. Only 9% (*n*=3) of adults were not satisfied with the sessions [their content]; however, the feedback messages could be improved according to 32% (*n*=19) of participants. Suggestions for improvement of the feedback messages were: more variation (*n*=9), send feedback less frequently (*n*=6), and to make them more personalised (*n*=6).  **Efficiency**: 58% of adults experienced problems using the tool. During the intervention period, 190 issues were registered by the help desk. Most problems occurred with the connection between the activity monitor and the phone (*n*=88) or the connection between the phone and the server (*n*=30). A big issue in the beginning of the trial was that most phones were of a newer type than the one the app originally was developed for.  **Navigation**: Technical problems frequently occurred. 20% of nurses experienced problems using the web application (PA results not available or wrong).  **Understandability**: Seventy-four percent (*n*=13) of the nurses were positive about the SQUASH questionnaire; it gave a clear picture of adults’ activities.  **Usability**: Often the consultations took longer than the intended 20 minutes. Although most adults in group 1 were positive about the tool, the functioning could be improved. Nurses also indicated that the technical problems were demotivating. | **Knowledge**: Use of the tool led to greater awareness of the importance of PA and more enjoyment. The nurses stated that, by performing the intervention, they became more conscious about the PA of participants.  **Ability**: Use of the tool led to greater discipline in carrying out PA. Nurses from group 2 (SSP with NO tool) encountered difficulties for PA counselling because they only had vague ideas of PA levels of adults accessing care.  **Confidence:** Adults in both groups indicated they were more conscious about being physical active and more motivated to exercise.  **Frequency**: Some adults indicated that goals had not been discussed during the consultations. Goals were mainly set by adults themselves (61% *n*=69) or in collaboration with the nurse (32%, *n*=36). In group 1, 88% (*n*=50) of adults used the tool until the end of the intervention. Reasons to stop wearing the tool were: malfunction of the tool, the belief that using it was not necessary anymore because of an appropriate activity level, or quitting the study. The median of number of times that adults (*n*=63) used the ‘remarks of today’s measurement’ session was 30 (IQR=15–52). There were 6 diary sessions on consecutive days. On each day, 70% of adults completed this session. The median that adults (*n*=53) read feedback messages was 23 (IQR=6–35). Nurses: 90% viewed the results using the web application during consultation and 50% viewed results in between consultations. The frequency of use varied from rarely, 2x in total, to 2x/month.  **Movement Behaviours:** Sig. difference between groups 1 and 2 in the responses regarding goal achievement; group 1: 84% *n*=48 and group 2: 61% *n*=34 thought they had reached their goals x2 (1, *N*=110) = 7.50, *P*=0.006. Adults in both groups indicated that they were more physically active and that their physical fitness improved. 75% of the adults in group 1 and 46% of adults in group 2 thought that PA levels were improved (χ2 (2, *n=*110) = 8.18, *P*=0.004). According to group 1 adults, the effectiveness was specifically attributed to the tool (*n*=11).  **(Other) Training:** All nurses were instructed as planned (i.e., the 2-hour instruction), but the web lectures were rarely watched. Nurses in Group 1 (tool) experienced the instruction as too brief, especially to become familiar with the web application. | 4 |
| Verwey et al. (2016b)  Qualitative  The Netherlands | **Satisfaction**: Most respondents liked the idea that using the tool would give both adults accessing care and practice nurse the ability to monitor PA levels.  **Content**: A few practice nurses were not enthusiastic at all about the focus on PA.  **Efficiency**: The role of the GP in lifestyle counselling was regarded as limited (the practice nurse has more time for it). Lack of time and money and heavy caseload were cited as important barriers by practice nurses.  **Usability**: They argued that the tool was probably more suitable for younger adults accessing care who are used to mobile technology.  **Workflow**: Practice nurses had doubts about giving feedback in between consultations. |  | N/A |
| **IWT (Integrated Wellness Tool; *n* = 1)** | | | |
| Foucher-Urcuyo et al. (2017)  Mixed Methods  USA | **Satisfaction**: 92% of adults accessing care indicated that the tool would help their provider understand their current state of health to some extent; providers were not surprised by adults’ risk scores, stating that the tool did not provide any new information; however, it was useful in addressing chronic pain and fatigue.  **Usability**: Almost all adults found the tool easy to use, did not require help, and stated that they would use the tool again. Similar observations were made by adults >80 years of age; Providers also found the tool easy to use and most useful for new encounters and physical exams.  **Workflow**: Providers found that the tool generated more discussion and increased adults’ involvement in their care; discussing wellness required time but in some situations, providers found the tool to streamline the ordering process (ordering tests). Adults were willing to complete questionnaires in the waiting room, thus this did not impact workflow. Providers had concerns about changing their routine, learning new technology, and increased demands on their time with tool. | **Ability**: Providers did not perceive the tool to have improved their ability to care for adults accessing care.  **Movement Behaviours:** Providers did not notice much of adults’ follow-through with lifestyle recommendations after using the tool. | 5 |
| **Life’s Simple 7 and My Life Check (*n* = 3)** | | | |
| Foraker et al. (2014)  Descriptive  USA | N/A | N/A | N/A |
| Murphy et al. (2015)  Quantitative Descriptive  USA | **Usability**: The summary report helped to promote a stepwise, sequential approach to addressing the health factors and promoted adherence to specific participant goals.  **Visibility**: Large font with pictures, diagrams, checklists were positive attributes. | **Ability**: NPs reported feeling well-prepared and moderately effective in providing lifestyle change counselling.  **(Other) General Health:** Sig. increase in older adults’ tool score at program completion. 12% and 8% decrease in SBP and DBP, respectively. Blood cholesterol and weight increased slightly with +4% and +2% change, respectively. 88% of seniors improved their total tool score. Decrease in tool score at the residential facility. Slight decreases in SBP and DBP, a decrease in blood glucose, an increase in blood cholesterol, and unappreciable change in weight. | 4 |
| Yamane et al. (2020) Quantitative Descriptive  USA |  | **Confidence**: Most adults accessing care felt positively about their role in the healthcare decision-making process and its likelihood of success. When asked, “What changes do you plan to make after your visit today?” 12 adults (48%) answered changes to the amount of PA. | 3 |
| **The Lifestyle Assessment (*n* = 1)** | | | |
| Saunders & Pastors (2008)  Descriptive  USA | N/A | N/A | N/A |
| **MIS (Minimal Intervention Strategy; *n* = 1)** | | | |
| Fransen et al. (2008) Qualitative Netherlands | **Content**: Treatment should be tailored to the preferences of both adults accessing care and providers. The tool should pay attention to both healthy nutrition and PA, facilitating long-term behaviour change to a healthy lifestyle; the tool should consist of different types of interventions; The manual was considered comprehensive with sufficient background and in-depth information.  **Efficiency**: Several participants indicated that they thought that it was a lot of work but at a closer look they noticed that all described steps were small.  **Understandability**: Test needed to be shortened, e.g., replacing sentences by keywords was considered sufficient by several GPs; the information needed to be restructured to increase its clarity and workability; overall the materials were considered understandable and interesting but words like “barriers” were perceived as difficult. | **Knowledge**: Treatment needs to be multidisciplinary involving GPs, NPs, physiotherapists, psychologists, and dieticians; the GP is the most suitable person to select the adults during regular medical visits.  **Ability**: The NP is seen as the most suitable mentor for adults accessing care: she/he can guide the adults with the help of the GP and coordinate with other disciplines if necessary.  **Confidence**: Need for professionals to have confidence in their own ability to address overweight and obesity (self-efficacy).  **(Other) Motivation:** Motivation of adults accessing care was identified as one of the most important determinants of treatment success. | 5 |
| **MyHealthKeeper (*n* = 1)** | | | |
| Ryu et al. (2017)  RCT  South Korea |  | **(Other) General Health:** Intervention group who used the mobile app every day and received lifestyle feedback counselling showed sig. higher weight loss than the control group (mean 1.4 kg, *p*<.001). Triglyceride levels (mean 2.6 mmol/L, *P*=.002), and BMI (mean 0.4 kg/m^2, *P*=.000) decreased by end of the study period in the intervention compared to control. | 3 |
| **My Plan (*n* = 3)** | | | |
| Plaete et al. (2015a) Qualitative Belgium | **Satisfaction**: Most GPs were positive about the use of the principles of goal setting, self-regulation, and empowerment.  **Content**: According to GPs, the proposed methods, and techniques to apply the self-regulation principles may effectively lead to behavioural change; GPs supported the idea that adults have to decide themselves which behaviour they want to change, and to select their own health goals about PA and nutrition intake. Most GPs agreed that participants will be more likely to change their health behaviour when they make their own decisions about the extent of behavioural change compared with priori behavioural targets; believed in personalized, written feedback; should be sufficient options for adults in health behaviour choices and action planning.  **Navigation**: Suggested pictograms and pictures so individuals who do not speak the language can navigate through the tool.  **Understandability**: When adults defined health goals that are too difficult or too easy to reach, GPs found it instrumental that the programme would provide appropriate feedback, and advise adults to adapt their goal to a more attainable or challenging goal; when using the tablet in the waiting room, it should be clear what the tablet is meant for. Therefore, GPs suggested using a poster and flyers in the waiting room.  **Usability**: Some GPs came up with the idea to use an existing online platform to send the action plan to the GP. Another suggestion was to integrate the tool in the medical software programmes of GPs.  **Workflow**: The idea that the program could be halted and resumed at home was also approved by most GPs; GPs wanted to be able to select a delivery method that worked best for their working system and for adults accessing their care. | **Knowledge**: GPs reported that it is important to have the opportunity and time to introduce the intervention program to adults accessing their care; have the expertise to give appropriate feedback; others mentioned that they did not have enough expertise for PA counselling.  **Ability**: GPs found it important that they see adults trying to change health behaviour in order to continue PA counselling; Some GPs indicated that they did not want to receive adults’ action plans by email because they would feel responsible to give feedback and monitor goal progress. Other GPs thought this was a good idea because they can evaluate and give feedback on adults’ action plans; they reported that the tablet can facilitate preventative counselling, but they still thought it was difficult to do this for all adults.  **Confidence**: GPs believed that the programme can be effective in short but not long term; thought that follow-up response rate would be low; did not want to use the tablet during consultation due to a lack of time; some GPs were convinced that they are the most appropriate counsellors to provide the programme, because of GPs' high level of authority. Some GPs had doubts about the effectiveness of setting too easy health goals or health goals that do not reach health norms; combination of both flyers and tablets was suggested.  **Frequency**: Most GPs mentioned that they already used this approach and, when applied, experienced that it was successful. | 5 |
| Plaete et al. (2015b) RCT Belgium | **Satisfaction**: Older participants reported more that the personal advice was interesting and instructive. Older participants also found more that the action plan was extra motivating; lower educated participants found the personal advice more instructive and motivating compared to higher educated participants; most adults found it positive that they could evaluate whether they reached their goals and indicated that this evaluation is extra motivating.  **Content**: Most adults thought that the personal feedback was relevant, interesting, clear, and understandable; a majority found that the advice was instructive, and motivating; 10.3% indicated the advice to be too prescriptive; participants who read extra information found it positive that they could choose which information they wanted to read; the extra information was evaluated as useful by a majority of the participants; most participants thought that the program contained good questions, and tips to make an action plan; most participants found it positive that they could make an if-then plan and most indicated to have enough information to make such a plan; Participants who completed the PA module, indicated there were too many questions compared to those that completed the fruit module. Participants who chose fruit, found the action plan more motivating than those who chose PA. More participants indicated to live up to their plan for fruit compared to participants that had a plan for PA.  **Efficiency**: 11.5% indicated that questions were too long and 5.8% found that there were too many questions. Only 5.9% found the personal feedback too long; those with lower education indicated more that the personal advice was too long.  **Understandability**: Most participants indicated the questions were easy to understand, had clear answering options, and had clear instructions. Younger participants (<40) found the answer options of the questionnaire more clear than older participants (>40). Participants with higher education reported more that the questions were easy to understand compared to participants with lower education.  **Visibility**: A large part of the participants indicated that the plan had an attractive lay-out, easy to understand, personally relevant, and extra motivating; the low educated group reported more that the action plan included all necessary things and indicated more that the lay-out was attractive than participants with higher education. | **Movement Behaviours:** The intervention proved to be able to increase PA in the short term (after 1 month). Mins/week of total PA (*p*<0.05) and mins/week moderate intensity PA (*p*<0.01) increased sig. more in the intervention group compared to control.  **(Other) Compliance:** High drop out for module 2 and 3; no sig. differences were found in the acceptability and feasibility questions about module 2 as a function of age and education level. | 1 |
| Plaete et al. (2015c) Qualitative Belgium | **Satisfaction**: The program was generally well accepted, including for participants with a low educational level and for older adults.  **Navigation**: Moving from one page to another was deemed as ‘too slow’.  **Understandability**: To make the program more comprehensible for the different groups, the questions, answer options, and advice were made shorter and clearer.  **Usability**: Most indicated that it was easy to use the intervention program on a tablet; a pen to tick the answers would be useful.  **Visibility**: Text was deemed to be too small to read on a tablet.  **Workflow**: Doubts were raised on how to implement MyPlan in general practice (e.g., in some situations it is not possible to use a tablet). | **Knowledge**: Providers appreciated that they did not need the expertise and time to compose personal advice for every adult accessing care, and many restrict their role to simply motivating and advising adults to use the intervention. | 5 |
| **PA Screen in EMR (*n* = 2)** | | | |
| Clark et al. (2020)  Qualitative  Canada | **Content**: Providers were receptive to an instrument that could provide customized information; Providers want prompts and messages that cater to a broad range of adults. Providers indicated that standardizing documentation of adult’s PA levels would improve their current practices but could make the process confrontational.  **Efficiency**: It was recommended that the tool should automatically calculate mins of PA.  **Understandability**: Simple questions were preferred; the terminology needs to be positive, easy to understand, and centred on adults accessing care.  **Usability**: Providers want a decision algorithm (e.g., “go to this guide, use this guide, etc.”) embedded in EMR. Adults accessing care should receive a paper handout and list of local resources. | **Knowledge**: Providers are not experts in PA; they need referral to or suggestions for adults to access resources made by experts. Providers have limited knowledge regarding PA, especially for individuals with chronic conditions or other barriers, therefore prompts and resources are needed to help providers have effective conversations.  **Ability**: Providers are willing to conduct PA screening in primary care, but they acknowledge they often do it poorly. | 5 |
| Neudorf et al. (2021) Qualitative Canada | **Content**: The need for the following was identified: additional diseases/conditions in the care plan, simple and non-intimidating prompting questions; evidence-based information and recommendations within care plan tool; disease-specific recommendations; care plan in printable format. The tool was seen to act as a placeholder for PA resources.  **Efficiency**: 2/5 participants asked the mock client if they participated in each of activities. The tool required too much thought to navigate the care plan. There were large amounts of text throughout the care plan; however, the length of the tool was reasonable.  **Navigation**: Providers expressed difficulty navigating the care plan and the transition from the questionnaire to care plan, and stated that navigation required thought. The pop-up link appeared in an inappropriate area (referring to intensity cues link). Function of the navigation guide was discovered by only 1/5 participants.  **Understandability**: Providers did not understand the purpose of the care plan, but thought the questionnaire was straightforward.  **Visibility**: Prompting questions were not visible throughout the care plan. The “calculate” link on the questionnaire and the open care plan tool link were missed repeatedly. Providers did not notice the user instructions for the navigation box in the care plan tool.  **Workflow**: The need for more comment boxes throughout the entire tool was brought up. Providers thought the tool required thought and attention to be used effectively as well as simple and non-intimidating prompts, and features to document adults’ responses (i.e., comment boxes, additional notes spaces, scheduling a follow-up appointment). | **Ability**: One participant indicated they would be quick at the tool after 2-3x using it. | 5 |
| **PAAT (Physical Activity Assessment Tool;** ***n* = 1)** | | | |
| Meriwether et al. (2006) Quantitative Non-randomized  USA |  | **(Other) Concurrent Validity:** The PAAT was significantly correlated with the International PA Questionnaire (*r*=0.562, *P*<0.001) and accelerometer (*r*=0.392, *P*=0.015) for MVPA. Seven-day test–retest reliability for MVPA was comparable for PAAT (*r*=0.618, *P*<0.001) and accelerometer (*r*=0.527, *P*<0.001).  **Criterion validity:** PAAT classified participants as “active” or “under-active” concordantly with accelerometer for 69.8% of participants and with IPAQ for 66.7%; strength of agreement was fair (κ=0.338 and 0.212, respectively). The PAAT classified fewer participants as active (60.3%) than either the accelerometer (71.4%; *P*=0.169) or IPAQ (80.3%; *P*<0.001), and measured PA more like the accelerometer than did IPAQ. | 2 |
| **PACE (Patient-Centered Assessment and Counselling for Exercise;** ***n* = 8)** | | | |
| Norris et al. (2000)  RCT  USA |  | **Knowledge**: Intervention providers perceived themselves to be more knowledgeable about PA counselling than did controls.  **Frequency**: Both intervention and control providers asked and counseled about PA more frequently, but the increase was sig. higher for intervention providers than for controls (*P*=0.001). Follow-up data for physicians showed that PACE-trained providers were 22% more likely than controls to counsel about PA at both health maintenance and chronic condition visits.  **Movement Behaviours:** At 6-month follow-up, the control group did not sig. differ from the intervention group for energy expended, time spent in walking or other moderate to vigorous activities, mental health, physical function, or behaviours previously shown to predict PA change. Among adults accessing care in the intervention, the stages-of-change score for contemplators sig. increased compared with controls, but without a sig. change in energy expended. | 4 |
| Prochaska et al. (2000) Qualitative USA | **Satisfaction**: 77% of providers and office staff would recommend PACE+ to other primary care offices; 45% of adults rated the computer program and printouts as helpful or very helpful in changing behaviour.  **Content**: 72% of adults believed the feedback fit them well or very well, suggesting that the tailoring including in PACE+ was good.  **Understandability**: 98% of adults understood the words and ideas.  **Visibility**: The need to improve the graphic interface was highlighted.  **Workflow**: 72% of providers and office staff reported being satisfied or very satisfied with how PACE+ was integrated into their office procedures. | **Confidence**: 85% of providers perceived the PACE+ program as helpful or very helpful for improving the PA behaviours of adults accessing their care. | 5 |
| Calfas et al. (2002)  RCT  USA | **Satisfaction**: Participants were highly satisfied with each component of the PACE+ intervention. Almost half rated both the computer and the provider counselling as helpful or very helpful in making changes in PA. The vast majority of participants endorsed PACE+ as a service that they would like offered at doctor's offices on an ongoing basis. Providers were satisfied with the program (mean score=4.1), would like to continue using the program (mean score=3.9), and would recommend to other providers (mean score=3.8) protocols and saw improvements in adults accessing their care.  **Content**: The majority of participants showed mailed materials to friends or family; more specific tailoring of the content of mailed materials may increase adults’ perception of their helpfulness in making behavioural changes. | **Movement Behaviours:** Adults who set a goal to increase moderate PA were more likely to progress to the next stage of change than those who did not set such a goal (*P*<0.001). Adults who targeted vigorous PA were more likely to progress to the next stage of change than adults who did not target that behaviour (*P*<0.001). | 3 |
| Bertozzi et al. (2004) Quantitative Descriptive  Italy | **Satisfaction**: There was difficulty maintaining elevated motivation in the physicians for an extended period of time.  **Workflow**: There was difficulty in joint action between physicians and trainers to facilitate the intervention. | **Movement Behaviours**: 64.1% declared to have increased their own PA level after the counselling intervention; meaningful increase of the motivational level (*P*<0.01).  **(Other) Motivation:** Physicians lacked the motivation to continue to use the tool.  **General Health:** Adults’ average BMI and WC had sig. decreased at follow-up (both *P*<.001) | 4 |
| Van Sluijs et al. (2005a) RCT Netherlands |  | **(Other) Self-efficacy:** Sig. positive effect of the intervention for both self-efficacy subscales (e.g., “making time for exercise” and “resisting relapse”) at the 8-week (T1) and 6-month (T2) follow-up (no difference from control). Sig. positive intervention effect on the behavioral processes of change was observed at all follow-ups; sig. effect on the cognitive processes of change was observed at T1 and T2. Sig. decrease of perceived barriers was observed at short-term follow-up (8 weeks), and a small and non-sig. decrease was observed at 6-month. | 4 |
| Van Sluijs et al. (2005b) RCT Netherlands |  | **Knowledge**: Most intervention providers did not tailor their counselling to individual stages of change but instead discussed the same topics with all adults accessing care.  **Movement Behaviours:** No sig. intervention effects over time were observed in the PA outcome measures. However, both groups exhibited an overall increase in duration of PA from baseline to 1-year follow-up.  **(Other) General Health:** WC sig. increased among intervention participants relative to control, but the overall study population exhibited a non-sig. decrease in weight over time. | 4 |
| Bolognesi et al. (2006)  RCT  Italy | **Efficiency**: Brief physician intervention to discuss PA did not need to take more than 3-5min during a visit, showing an ability to use the tool with minimal effort.  **Workflow**: The tool added to the regular clinic visit – the GP will often discuss quality of life anyway so that fits in naturally. | **Knowledge**: PACE is helpful because it minimizes the duration of intervention while improving the GPs knowledge.  **Ability**: PACE is helpful because it minimizes the duration of intervention while improving the GPs abilities.  **Movement Behaviours:** 75% of the adults who were physically active at baseline stayed active.  **(Other) Self-efficacy:** Participants in all stages increased their self-efficacy. Specific to the experimental group, self-efficacy increased significantly from the first to the second contact. The effect was stronger in male than in female participants.  **(Other) Stage of Change:** Over 50% of adults who either were not ready or ready (Stages 1 and 2) progressed, and no adult in these two stages of readiness regressed.  **(Other) General Health:** On average, active adults had a sig. decrease in BMI, whereas the inactive adults did not increase their BMI. Independent of exercise behaviour, the intervention group decreased in abdominal girth. | 2 |
| Spink et al. (2008) Quantitative Non-randomized Canada | **Content**: Enhanced treatment effect of additional telephone support was not supported. The content of the calls may require more specific tailoring for each participant so that the messages are congruent with the participant’s style of processing health information. | **Movement Behaviours:** After counselling, mean levels of self-reported PA for all participants sig. increased from 1.7 to 2.7 kcal/kg/day over a 1-month period (*p*<0.001); the frequency of PA of at least 10mins sig. increased; after counselling, participants engaged in moderate-intensity PA as frequently as light-intensity PA. | 4 |
| **PAFES (Physical Activity, Health, and Sports Plan; *n* = 1)** | | | |
| Gonzalez-Viana et al. (2018) Quantitative Descriptive Spain | **Satisfaction**: 42.4% of participants indicated that PAFES had helped to enhance PA in the community and 46.3% indicated that it had helped to improve their communication and collaboration with municipalities. | **Frequency:** 64.2% (*n*=99) indicated PAFES had been useful to increase PA screening, advice, and recording. PA screening of adults aged 15-69 years with at least one cardiovascular risk factor increased from 14.4% (*n*=280,162) in 2008 to 69.6% (*n*=1,355,818) in 2015, a 55.2% increase during that period. Among those screened as inactive, advice coverage increased from 8.3% (*n*=2,458) in 2012 to 35.6% (*n*=231,291) in 2015. 42.2% of respondents had received a PA recommendation from their provider in the previous year. By 2015, 6,046,611 (82.5%) of Catalan people had access to a PAFES “healthy route” in their municipality. In 2015, 100% of primary health care teams were included in the Plan. From 2005 on, there was a yearly increase in the number of primary health care teams implementing the Plan and registering PA screening and advice, except for 2011 when no training sessions were offered, and implementation and registration remained at the same level as in the previous year. By 2015 most primary health care teams (N 356; 96.4%) were recording their PA advice. In 2015, 18.2% (N 172) of the 947 municipalities of Catalonia had at least one “healthy route” and had identified their PA offerings (93.6% of the municipalities with >20,000 inhabitants and 60.8% of those with >5,000 inhabitants). Implementation: By the maintenance phase of the plan, >90% of primary health care teams were registering their PA screening and advice across all health regions.  **Movement Behaviours:** Of those advised by their provider to be active, 28.3% said they had followed their provider’s recommendation to walk 30 mins/day. Between 2006 and 2010, a general increase was observed in adults reporting moderate or high levels of PA, from 64.8% to 74.1% [adjusted OR=1.54; 95% CI (1.36-1.74), *p*<0.001]. From 2010 to 2013, PA levels declined, but remained higher than in 2006. In 2014, however, PA dropped below 2006 levels, returning to 2006 levels in 2015. After stratifying by sex and cardiovascular risk factors, a similar evolution in PA was observed over time; men without risk factors were the most active group and women with cardiovascular risk factors the least active. Women with no cardiovascular risk factors showed the highest PA increase between 2006 and 2010 (16.0%), followed by men and women with at least one cardiovascular risk factor (14.4% and 12.2%, respectively). | 4 |
| **PAHLS (*n* = 1)** | | | |
| Brostrom et al. (2017) Quantitative Descriptive Sweden |  | **(Other) Validity and Reliability:** Uni-dimensionality for the tool was supported by confirmatory factor analysis and Rasch analyses. Multiple group confirmatory factor analysis showed that the tool operated equivalently across both male and female participants. Internal consistency (Cronbach’s alpha 0.83) and composite reliability (0.89) were good. | 5 |
| **Paper-based Decision Tool (*n* = 1)** | | | |
| Cupples et al. (2018)  Mixed Methods Ireland | **Satisfaction**: Adults accessing care suggested that more feedback on their progress might increase their motivation for change.  **Content**: 16 adults thought they had enough advice and support to decide about change, but half of these were not ready for action. 14 wanted more information about practical options for change; only one provider thought that more details about heart disease should be provided.  **Efficiency**: The convenience of a paper-based tool was highlighted, but a need for alternative, electronic formats was recognised, particularly for adults with limited reading capability.  **Understandability**: Providers considered that the tool’s structured framework helped focus consultations on relevant issues.  **Visibility**: The tool's format, A5 size, content and brevity were welcomed unanimously.  **Workflow**: Opportunities to ask for explanations and converse with providers were valued. Participants' responses indicated the strength of impact of providers sharing personal experiences and showing understanding of adults’ lives. However, providers did not appear to recognize how much adults valued using the shared decision tool: they considered it impossible to use in everyday practice, given current time constraints and workloads. | **Knowledge**: Interviewees considered that no qualifications were needed to use the tool but interviewing skills were required and that someone, outside the practice team, could conduct consultations.  **Confidence**: Providers lacked confidence regarding their knowledge of lifestyle advice.  **Movement Behaviours**: 19 adults considered they could do more PA. At 1-month review some reported new activities but, for various reasons, did not sustain these. Television viewing was an indicator of sedentary behaviour, with 45% watching <1 hour's daytime television, but viewing time was higher in the evenings, on both weekdays and weekends, and changed little during the study. Sedentary behaviour fell over the 3 months, but there were also small reductions in mean MVPA and daily step counts. | 5 |
| **PAVS (Physical Activity Vital Sign; *n* = 6)** | | | |
| Greenwood et al. (2010) Quantitative Descriptive  USA |  | **(Other) General Health:** BMI sig. decreased 0.91 units for every day of moderate-intensity PA during a typical week (adjusted β coefficient; *P*<.001). BMI sig. decreased 2.90 units for those who reported PA ≥5 times in a typical week (adjusted β coefficient; *P*<.01) | 4 |
| Heath et al. (2015)  Quantitative Non-randomized  USA | **Satisfaction**: Providers were unified in their qualitative recommendation to integrate the Exercise is Medicine^®^ (EIM) protocols of PA assessment and referral into the EMR in response to questions about how to improve the EIM process.  **Usability**: 40% of providers reported that the EIM Health Care Provider Action Guide was easy to use, with 50% neutral. Use of the EIM materials was reported to be easy to very easy to use by more than 80% of respondents. | **Ability**: 53% of providers indicated that the use of the EIM protocols improved their counselling skills, while the group was split between feeling neutral and being helped by EIM in overcoming barriers for assessment and counselling about PA among adults accessing their care.  **Frequency**: Exposure to EIM protocols did not appear to impact the amount of time providers spent talking to about PA, with 30% (9/30) spending from 0–1 min, and 70% (21/30) spending >1min but <5mins.  **Movement Behaviours:** Almost 60% of providers were neutral as to whether the EIM process helped to increase adults’ PA, with 30% responding in the affirmative. PA assessments among the EIM + and the EIM only group revealed sig. greater increases from baseline to 3 months at follow-up in self-reported moderate, vigorous, and total PA.  **(Other) General Health:** Adults in the EIM+ group reported sig. fewer disability days compared to those in the EIM only group from baseline to follow-up at 3 months. Those in EIM+ reported a net decrease in the number of bad physical health days at follow-up compared with the EIM only. No sig. differences in number of bad mental health days. | 3 |
| Ball et al. (2015)  Quantitative Descriptive USA |  | **(Other) Validity:** The PAVS moderately strongly correlated with bout measures of PA and weakly correlated with non-bout measures of PA. Kappa statistics indicated that the PAVS agreed moderately with identifying if participants met or did not meet PA recommendations, whereas SNAP agreed poorly. The PAVS more strongly agrees with true PA when true PA is low and high. 91% respondents to the PAVS overestimated being sufficiently active by ≤3 days, and 9% overestimated being sufficiently active by 4-7. PAVS strongly identified insufficiently active people. | 3 |
| Ball et al. (2016a) Quantitative Descriptive USA |  | **(Other) Predictive Validity:** EHRs of the PAVS were strongly associated with BMI and disease burden. Sig. more clients who were overweight or obese (BMI≥25.0 kg·m-2) did not meet PA guidelines according to the PAVS compared with clients who were normal weight (BMI 18.5–24.9 kg·m2, *P*<.0001). Sig. more clients with the greatest disease burden (>50th percentile) also did not meet PA guidelines according to the PAVS compared with clients with less disease burden (*P*<.0001). No sig. differences were found in odds of clients being underweight (BMI<18.5 kg·m2), compared with normal weight (BMI 18.5–24.9), when not meeting PA guidelines according to the PAVS. Compared with normal weight, clients not meeting PA guidelines were sig. more likely to have a higher BMI. Clients not meeting PA guidelines were 3.7 times more likely to have a BMI≥40 kg·m2 compared with clients with a normal weight (*P*<.0001). Clients not meeting PA guidelines were 1.77x more likely to score 5 or more for the Charlson Comorbidity Index compared with clients with fewer than 5 points (*P*<.0001). Clients with the greatest odds of high disease burden when not meeting PA guidelines were female (OR=1.90, *P*<.0001) and those aged 30 to 39 years (OR=3.26, *P*<.0001). | 4 |
| Ball et al. (2016b)  Quantitative Descriptive USA |  | **(Other) Concurrent Validity:** PAVS agreed strongly with the Modifiable Activity Questionnaire (MAQ) 89.6% of the time and identified adults who were insufficiently active 89.6% of the time. PAVS showed moderate agreement for correctly identifying adults as meeting/not meeting the PA guidelines, when accounting for agreement occurring by chance (κ=0.55, *P*<.001). PAVS correlated strongly with MAQ for assessing weekly minutes of PA (*r*=0.71, *P*<.001). Adults who frequently self-identified as insufficiently active reported low confidence reporting PA to PAVS (70.9%), were not high school graduates (77.8%), and were female (63.2%). Agreement for identifying adults as meeting or not meeting PA guidelines was strongest for those with high confidence in reporting PA to PAVS (79.9% of the time; κ=0.60, *P*<.001) and for those >64 years of age (80.0% of the time; κ=0.60, *P*<.001). Those with high confidence in reporting PA to PAVS also had more valid measures of PA (i.e., stronger correlation between PAVS and MAQ; *r*=0.74, *P*<.001). More valid measures of PA were among men (*r*=0.81, *P*<.001) and those 18–64 years (*r*=0.75, *P*<.001). Bland-Altman agreement between PAVS and MAQ was fair. 95% confidence limits were wide (−371.3 to 198.7 mins/week). Participants reported an average of 86.3 fewer weekly minutes of PA to PAVS (128.5) compared with the MAQ (214.8; *P*<.001). Most felt “very sure” that their PA reported to PAVS was accurate (68%). Weekly minutes of PA assessed by PAVS strongly correlated with the same assessed by MAQ among the participants with high confidence self-reporting their PA (*r*=0.74) compared with low confidence (*r*=0.63, *P*=.01). | 5 |
| Birchfield et al. (2019)  Mixed Methods  USA | **Satisfaction**: 60% indicated they liked the idea of obtaining the PAVS and would recommend continuing this practice.  **Content**: One provider commented they were impressed with the quality of the messaging presented in the education materials and that they were a good way to reinforce their conversations about PA. All providers expressed that they would like to have electronic versions of the education materials integrated into the EMR system if they were going to continue to be used.  **Efficiency**: 2/5 thought the systems change was burdensome and created unnecessary work for the non-provider staff.  **Navigation**: The agreement that the charting options were important to have (i.e., record PAVS variables in EMR) but needed to be more user-friendly.  **Usability**: 60% found the Exercise Prescription forms useful as they made it easier to quantify and prescribe PA. One provider said the EIM materials and process was not useful because inactivity was not an issue for the population served. | **Knowledge**: 67% were able to identify the PA guidelines (no change pre- and post-implementation).  **Ability**: 60% indicated the EIM approach made it easier for them to engage with adults accessing care about PA and the Exercise Prescription form made it easier to quantify and prescribe PA. One provider indicated that it did not change the way in which they engaged with adults, but it did reinforce the way they have always thought of exercise *as* medicine.  **Confidence**: Before training and resources: 94% of providers were confident they could identify inactive adults, provide PA advice, and prescribe exercise. 89% of providers were confident to provide PA resources and referrals (no change pre- and post-implementation).  **Frequency**: Lack of time (78%) and feeling it would not change adults’ behaviours (44%) were barriers to using the tool. Study 1: Proportion of adults being asked about PA increased in all groups from phase 1-2 and 2-3. Proportion of adults receiving PA advice increased in EMR++ and EMR+ from phase 1-2, no change in EMR++ from phase 2-3, and decreased in EMR+ and EMR from phase 2-3. Proportion of adults receiving an Exercise Prescription was greatest in EMR++, and proportion of adults receiving PA resources and referral increased for EMR++ and EMR+ from phase 1-2 (resources increased in EMR+/decreased in EMR++, and referrals increased in EMR++ from phase 2-3). Likelihood of adults receiving PA advice was sig. greater for the EMR++ group (OR=6.0, 95% CI=1.7-20.7) and the EMR+ group (OR=11.6, 95% CI=3.2-41.9) compared to the referent (EMR) group. Models were non-sig. for receiving an Exercise Prescription, PA resources, or a PA referral. Provider group was not a sig. predictor of being asked about PA. Study 2: The EMR++ group reported the highest proportions for PAVS reviewed (47%), PA identified (32%), and PA advice (9%). The EMR group was the least likely to obtain the PAVS (χ2(2)=28.94, *P*<.001) compared to the EMR++ and EMR+ groups. The EMR++ group was the most likely to review the PAVS (χ2(2)=779.53, *P*<.001), identify PA (χ2(2)=493.28, *P*<.001), and provide PA advice (χ2(2)=140.90, *P*<.001) compared to the EMR+ and EMR groups. | 3 |
| Carey et al. (2021)  Mixed Methods  USA | **Satisfaction**: Care providers expressed a wide variety of willingness to adopt a PAVS into standard workflow in the ambulatory care setting.  **Workflow**: Among nurses, the primary hesitation was the logistics of adding another step to the rooming process. Among providers, there was generally more openness to adopt the PAVS into standard workflow. |  | N/A |
| **Pedometer Prescription in EMR (*n* = 1)** | | | |
| Barnes et al. (2013) Quantitative Non-randomized USA |  | **Frequency**: Between February 2010 and December 2010, 750 pedometers were prescribed by the 2 participating clinics. All physicians caring for adults accessing care wrote at least one pedometer prescription during the study period. Of the 750 prescriptions written, 603 were filled in the pharmacy (80% filled). | 0 |
| **PEM (Electronic Medical Prescription in Portugal) (*n* = 1)** | | | |
| Mendes et al. (2020) Quantitative Non-randomized Portugal |  | **Frequency**: From September 2017 to June 2019, 119,386 adults accessing care from the Portuguese PHC system had their PA assessed through the “SClínico Cuidados de Saúde Primários” platform. This number represents a proportion of 1736 per 100,000 users of the NHS (6,876,364 Portuguese citizens). These records were completed by 3715 medical doctors (60%), 2382 nurses (39%), and 86 registered dietitians and nutritionists (1%). Between December 2017 and June 2019, a total of 7957 adults received PA brief counselling using the “PEM—Prescrição Eletrónica Médica” PA guides, meaning that 94 per 100,000 residents in Portugal ≥15 years were reached. A total of 20,494 guides were delivered, the majority in paper format (less than 5% were sent by email). | 3 |
| **Pressure System Model (*n* = 1)** | | | |
| Katz et al. (2008)  RCT  USA |  | **Frequency**: At 12 months, intervention residents provided PA counselling 1.5x more than they did at baseline (*P*<0.05) compared with no sig. changes in the control group.  **Movement Behaviours:** At 6-month follow-up intervention, adults’ PA levels increased sig. from baseline (1.77 ± 0.84; *P*=0.0376) | 3 |
| **Prex (*n* = 3)** | | | |
| Aittasalo et al. (2006)  Mixed Methods Finland | **Satisfaction**: 10 physicians believed using Prex in the future.  **Content**” The majority of the interviewed adults accessing care reported that Prex served as a trigger to initiate PA. 9 adults felt that they would not have changed their PA habits without Prex.  **Efficiency**: Counselling with Prex took 5-10 min on average, but 8 physicians would have preferred 15 min.  **Usability**: All adults accessing care except one considered Prex as a worthwhile way of promoting PA in health care.  **Workflow**: Most physicians reported Prex as an acceptable counselling tool, being most applicable for health check and control visits. | **Ability**: All adults accessing care felt that Prex had been within their capabilities to carry out.  **Frequency**: The average number of Prex’s administered per physician was 5 (2 physicians compiled only 1 and 11 physicians 5–10 prescriptions). No blank Prex forms were returned, suggesting that Prex had been delivered to all the adults intended. PA habits had been assessed (100%), a PA goal had been set (78%) and a control visit had been agreed (87%). However, most of the goals were health-oriented (70%) and the average number of weekly PA sessions recommended (7.6) was quite high compared to adults’ prevailing sessions in Prex (3.7). 53% of the PA plans included only structured exercise and none of them were exclusively based on lifestyle activities. A control visit was in most cases the next physician’s visit (82%) but a pre-set date had been written down in only every other Prex. 4 physicians had used Prex as a referral to other health care staff or exercise experts.  **Movement Behaviours:** At 2-month follow-up, the increase in the weekly number of PA sessions was on average 1.0 (95% CI 0.0 to 2.0, *P*=0.05) session and in at least moderate-intensity PA 0.8 (95% CI 0.1 to 1.5, *P*=0.024) sessions greater in Prex than in control. At 6-month follow-up, the mean difference in changes between Prex and control in at least moderate-intensity PA was 0.9 weekly sessions (95% CI 0.2 to 1.5) for the favor of Prex.  **(Other) General Health**: At 2-months follow-up, 24% of the respondents reported some adverse effects caused by PA, mostly musculoskeletal pains, but no statistically sig. differences were found between the groups. The most common reasons for participants not carrying out Prex were lack of time and willpower. Factors encouraging to Prex included company, good feeling after PA, baby-sitter and good weather. | 3 |
| Aittasalo et al. (2007) Quantitative Descriptive  Finland | **Satisfaction**: The counselling approach was deemed credible and acceptable. It was justified due to its resemblance to drug prescriptions, was developed in conjunction with physicians in a pilot study, was based on the prevailing evidence on health-enhancing PA and PA counselling, and the compliance with the counselling principles was enhanced with a User’s Guide. In the further development of Prex, however, it was not as successful: Negotiations with the electronic record system producers were prolonged and finally abandoned due, according to the producers, to insufficient customer demand. | **Frequency**: The proportion of physicians asking at least "one out of three" of adults accessing their care about PA habits was 64.9% in 2002 and 66.8% in 2004 (statistically sig.). Similar changes in all subgroups except for the youngest age group and those working in private clinics and Occupational Health Care (changes are modest). The proportion of physicians using ‘Prex’ or other written material in PA counselling with at least 1/3 of adults accessing care was 12.2% in 2002 and 11.0% in 2004 [statistically sig. decline of 1.3% units (95%CI -2.0 to -0.5)]. Decrease seen in both genders and in the youngest and oldest age groups as well as in physicians working in Municipal Health Care (declines are modest from clinical point of view).  **(Other) Reach:** 3048 blocks of prescriptions were delivered by the end of 2004, 50% of them to Municipal Health Care, 15% to Occupational Health Care, 24% to local projects and 11% to hospitals, private clinics and rehabilitation centres. The number of Municipal Health Care requesting material was 96, representing 34% of all the centres. Occupational Health Care coverage was 7%.  **(Other) Implementation**: By the end of 2003, Prex had been introduced at 49 events involving altogether 3555 participants and in 8 articles published in professional journals. Referrals were made in 69 newspaper articles, 33 articles in health and exercise magazines, 8 articles in other magazines and 7 TV and 5 radio programmes. Collaboration was started with the 6 Finnish Centres of Exercise Medicine, who provide training to medical students together with the universities. 1480 information leaflets were mailed to physicians in hospitals, private clinics and rehabilitation centres. Programme costs 2001-2004 were 266,000 Euros.  **(Other) Maintenance**: By the end of 2004, at least 14 local projects had been initiated based on Prex to harmonize PA counselling practices in health care and to improve inter-sectoral cooperation. Prex was introduced as a recommended tool for PA counselling in 2 national public health documents [Recommendations for promoting health-enhancing PA (Ministry of Social Affairs and Health, 2001) and Programme for the Prevention of Type 2 Diabetes (Finnish Diabetes Association, 2003)]. The latter was implemented in 5/20 Finnish hospital districts from 2003–2007. | 3 |
| Aittasalo et al. (2016) Quantitative Non-randomized  Finland |  | **Knowledge**: At follow-up, knowledge increased 10-17% depending on the outcome variable, but none were sig.  **Frequency**: At baseline 40 % of the professionals reported they gave PA advice to at least 2/3 of adults accessing care. The record sheets on visits indicated similarly that PA was discussed 44 % of the time. Every other adult (54 %) recalled that PA issues had been brought up at the visit. However, only 1 % of discussions included the 4 issues considered essential to PA counselling. The corresponding proportion in the questionnaire to professionals was 7 %. At follow-up the proportion of professionals giving PA advice and the proportion of visits including PA discussions decreased statistically significantly 2 and 6 %-points, respectively. The other variables related to the implementation and quality of PA counselling did not change. At baseline, only 5% of professionals had used the tool during the past 2 weeks or in general and had not been used at any adult visit according to the record sheet. Still, 4% of adults recalled having received PAP during their visit. No provider and only 2% of external partners reported they had a mutual agreement in their working unit to use the tool in PA counselling. At follow-up, usage of PAP among providers increased 32% and agreement on using it in PA counselling increased 32%. Visits with the tool were 4% more than at baseline. (Adult and external partner results non-sig.). At baseline, the majority of providers reported referring adults accessing their care for PA advice to other providers in the centre (internal collaboration) or external partners (83 % and 84 %, respectively). Based on the record sheets on visits where the tool was not used as a form of referral at any of the visits. 67% of the external partners reported collaborating with the local health centre in health-related PA issues. At follow-up the use of PAP as providers’ internal referral had sig. increased 1%. No changes in other variables. At baseline 37% of health professionals reported always entering information on PA to the record system. Based on the record sheets, PA information was recorded to the system in 27% of the visits. At follow-up the proportion of providers, who reported that they always recorded information on PA to the electronic system, had increased 11% but the change was not statistically significant. Physicians entering PA issues to the electronic record system had increased 15%. | 2 |
| **RADI (Rapid Assessment Disuse Index; *n* = 3)** | | | |
| Shuval et al. (2014a)  Quantitative Descriptive  USA |  | **Frequency**: 10% of adults accessing care received general advice to decrease sitting time, in comparison with 53% receiving general PA counselling. None, however, received a written plan pertaining to sedentary behaviour whereas 14% received a written PA prescription. Only 2% were provided with specific strategies for sedentary behaviour change in comparison with 10% for PA change. | 4 |
| Shuval et al. (2014b)  Quantitative Descriptive  USA |  | **(Other) Reliability:** The test-retest reliability for the RADI domains (moving, stair climbing, and sitting) ranged from moderate-strong agreement. The cumulative RADI score showed a strong agreement and was directly correlated with sedentary time and inversely correlated with sedentary breaks and light PA; accuracy of the survey is fair. The tool exhibited similar reliability and validity traits to longer, more cumbersome questionnaires.  **(Other) General Health:** The cumulative RADI score was directly correlated with sedentary time (Spearman’s ρ=0.402; p<0.001) and inversely correlated with sedentary breaks (Spearman’s ρ=−0.425; p<0.001) and light activity (Spearman’s ρ=−0.406; p<0.001), while adjusting for accelerometer wear time, age, gender, race/ethnicity and BMI. RADI cumulative score was not sig. correlated with MVPA (accelerometer-derived). | 5 |
| Shuval et al. (2020) Quantitative Non-randomized  USA |  | **(Other) General Health:** Higher RADI sitting scores directly related to an increased likelihood for metabolic syndrome among women only (influenced by the association of the RADI sitting score with HDL levels). Among women, the cumulative RADI score was not sig. associated with metabolic syndrome; however, a transition to a higher RADI sitting score by 1 unit (vs remaining in the score) was related with a 1.4 and 1.3x higher odds for having low HDL cholesterol (95% CI, 1.05– 1.87) and metabolic syndrome (95% confidence interval, 1.02–1.64), respectively. No sig. relations were found among men. | 1 |
| **RAPA (*n* = 1)** | | | |
| Topolski et al. (2006) Quantitative Descriptive USA |  | **(Other) Criterion validity:** The RAPA was more highly correlated with moderate and total calories than either the BRFSS (t102=2.88, *P*<.005) or the PACE (t102=3.34, *P*<.001). **(Other) Sensitivity, Specificity, and Predictive Value:** The RAPA had the best sensitivity and negative predictive value of the 3 questionnaires.  **(Other) Discriminant Known Groups Validity:** The RAPA showed superior performance over the other 2 measures in being able to discriminate between those who reported inadequate vs. adequate moderate and vigorous PA. | 3 |
| **REAP (*n* = 1)** | | | |
| Gans et al. (2003) Quantitative Descriptive USA | N/A | N/A | N/A |
| **SNAP (*n* = 2)** | | | |
| Harris et al. (2005) Qualitative  Canada | **Satisfaction**: All GPs felt that overall, the SNAP interventions had a positive impact on adults accessing their care.  **Content**: Some GPs said that the 5 As approach was particularly useful because it prompted them to ask about SNAP risk factors, even when the adult presented for different reasons; the proportion of practices with display materials on PA increased after the intervention, as did the proportion with a designated staff member to coordinate education materials for adults accessing care. The motivational interviewing workshop was seen as helpful.  **Efficiency**: Difficulty making the organisational changes required to incorporate SNAP into the practice as a whole was identified. Lack of time and heavy workload were mentioned (SNAP intervention required proactive planning).  **Workflow**: Most of the GPs had incorporated SNAP into their existing management of adults with chronic diseases such as hypertension and diabetes, especially about assessing height, weight, BMI, smoking and alcohol consumption. This was especially true for adults with diabetes, for whom assessment was part of the annual cycle of care, and for adults having a care plan; overwhelming to fit into existing practice. There was a good fit between SNAP interventions and clinical practice. | **Knowledge**: Before the intervention, GPs' self-rated skills and knowledge in assessing and offering interventions to help adults accessing care reduce smoking and increase PA were already high.  **Ability**: All GPs felt that, overall, the SNAP interventions reinforced their clinical skills.  **Confidence**: GPs who had previously conducted SNAP interventions found participation and ensuring DGP support gave them encourage and confidence to sustain SNAP management. | 5 |
| Laws et al. (2010)  Mixed Methods Protocol  Australia | N/A | N/A | N/A |
| **Standardized Computer Software (GOAL Study; *n* = 3)** | | | |
| ter Bogt et al. (2011a)  RCT  The Netherlands |  | **(Other) General Health:** 60% of participants maintained weight loss after 3 years. No sig. difference in mean weight change and change of WC between groups. | 2 |
| ter Bogt et al. (2011b)  RCT  The Netherlands |  | **Movement Behaviour:** MVPA increased after 1 year and was higher in the NP group, with a sig. increase in walking compared to control (*P*=0.05). Lifestyle counselling that focused on weight maintenance led to an increase in walking in intervention group compared with usual care group. | 2 |
| Barte et al. (2012)  Quantitative Descriptive  The Netherlands | **Content**: 76% of adults accessing care totally agreed lifestyle goals were used. 74% totally agreed pedometer is useful. 81% agreed that the PA advice is useful; 61% agree it fit into their daily life; 55% agreed it added knowledge. |  | N/A |
| **Sleep Health Materials and Protocol (*n* = 1)** | | | |
| Fuller et al. (2011) Quantitative Descriptive  Australia | **Satisfaction**: Most adults accessing care felt positively about the program with 74% indicating that they would recommend the service to a friend. | **Knowledge**: Sleep health knowledge was sig. higher among pharmacists. 62% of adults accessing care followed up reported that the service had had some impact (defined as a little, some, or a large impact) on their understanding of good sleep health practices with only 23% reporting no impact at all.  **Confidence**: 59% of adults reported that the service had largely to somewhat impacted on their confidence in managing sleep problems, whilst 26% reported no impact.  **Frequency**: Pharmacists delivered a total of 847 interventions, with 418 cases of written information provision, 292 verbal counselling sessions, and 137 referrals to a physician. The mean total number of interventions delivered per pharmacist was 42.3 ± 53.3 (range 0–208). 46% of adults referred indicated that they had followed up on the referral provided to them by the pharmacist. | 5 |
| **STEP (*n* = 7)** | | | |
| Petrella & Wight (2000)  RCT  Canada | **Efficiency**: Most physicians in STEP and control said that the counselling did not significantly ‘slow’ their practice. | **Knowledge**: 100% of physicians using STEP believed the intervention improved their knowledge of exercise counselling, compared to 88 in the control (*P*=.009).  **Confidence**: 97% of physicians using STEP believed the intervention improved their confidence to discuss exercise, compared to 82% in the control (*P*=.01).  **Frequency**: 12/20 physicians in STEP vs. 7/20 in the control used the exercise prescription protocol in the previous month. | 3 |
| Petrella et al. (2003)  RCT  Canada |  | **(Other) Compliance:** 76% in STEP vs. 61% in control at 6-months (*P*<.05) and 71% vs 56% in control at 12-months (*P*<.05). **(Other) VO_2_ max**: 11% of STEP group sig. increased their VO_2_ max (21.3 to 24 ml/kg/min) compared to 4% (22 to 23 ml/kg/min) in the control at 6 months and 17% (21.3 to 24.9 ml/kg/min) vs 3% (22 to 22.8 ml/kg/min) at 12-months (*P*<.001). The STEP group reported sig. higher exercise self-efficacy (4.6 vs 6.8) compared to control at 12 months (*P*<.001), but there was a sig. increase from baseline in the control group (4.2 vs. 5.4; *P*< .05). **(Other) Clinical Measures:** 9-mmHg reduction in SBP (*P*=0.002) and a 7.4% reduction in BMI (*P*=0.05) observed in the STEP group compared to control at 12-months. Post-hoc subgroup analyses showed having >2 chronic disease conditions and having BMI >32 showed greatest improvements in VO_2_ max and exercise self-efficacy in STEP vs control. There was a sig. dose-dependent improvement in VO_2_ max for increasing level of participation (22.0 1.4; 23.6 1.1; 25.4 1.7 [*P*<.001]), but not exercise self-efficacy, with greater self-reported compliance according to exercise opportunities in both groups. | 4 |
| Petrella et al. (2010)  RCT  Canada |  | **(Other) pVO_2_ max:** increased 9.9% in STEPS group at 12-months vs. 7.1% in control group (both *P*<.001), but this was not sig. between the groups. Greatest improvement in pVO_2max_ for the STEPS group was in the first 3 months (6.6%); increases of 3.9% more at 6-months and 1.7% more at 9-months. Control group increased pVO_2_ max by 4.9% in the first 3 months, with an increase of 5.8% at 6 months, but a slight decline of 1.0% in the last 6 months. At 12 months, HRmax and RPE sig. increased (*P*<.01) in both the STEPS and control groups, but there were no differences between groups. Stepping time sig. decreased (*P*<.001) in both treatment groups, but STEPS group had a sig. greater decrease (*P*<.001). At 12 months the control groups’ mean HRrest had sig. increased (*P*<.05) and the STEPS group had a non-sig. increase. The STEPS group had a 4.1–mm Hg reduction (*P*<.001) in systolic blood pressures compared with a 0.4–mm Hg in the control group at 12 months. The DBPrest sig. decreased (*P*<.05) in both the STEPS and control groups. Self-reported total energy expenditure (kcal/kg/d) sig. increased in both the STEPS and control groups; however, the STEPS group had a sig. greater increase compared with the control group (2.1% and 0.8%, respectively; *P*=.006). When mean (SD) energy expenditure was adjusted for body weight (kcal/d), there was still a sig. increase (*p*<.001) at 12 months in the STEPS group [69.06 (169.87) kcal/d], whereas the control group had no sig. changes (–6.96 [157.06] kcal/d). | 0 |
| Knight et al. (2014a) Quantitative Non-randomized Canada |  | **Movement Behaviours:** No difference in changes between groups for PA (F(2247)=1.615, P=0.201, ηP2=0.013).  **(Other) General Health:** No difference in changes between groups for body weight (F(2247)=2.147, *P*=0.119, ηP2=0.017), or BP (SBP, F(2247) 1.260, *P*=0.286, ηP2 0.010; DBP, F(2247)=0.520, *P*=0.595, ηP2=0.004). Changes were sig. different between groups for fasting blood glucose (F(2247)=5.978, P=0.003, ηP2=0.046). Post-hoc analysis showed no sig. difference in change in blood glucose between the exercise and comprehensive counselling groups; however, there was a sig. difference between the SB and CC groups as well as the SB and exercise groups (*P*s<0.05). | 2 |
| Knight et al. (2014b) Quantitative Non-randomized Canada |  | **(Other) Clinical Measures:** Sig. differences from baseline to follow-up (F(5,50)=20.458, *P*=0.000, ηP2=0.672). Posthoc analysis indicated this difference was not sig. between groups. Statistically sig. changes from baseline to follow-up for all groups in BMI, WC, DBP, and VO_2_ max, with no change in SBP. Secondary: The difference in blood panel measures was sig. from baseline to follow-up (F(5,50)=4.576, *P*=0.002, ηP2=0.314). Post-hoc analysis indicated this difference was not sig. between groups. Statistically sig. changes from baseline to follow-up for all groups in fasting glucose, total cholesterol, and triglycerides, but no difference in HDL cholesterol or LDL cholesterol. | 2 |
| Knight et al. (2014c) Quantitative Descriptive  Canada |  | **(Other) VO_2_ max:** There was a strong relationship between predicted VO_2_ max from the STEP tool and direct measurements of VO_2_ max from the maximal treadmill test in the present study (*r*=.78, *P*<0.001), and the relationship remained strong for each sex (female, *r*=.79, *p*<0.001; male, *r*=.78, *P*<0.001). Previously reported correlation coefficients between VO_2_ max predicted using the STEP tool and measured directly via treadmill test ranged from *r*=.75 to .94. The Bland-Altman plot further demonstrated acceptable agreement between tests. Systematic bias was observed between VO_2_ max tests, indicating higher values in the predictive (STEP) vs. maximal test (mean difference=6.4 mL/kg/min; 95% CI=4.1 to 8.7 mL/kg/min, *P*<0.001). | 4 |
| Knight & Petrella (2014)  Mixed Methods Canada |  | **Knowledge**: Participants reported they gained an understanding of their personal PA behaviours relative to evidence-based guidelines. Participants felt the PA counselling process was a beneficial learning experience by enhancing their knowledge of and confidence with PA and exercise.  **Confidence**: Participants felt the PA counselling process was a beneficial learning experience by enhancing their knowledge of and confidence with PA and exercise.  **Movement Behaviours:** Most participants said they successfully incorporated healthy PA behaviours into their daily routines and most who continued their prescription adapted it to their evolving activity needs and preferences. Some participants reported obstacles to engaging in sustained activity behaviours post-intervention (common reasons: weather/seasons, lack of purpose after study ended, medical reasons).  **(Other) Aerobic Capacity:** Sig. change (F(2,38)=13.645, *P*=0.000, ηp2=0.418). Post-hoc analysis indicated the difference was sig. between pre-intervention (week 0) and post-intervention (week 12) visits. No difference between post-intervention and follow-up (after 36 weeks) visits (but there were still increases in VO_2_ max from post-intervention to follow-up). | 4 |
| **Step It Up! (*n* = 2)** | | | |
| Pears et al. (2016)  RCT  UK | **Satisfaction**: 6/12 providers favoured the Pedometer VBI, 5 favoured the Combined VBI, and one favoured the Motivational VBI.  **Content**: 37 adults accessing care reported that the Health Check was a good time to discuss PA and that the VBI was a good reminder of the importance of PA.  **Efficiency**: All providers felt that the Pedometer VBI was the easiest and quickest to deliver, and that delivery of the Combined VBI was most difficult and time-consuming.  **Usability**: 4 providers reported that ease and duration of VBI delivery depended more on the responsiveness of adults than on VBI content. | **Confidence**: All intervention groups reported a stronger intention to be more physically active than control participants. The 12 providers interviewed reported that they felt most confident delivering the Pedometer and Combined VBIs and that these two VBIs were the most acceptable to participants and were most likely to be effective.  **Movement Behaviours**: Accelerometer-derived measures of PA were similar for all VBI arms relative to control. Self-reported measures of PA were also similar for all VBI arms relative to control, except for reported leisure-based PA energy expenditure which was 50.3 % (95% CI: +2.1 %, +121.2 %) higher for Motivational VBI participants (16.5 kJ/kg/day) than Control participants (11.0 kJ/kg/day). Intervention effect ranged from -3.1 to +23.5 accelerometer counts/min.  **(Other) Cost:** £6.83 (US$10.06) per participant for the Motivational group (£4.99/US$7.35 delivery, £1.84/US$2.71 materials); £17.09 (US$25.17) for the Pedometer group (£3.67/US$5.41 delivery, £1.42/US$2.09 materials, £12/US$17.67 pedometer); and £20.98 (US$30.90) for the Combined group (£7.03/US$10.35 delivery, £1.95/US$2.87 materials, £12/US$17.67 pedometer). | 3 |
| Hardeman et al. (2020)  RCT  UK |  | **Frequency**: 491/505 adults received the intervention as planned. At 3-month follow-up, sig. more adults in intervention than control recalled receiving PA advice and intervention materials and reported enacting BCTs (e.g., using a pedometer to count steps) in daily life (*p*<0.001). 9/15 intervention components were delivered on average; components that were poorly delivered concerned the following BCTs: giving feedback on PA (51.4%), mentioning the effectiveness of pedometers (18.9%), and prompting goal setting (21.6%). Components to promote engagement were also poorly delivered, e.g., asking adults whether they were aware of the PA recommendations (29.7%) and whether they had any questions (18.9%). There was large variability in fidelity of delivery across practices and providers.  **Movement Behaviours:** Accelerometer counts/min at 3-month follow-up were similar in the control and intervention groups (660 versus 668 counts/min, respectively). This corresponds to an adjusted intervention effect of 8.8 counts/min (95% CI −18.7 to 36.3). Intervention effect ranged from +2.8 to +14.8; all values represented a positive non-sig. intervention effect with a robust main result. The intervention was associated with a reduction in PA among those at highest CVD risk and an increase in activity among those at lowest CVD risk (*P*=0.002). No sig. differences between groups in accelerometer-derived step counts per day and time spent in moderate or vigorous, vigorous, and moderate-intensity PA. Similarly, we found no sig. differences between groups in self-reported total PA, home-based activity, work-based activity, leisure-based activity, commuting PA, and screen or TV time.  **(Other) Cost:** £18.04 per participant (£11.25 pedometer, £4.67 face-to-face consultation time with nurse, £2.12 materials). Other costs were not statistically sig. different between groups (mean [SE]=+£21.55 [£24.21]). Total societal costs were not sig. different either (mean [SE]=+£53.46 [£76.97]). | 3 |
| **Tablet-based Lifestyle Assessment (*n* = 1)** | | | |
| Diaz et al. (2016) Quantitative Descriptive  USA | **Satisfaction**: Within the intervention group, participant satisfaction with regard to provider honesty and trust was higher when the provider discussed as least one of the weight-related healthier lifestyles with the individual. Almost 90% of adults accessing care said that they trusted their provider when counselling was provided, compared to 74% who trusted their provider but did not receive counselling.  **Content**: 93% of adults accessing care who received weight-related healthier lifestyle counselling said they felt their provider told the truth about their health compared to only 76% of individuals who did not receive counselling. | **(Other) General:** 55.2% were accurate at classifying whether their PA was healthy or unhealthy. Individuals in the intervention group were more likely to trust their providers and feel that their providers cared as much about their health as they do. | 5 |

* BCT = behaviour change technique; BMI = body mass index; BP = blood pressure; CI = confidence interval; CVD = cardiovascular disease; DBP = diastolic blood pressure; DBP_rest_ = resting diastolic blood pressure; EHR/EMR = electronic health/medical record; GP = General Practitioner; HDL = high-density lipoproteins; Hg = hemoglobin; HR = heart rate; HR_max_ = heart rate maximum; HR_rest_ = resting heart rate; IQR = inter-quartile range; LDL = low-density lipoproteins; NP = Nurse Practitioner; OR = odds ratio; PA = PA; QALY = quality adjusted life years; QOL = quality of life; RCT = randomized controlled trial; RPE = rate of perceived exertion; sig. = significant; SBP = systolic blood pressure; SD = standard deviation; SE = standard error; VBI = very brief intervention; VO_2_ max = maximal oxygen uptake; WC = waist circumference
