# Supplementary material for: Tools to guide clinical discussions on physical activity, sedentary behaviour, and/or sleep for health promotion between primary care providers and adults accessing care: a scoping review
Source: BMC Prim Care. 2023 Jul 7;24:140. doi: 10.1186/s12875-023-02091-9 (PMC10326959; doi:10.1186/s12875-023-02091-9)
Supplement: Supplementary file 7 — Additional file 7: Mixed Methods Appraisal Tool (MMAT) Quality Assessment Ratings. [file 12875_2023_2091_MOESM7_ESM.docx]

**Multimedia Appendix 7.** Mixed Methods Appraisal Tool (MMAT) Quality Assessment Ratings.*

| **Author Information** | **Quality Appraisal Rating** | | | | |  |
| --- | --- | --- | --- | --- | --- | --- |
| **Qualitative**  **(*n* = 21)** | **1**  Is the qualitative approach appropriate to answer the research question? | **2**  Are the qualitative data collection methods adequate to address the research question? | **3**  Are the findings adequately derived from the data? | **4**  Is the interpretation of results sufficiently substantiated by data? | **5**  Is there coherence between qualitative data sources, collection, analysis and interpretation? | **Quality Appraisal Total** |
| Ogunleye et al. (2015) | Yes | Yes | Yes | Yes | Yes | 5 |
| Asselin et al. (2017) | Yes | Yes | Yes | Yes | Yes | 5 |
| Noel et al. (2018) | Yes | Yes | Yes | Yes | Yes | 5 |
| Dickfos et al. (2015) | Yes | Yes | Yes | Yes | Yes | 5 |
| Lin & Mann (2012) | Unclear | Unclear | Unclear | Unclear | No | 0 |
| Sopcak et al. (2017) | Yes | Yes | Yes | Yes | Yes | 5 |
| Sturgiss & Douglas (2016) | Yes | Yes | Yes | Yes | Yes | 5 |
| Carlfjord et al. (2012) | Yes | Yes | Yes | Yes | Yes | 5 |
| Sciamanna et al. (2004) | Yes | Yes | Unclear | No | Unclear | 2 |
| Mateo et al. (2018) | Yes | Yes | Yes | Yes | Yes | 5 |
| Mishuris et al. (2016) | Yes | Yes | Yes | Yes | Yes | 5 |
| Patel et al. (2011) | Yes | Yes | Yes | Yes | Yes | 5 |
| Patel et al. (2012) | Yes | Yes | Yes | Yes | Yes | 5 |
| Albert (2020) | Yes | Yes | Yes | Yes | Yes | 5 |
| Fransen et al. (2008) | Yes | Yes | Yes | Yes | Yes | 5 |
| Plaete et al. (2015a) | Yes | Yes | Yes | Yes | Yes | 5 |
| Plaete et al. (2015c) | Yes | Yes | Yes | Yes | Yes | 5 |
| Clark et al. (2020) | Yes | Yes | Yes | Yes | Yes | 5 |
| Neudorf et al. (2021) | Yes | Yes | Yes | Yes | Yes | 5 |
| Prochaska et al. (2000) | Yes | Yes | Yes | Yes | Yes | 5 |
| Harris et al. (2005) | Yes | Yes | Yes | Yes | Yes | 5 |
| **Quantitative**  **(Randomized Control Trial)**  **(*n* = 35)** | **1**  Is randomization appropriately performed? | **2**  Are the groups comparable at baseline? | **3**  Are there complete outcome data? | **4**  Are outcome assessors blinded to the intervention provided? | **5**  Did the participants adhere to the assigned intervention? | **Quality Appraisal Total** |
| Osunlana et al. (2015) | Yes | Unclear | Yes | Yes | Unclear | 3 |
| Campbell-Scherer et al. (2019) | Yes | Yes | Yes | Yes | No | 4 |
| Parekh et al. (2014) | Yes | Yes | Yes | No | No | 3 |
| Albright et al. (2000) | Unclear | Unclear | Unclear | Unclear | Unclear | 0 |
| Simons-Morton et al. (2001) | Yes | Yes | Unclear | No | Yes | 3 |
| Anderson et al. (2005) | Yes | Yes | Unclear | No | Yes | 3 |
| King et al. (2006) | Yes | Yes | Unclear | No | Yes | 3 |
| Mann et al. (2016) | Yes | Yes | Unclear | No | Yes | 3 |
| Nanchahal et al. (2009) | Yes | Yes | Yes | Unclear | Yes | 4 |
| Redfern et al. (2020) | Yes | Yes | Yes | No | Yes | 4 |
| Agarwal et al. (2020) | Yes | Yes | Yes | No | Unclear | 3 |
| Viglione et al. (2019) | Yes | Yes | Unclear | No | Yes | 3 |
| Kerse et al. (2005) | Yes | Yes | Unclear | Unclear | Yes | 3 |
| Lawton et al. (2009) | Yes | Yes | Unclear | Yes | No | 3 |
| Elley et al. (2011) | Yes | Yes | Unclear | Yes | No | 3 |
| Kolt et al. (2012) | Yes | Yes | Unclear | Yes | No | 3 |
| Patel et al. (2013a) | Yes | Yes | Unclear | Yes | No | 3 |
| Mehring et al. (2013) | Yes | Yes | Unclear | Yes | No | 3 |
| Abu-Saad et al. (2019) | Yes | Yes | Unclear | Yes | No | 3 |
| van der Weegen et al. (2015) | Yes | Yes | Yes | Yes | No | 4 |
| Ryu et al. (2017) | Unclear | Yes | Yes | Unclear | Yes | 3 |
| Plaete et al. (2015b) | Yes | No | No | No | No | 1 |
| Norris et al. (2000) | Yes | Yes | Yes | Unclear | Yes | 4 |
| Calfas et al. (2002) | Yes | Yes | Unclear | Unclear | Yes | 3 |
| Van Sluijs et al. (2005a) | Yes | Yes | Yes | Unclear | Yes | 4 |
| Van Sluijs et al. (2005b) | Yes | Yes | Yes | Unclear | Yes | 4 |
| Bolognesi et al. (2006) | Yes | No | Unclear | No | Yes | 2 |
| Katz et al. (2008) | Yes | Yes | No | No | Yes | 3 |
| Ter Bogt et al. (2011a) | Yes | Yes | Unclear | No | Unclear | 2 |
| Ter Bogt et al. (2011b) | Yes | Yes | Unclear | No | Yes | 3 |
| Petrella & Wight (2000) | Yes | Yes | Yes | Unclear | No | 3 |
| Petrella et al. (2003) | Yes | Yes | Unclear | Yes | Yes | 4 |
| Petrella et al. (2010) | Unclear | No | Unclear | Unclear | No | 0 |
| Pears et al. (2016) | Yes | Yes | No | Yes | Unclear | 3 |
| Hardeman et al. (2020) | Yes | Yes | Yes | Unclear | No | 3 |
| **Quantitative**  **(Non-Randomized)**  **(*n* = 15)** | **1**  Are the participants representative of the target population? | **2**  Are measurements appropriate regarding both the outcome and intervention (or exposure)? | **3**  Are there complete outcome data? | **4**  Are the confounders accounted for in the design and analysis? | **5**  During the study period, is the intervention administered (or exposure occurred) as intended? | **Quality Appraisal Total** |
| Matoff-Stepp (2012) | Unclear | Yes | Unclear | No | Yes | 2 |
| Lv et al. (2017) | Unclear | Yes | Unclear | No | Unclear | 1 |
| Grant et al. (2013) | Yes | Yes | Unclear | No | Yes | 3 |
| Sassano (2004) | Unclear | Unclear | Unclear | No | Unclear | 0 |
| Heath et al. (2015) | Yes | Yes | Unclear | No | Yes | 3 |
| Porter et al. (2002) | Unclear | Yes | No | No | Unclear | 1 |
| VanDenToorn (2016) | Unclear | Yes | Unclear | No | Unclear | 1 |
| Meriwether et al. (2006) | Unclear | Yes | Yes | No | No | 2 |
| Spink et al. (2008) | Yes | Yes | Yes | Unclear | Yes | 4 |
| Barnes et al. (2013) | Unclear | Unclear | Unclear | Unclear | Unclear | 0 |
| Mendes et al. (2020) | Yes | Yes | Unclear | Yes | Unclear | 3 |
| Aittasalo et al. (2016) | Unclear | Yes | No | No | Yes | 2 |
| Shuval et al. (2020) | No | Unclear | No | Unclear | Yes | 1 |
| Knight et al. (2014a) | Yes | Yes | Unclear | No | Unclear | 2 |
| Knight et al. (2014b) | Yes | Yes | Unclear | No | Unclear | 2 |
| **Quantitative**  **(Descriptive)**  **(*n* = 34)** | **1**  Is the sampling strategy relevant to address the research question? | **2**  Is the sample representative of the target population? | **3**  Are the measurements appropriate? | **4**  Is the risk of nonresponse bias low? | **5**  Is the statistical analysis appropriate to answer the research question? | **Quality Appraisal Total** |
| Burr et al. (2020) | Yes | Yes | Yes | Yes | Yes | 5 |
| Eakin et al. (2004) | Yes | Yes | Yes | No | Yes | 4 |
| Aubrey-Bassler et al. (2019) | Yes | Yes | Yes | No | Yes | 4 |
| Carlfjord et al. (2009) | Yes | Yes | Yes | N/A | Yes | 4 |
| Hessler et al. (2019) | Yes | Yes | Yes | Unclear | Yes | 4 |
| Resnick et al. (2008b) | Yes | Yes | Yes | Unclear | Yes | 4 |
| Elley et al. (2014) | Yes | Unclear | Yes | No | Yes | 3 |
| Goodyear-Smith et al. (2013) | Yes | Yes | Yes | Yes | Yes | 5 |
| Dedier et al. (2014) | Unclear | Unclear | Unclear | Unclear | Unclear | 0 |
| Kuntz et al. (2021) | Yes | Yes | Yes | No | Yes | 4 |
| Coleman et al. (2012) | Yes | Yes | Yes | Yes | Yes | 5 |
| Liu et al. (2017) | Yes | Yes | Yes | Unclear | Yes | 4 |
| Ahmad et al. (2015) | Yes | Yes | Yes | Unclear | Yes | 4 |
| Dalziel et al. (2006) | Unclear | Unclear | Yes | Unclear | Yes | 2 |
| Sinclair & Hamlin (2007) | No | Unclear | Yes | Unclear | No | 1 |
| Leung et al. (2012) | Yes | Unclear | Yes | Yes | Yes | 4 |
| Patel et al. (2013b) | Yes | Unclear | Yes | Unclear | Yes | 3 |
| Hamlin et al. (2016) | Yes | Unclear | Yes | Unclear | Yes | 3 |
| Murphy et al. (2015) | Yes | Yes | Yes | Unclear | Yes | 4 |
| Yamane et al. (2020) | Yes | No | Yes | Unclear | Yes | 3 |
| Bertozzi et al. (2004) | Yes | Yes | Yes | No | Yes | 4 |
| Gonzalez-Viana et al. (2018) | Yes | Yes | Yes | Unclear | Yes | 4 |
| Brostrom et al. (2017) | Yes | Yes | Yes | Yes | Yes | 5 |
| Greenwood et al. (2010) | Yes | Yes | Yes | Unclear | Yes | 4 |
| **Ball et al. (2015)** | Yes | Unclear | Yes | Unclear | Yes | 3 |
| Ball et al. (2016a) | Yes | Yes | Yes | Unclear | Yes | 4 |
| Ball et al. (2016b) | Yes | Yes | Yes | Yes | Yes | 5 |
| Aittasalo et al. (2007) | Yes | Unclear | Yes | Unclear | Yes | 3 |
| Shuval et al. (2014a) | Yes | Yes | Yes | Unclear | Yes | 4 |
| Shuval et al. (2014b) | Yes | Yes | Yes | Yes | Yes | 5 |
| Topolski et al. (2006) | Yes | No | Yes | Unclear | Yes | 3 |
| Fuller et al. (2011) | Yes | Yes | Yes | Yes | Yes | 5 |
| Knight et al. (2014c) | Yes | Yes | Yes | Unclear | Yes | 4 |
| Diaz et al. (2016) | Yes | Yes | Yes | Yes | Yes | 5 |
| **Mixed Methods**  **(*n* = 11)** | **1**  Is there an adequate rationale for using a mixed methods design to address the research question? | **2**  Are the different components of the study effectively integrated to answer the research question? | **3**  Are the outputs of the integration of qualitative and quantitative components adequately interpreted? | **4**  Are divergences and inconsistencies between quantitative and qualitative results adequately addressed? | **5**  Do the different components of the study adhere to the quality criteria of each tradition of the methods involved? | **Quality Appraisal Total** |
| Flocke et al. (2006) | Yes | Yes | No | Unclear | No | 2 |
| Becker et al. (2011) | Yes | Yes | Yes | Yes | Yes | 5 |
| Carlfjord et al. (2010) | Yes | Yes | Yes | Unclear | Unclear | 3 |
| Verwey et al. (2012) | Yes | Yes | Yes | Yes | Unclear | 4 |
| Verwey et al. (2014) | Yes | Yes | Yes | Unclear | No | 3 |
| Verwey et al. (2016a) | Yes | Yes | Yes | Unclear | Yes | 4 |
| Foucher-Urcuyo et al. (2017) | Yes | Yes | Yes | Yes | Yes | 5 |
| Cupples et al. (2018) | Yes | Yes | Yes | Yes | Yes | 5 |
| Birchfield et al. (2019) | Yes | Yes | Unclear | No | Yes | 3 |
| Aittasalo et al. (2006) | Yes | No | Yes | Yes | Unclear | 3 |
| Knight & Petrella (2014) | Yes | Yes | Yes | Unclear | Yes | 4 |

***** Only scores of similar study design should be compared against each other.
